# The Copper Reduction Potential Determines the Reductive Cytotoxicity: Relevance to the Design of Metal–Organic Antitumor Drugs

**DOI:** 10.3390/molecules29051032

**Published:** 2024-02-27

**Authors:** Elena K. Beloglazkina, Anna A. Moiseeva, Sergey A. Tsymbal, Dmitry A. Guk, Mikhail A. Kuzmin, Olga O. Krasnovskaya, Roman S. Borisov, Elena S. Barskaya, Victor A. Tafeenko, Victoria M. Alpatova, Andrei V. Zaitsev, Alexander V. Finko, Valentina A. Ol’shevskaya, Alexander A. Shtil

**Affiliations:** 1 Department of Chemistry, Lomonosov Moscow State University, 1/3 Leninskie Gory, Moscow 119991, Russia; bel@org.chem.msu.ru (E.K.B.); moiseeva@org.chem.msu.ru (A.A.M.); dmh200949@gmail.com (D.A.G.); michael-06-95@mail.ru (M.A.K.); krasnovskayao@gmail.com (O.O.K.); elenakovaleva2010@gmail.com (E.S.B.); tafeenko-victor@yandex.ru (V.A.T.); finko.alexander@gmail.com (A.V.F.); 2 International Institute of Solution Chemistry and Advanced Materials and Technologies, ITMO University, 9 Lomonosov Street, Saint-Petersburg 197101, Russia; zimbal@scamt-itmo.ru; 3 Topchiev Institute of Petrochemical Synthesis, Russian Academy of Sciences, 29 Leninsky Avenue, Moscow 119991, Russia; borisov@ips.ac.ru; 4 A.N.Nesmeyanov Institute of Organoelement Compounds, Russian Academy of Sciences, Bld. 1, 28 Vavilov Street, Moscow 119334, Russia; vika.alpatova@gmail.com (V.M.A.); porphyrin@yandex.ru (A.V.Z.); olshevsk@ineos.ac.ru (V.A.O.); 5 Blokhin National Medical Research Center of Oncology, 24 Kashirskoye Shosse, Moscow 115522, Russia

**Keywords:** copper, redox reactions, electrochemistry, tumor cells, cytotoxicity, medicinal chemistry, antitumor drug design

## Abstract

Copper–organic compounds have gained momentum as potent antitumor drug candidates largely due to their ability to generate an oxidative burst upon the transition of Cu^2+^ to Cu^1+^ triggered by the exogenous-reducing agents. We have reported the differential potencies of a series of Cu(II)–organic complexes that produce reactive oxygen species (ROS) and cell death after incubation with *N*-acetylcysteine (NAC). To get insight into the structural prerequisites for optimization of the organic ligands, we herein investigated the electrochemical properties and the cytotoxicity of Cu(II) complexes with pyridylmethylenethiohydantoins, pyridylbenzothiazole, pyridylbenzimidazole, thiosemicarbazones and porphyrins. We demonstrate that the ability of the complexes to kill cells in combination with NAC is determined by the potential of the Cu^+2^ → Cu^+1^ redox transition rather than by the spatial structure of the organic ligand. For cell sensitization to the copper–organic complex, the electrochemical potential of the metal reduction should be lower than the oxidation potential of the reducing agent. Generally, the structural optimization of copper–organic complexes for combinations with the reducing agents should include uncharged organic ligands that carry hard electronegative inorganic moieties.

## 1. Introduction

The plasma membrane, as well as the membrane organelles, are the critical guardians of cell integrity, since their damage leads to nonrepairable death. Therefore, membranes are considered important drug targets for intractable tumors. In particular, cells resistant to a variety of chemotherapeutic drugs remain sensitive to necrosis-inducing strategies [1,2,3]. This approach can be accomplished via the peroxidation of membrane lipids by reactive oxygen species (ROS) generated by direct pharmacological prooxidants or indirectly as a result of metabolic oxidation by drug combinations. In the latter scenario, copper-containing organic complexes are especially attractive because of the exceptional ability of this metal to be reduced with subsequent ROS generation.

Investigating the antitumor potential of copper–organic compounds in combinations with reducing agents, we have demonstrated that Cu(II) complexes readily triggered membrane damage and death of wild type and multidrug-resistant human tumor cells upon reduction of the Cu(II) cation by *N*-acetylcysteine (NAC) or ascorbate [4]. The remarkable antitumor efficacy of individual complexes has been mechanistically attributed to ROS generation upon the reduction of Cu^2+^ to Cu^1+^. Importantly, the cytocidal potencies varied depending on the molecular context around the metal cation. In contrast to the most active [(*Z*)-3-(2-fluorophenyl)-2-methylthio-5-(pyridin-2-ylmethylene)-3,5-dihydro-4*H*-imidazol-4-one]copper(II) dichloride, the complexes in which Cu(II) was coordinated by the tetrapyrrolic macrocycle were substantially less potent when combined with NAC or ascorbate, suggesting that the organic scaffold can impede Cu(II)-to-Cu(I) transition. Therefore, the design of drug candidates requires the criteria of the ability of the Cu–organic complex to be activated by reducing agents. One important issue is the role of the organic ligand’s spatial structure in reduction of the copper cation.

The search for cytotoxic coordination compounds of biogenic metals has attracted significant interest [5,6,7]. Among the biogenic transition metals, the most promising may be copper coordination compounds, which can exhibit high cytotoxicity via various mechanisms [8,9,10,11,12]. Copper-containing complexes with 2-thio-imidazol-4-ones are among the compounds with antitumor activity [13,14] and the ability to inhibit telomerase activity [15]. Furthermore, mononuclear Cu^+2^ coordinated 2-thio-imidazol-4-ones showed ROS-independent nuclease activity and apoptosis induction in MCF-7 breast carcinoma spheroids [15]. Thiosemicarbazones and their copper complexes also demonstrated the antitumor potency. Bis-thiosemicarbazoneelesclomol demonstrated clinical efficacy in leukemia [16,17]. Elesclomol exerted its effect by forming a Cu(II) coordination compound that caused intracellular oxidative stress due to redox reaction Cu(II)/Cu(I), the disruption of mitochondrial respiration and ROS formation. Ultimately, the coordination of copper with the elesclomol ligand interfered with the energy metabolism and triggered mitochondrial apoptosis in tumor cells [18]. At submicromolar concentrations, the elesclomol–copper complex showed antiproliferative activity against six gynecological cancer cell lines. Recently, Anjum et al. reported eight different thiosemicarbazido-based Cu(II) complexes with promising antitumor activity [19].

Porphyrins and their derivatives are widely recognized as antitumor drug candidates due to accumulation in tumors and broad opportunities for chemical modifications of the macrocyclic ring and its periphery [20,21,22,23,24]. In particular, a complexation of porphyrins with copper yielded the coordination compounds with high cytotoxic activity [25,26,27,28,29,30].

**Figure 1:**
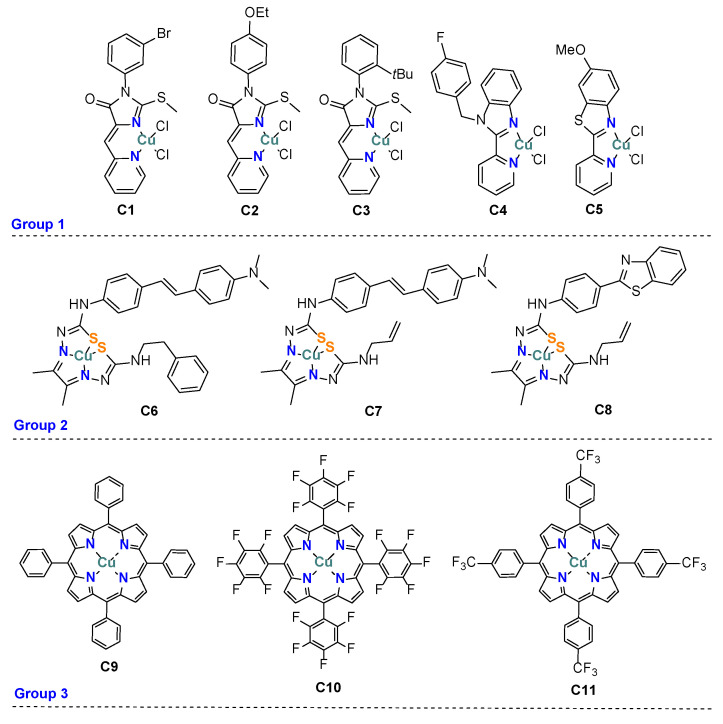
Copper–organic complexes **C1**–**C11** investigated in this study.

In the present study, we explored three chemo types of Cu(II)–organic complexes containing ligands of various structural types and geometry of the copper coordination environment. The ligands were pyridylmethylenethiohydantoins **C1**–**C3**, pyridylbenzimidazole **C4**, pyridylbenzothiazole **C5**, thiosemicarbazones **C6**–**C8** and porphyrins **C9**–**C11** (Figure 1). We demonstrated that the ability of NAC to reduce copper (II) in these complexes, a property translated into the cytotoxic potency in the cell culture, is determined by the potential of the Cu^+2^ → Cu^+1^ redox transition. Most importantly, the reduction potential in the complex must be lower than the oxidation potential of the reducing agent. In contrast, the geometry of the copper coordination environment had no significant effect. 

## 2. Results and Discussion

### 2.1. Synthesis of Coordination Compounds

**Scheme 1:**
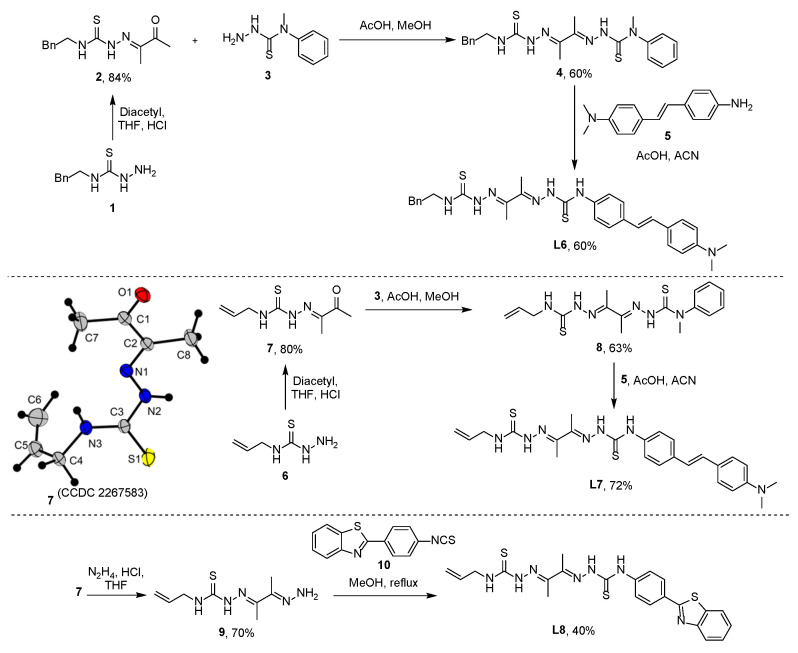
Synthesis of thiosemicarbazone ligands **L6**–**L8** and the crystal structure of **7** (CCDC 2267583). Thermal ellipsoids are at a 50% level.

Copper complexes **C1**–**C5**, **C9**–**C11** and thiosemicarbazone ligands **L6**–**L8** were obtained as described in [31,32,33,34,35] and Scheme 1.

Thiosemicarbazides—in particular, *N*-benzylhydrazinecarbothioamide (**1**) and *N*-allylhydrazinecarbothioamide (**6**)—were obtained in high yields from respective isothiocyanates in the reaction with hydrazine [36,37]. Also, *N*-benzylhydrazinecarbothioamide (**1**) and *N*-methyl-*N*-phenylhydrazinecarbothioamide (**3**) were obtained via the nucleophilic substitution of dithiocarbamic acid esters [38,39]. The nucleophilic substitution of *N*-phenyl-*N*-methylthiocarbamoylimidazole with hydrazine led to compound **3** [40]. Diacetyl, like many 1,2-dicarbonyl compounds, is very reactive towards the primary amino group. In the presence of an acidic catalyst, diacetyl reacts with thiosemicarbazide derivatives at the primary amino group, forming symmetrical bis(diacetyl)thiosemicarbazone derivatives [41]. Also, the condensation reaction can be carried out at one carbonyl group of diacetyls by slowly adding the solution of a thiosemicarbazide derivative to a cooled solution of an excess of diacetyl (1.1–5 eq.) in the presence of an acid catalyst [42,43]. This method was used to obtain (*E*)-*N*-benzyl-2-(3-oxobutan-2-ylidene)hydrazine-1-carbothioamide (**2**) and (*E*)-*N*-allyl-2-(3-oxobutan-2-ylidene)hydrazine-1-carbothioamide (**7**). The crystal structure of **7** is shown in Scheme 1 and Figure S2. The order of the addition of the components is not that important as maintenance of the reaction temperature [44]. During the reaction, the formation of the cyclization byproduct 2,3-butanedione mono(4-*R*-thiosemicarbazone) is possible [45]. 

Compound **5** was obtained by reducing the corresponding nitro derivative with Sn(II) chloride [46,47] or Fe(II) sulfate [48]. Unsymmetrical bis-diacetylthiosemicarbazones were synthesized from the 2,3-butanedione mono(thiosemicarbazone) derivative and thiosemicarbazide. The reactions in dry DMF proceeded in the presence of catalytic amounts of acetic acid [49], in ethanol in the presence of a HCl catalyst [42] or with AcOH [47] and in tetrahydrofuran with AcOH [50]. The resulting unsymmetrical derivatives of bis(diacetithiosemicarbazone) can be modified by a nucleophilic substitution at one of the thiocarbonyl groups [47,51]. This strategy was undertaken for the synthesis of **4**, **8**, **L6** and **L7**. However, this method did not work for the synthesis of **L8**. An alternative strategy (Scheme 1) included the treatment of **7** with hydrazine hydrate in THF with catalytic amounts of HCl to produce **9** in an acceptable yield. From 4-(benzo[*d*]thiazol-2-yl)aniline, 2-(4-isothiocyanatophenyl)benzo[*d*]thiazole (**10**) was obtained with thiophosgene [52]. Compound **10** could also be synthesized by decomposition of the corresponding dithiocarbamate with Boc_2_O [53], although the yield was <50%, with a large amount of byproducts. Compound **9** easily attached to the isothiocyanate group at the primary amino group in **10** to produce **L8** [54,55]. See Figure S3 and other details in the Supplementary Materials.

### 2.2. Electrochemistry

**Figure 2:**
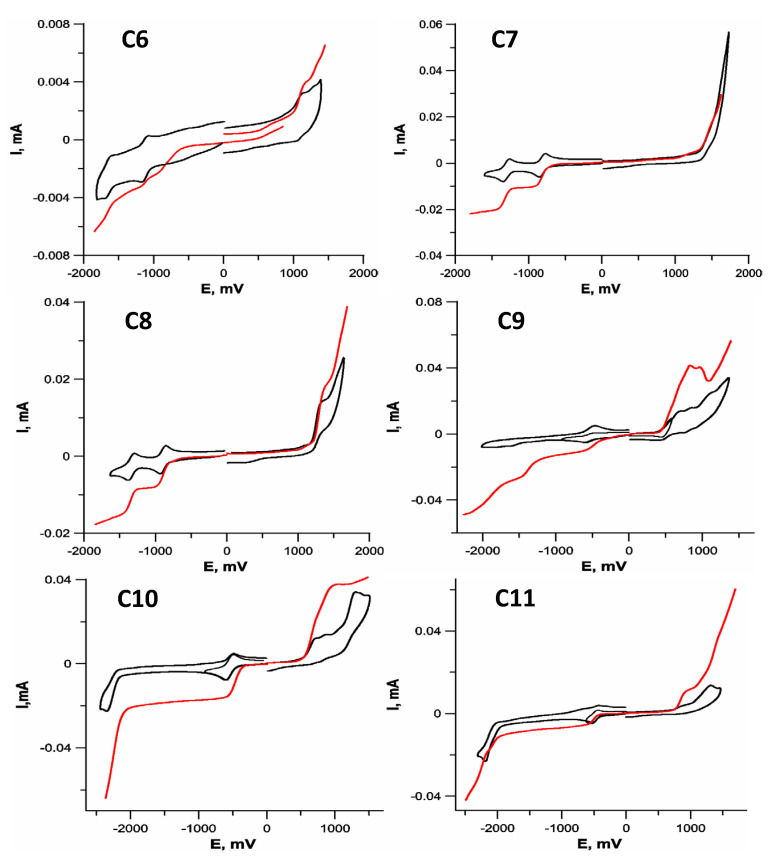
Cyclic voltammograms (black) and RDE curves (red) of **C6**–**C11**. GC electrode, DMF, 5.10–4 M, 0.1 M Bu_4_NClO_4_.

To evaluate the influence of the redox properties on the cytotoxicity upon Cu(II) reduction, we used CV and RDE techniques to measure the potentials of the Cu^2+^ → Cu^+1^ redox transition for **C1**–**C11** in DMF with 0.1 M Bu_4_NClO_4_ as a supporting electrolyte. CV determines the reduction potentials, while RDE allows for estimation of the oxidation state of copper [56] and E_1/2_ of the redox transition. The results are presented in Figure 2 and Table 1.

RDE voltammograms proved the Cu^+2^ oxidation state for all the initial complexes. According to their Cu^+2^ → Cu^+1^ reduction potentials, the tested complexes fell into three groups. The coordination compounds **C1**–**C5** (group 1, tetrahedral and planar square complexes with pyridyl-containing ligands and chloride anions) demonstrated a quasi-reversible Cu^+2^ → Cu^+1^ reduction at potentials E_Red_ ~0.33–0.42 V (Table 1). This redox transition took place in the region near NAC oxidation (~0.5 V [57,58,59]).

The coordination compounds of group 2 (complexes **C6**–**C8** with thiosemicarbazone ligands) had Cu^+2^ → Cu^+1^ redox transition potentials E_Red_ ~−0.52–−0.58 V (Figure 2 and Table 1). These values were intermediate relative to the complexes of groups 1 and 3 and are close to or slightly higher than the oxidation potential of NAC.

Finally, copper porphyrin complexes **C9**–**C11** (group 3) demonstrated Cu^+2^ → Cu^+1^ reduction at E_Red_ ~ −0.79–−1.08 V (Figure 2 and Table 1). Therefore, their reduction was much more difficult than in the group 1 complexes and took place at potentials ~1 V more cathodic than for **C1**–**C5**, significantly exceeding the oxidation potential of NAC.

### 2.3. Differential Effects of NAC on Cell Sensitization to Compounds ***C1***–***C11***

**Figure 3:**
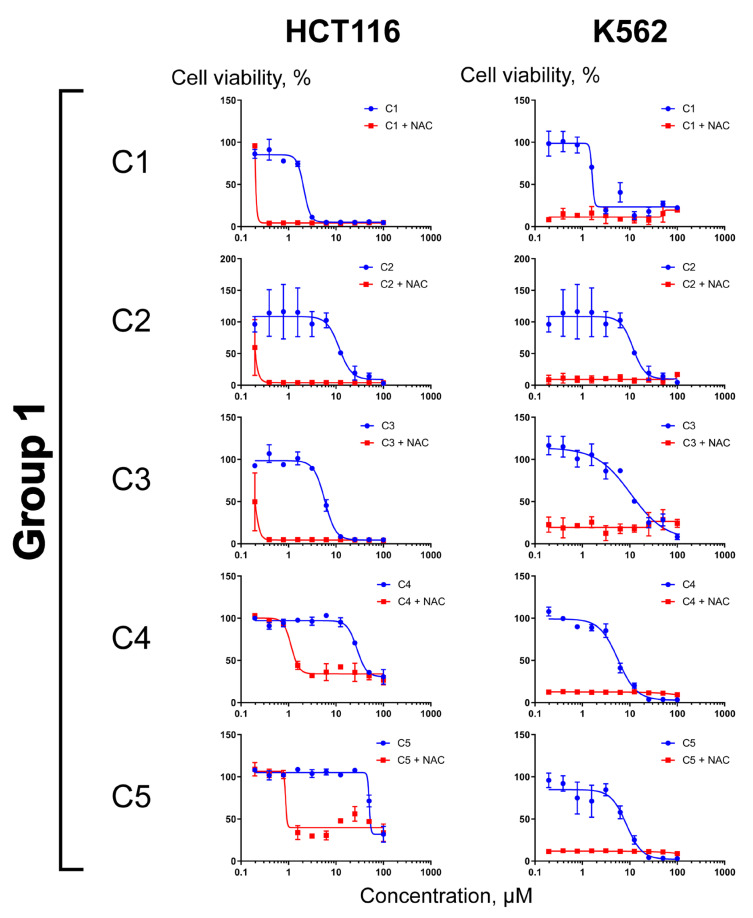

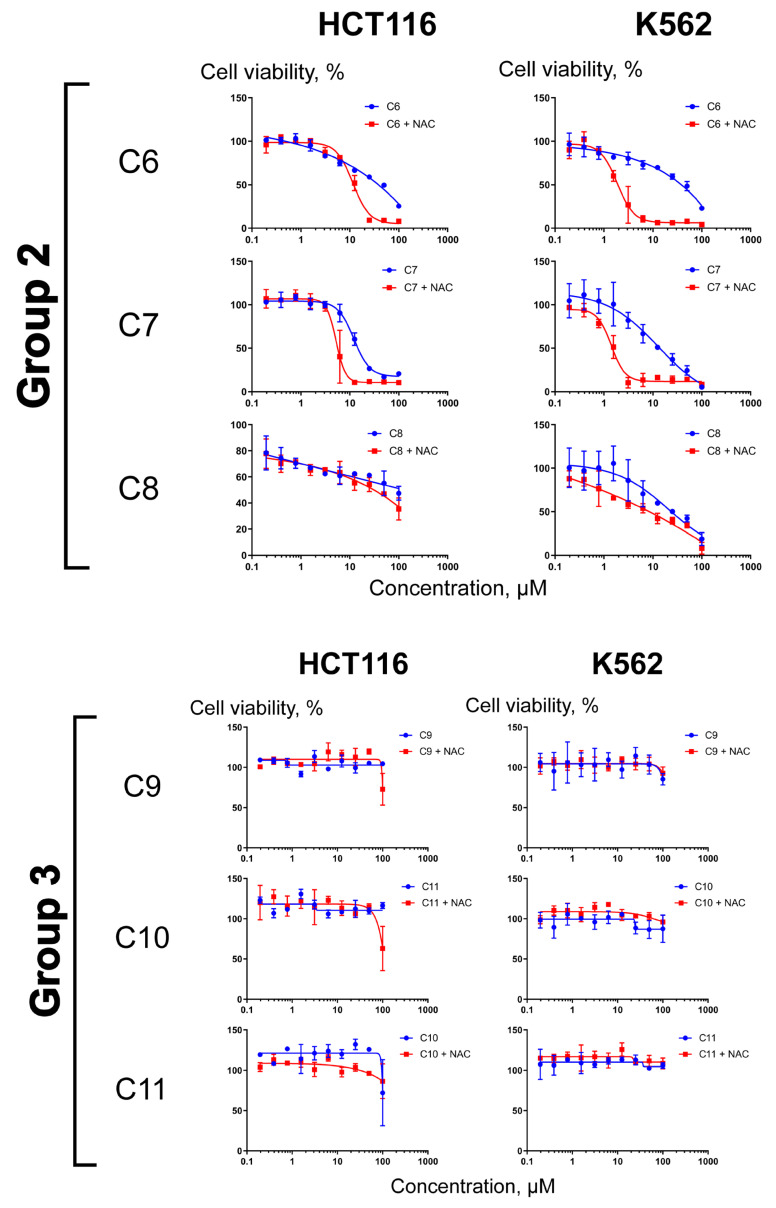
Differential effects of NAC on the cytotoxicity of the **C1**–**C11** copper–organic complexes.

Previously, we have demonstrated that the reduction of Cu^2+^ to Cu^1+^ by NAC or ascorbate resulted in rapid ROS generation and the death of cells of various tissue origins [4]. These effects were limited to CuO nanoparticles, as well as to some (but not all) Cu(II) organic complexes; neither other tested divalent metals nor copper-free compounds triggered ROS generation in combination with the reducing agents. Nevertheless, the potency of individual Cu(II)–organic compounds to kill cells in combination with the reducing agents differed significantly. One may expect that the electrochemical reduction potential is a key factor that determines the ability of copper-containing compounds to be reduced in the cellular context. To investigate whether the electrochemical parameters of copper reduction in cell-free systems (Figure 2 and Table 1) could be translated to the cytotoxicity, we compared the effects of NAC on the sensitization of HCT116 colon carcinoma and K562 leukemia cell lines to **C1**–**C11**. These models were chosen arbitrarily based on the cell line panel in [4]. Each compound was tested at 0.1–100 µM (twofold serial dilutions) in the absence or presence of 1 mM NAC. At this concentration, NAC alone had no effect on cell viability within 72 h (this study and Ref. [4]). As shown in Figure 3, NAC significantly sensitized both tested cell lines to the nontoxic concentrations of **C1**–**C5**. It is worth noting that accurate calculation of the IC_50_ values was difficult, because doubling of the drug concentration frequently led to an abrupt drop in survival. Such an explosive increase in cytotoxicity in the presence of NAC evidenced that the dependences of cell survival on the drug concentration were nonlinear. We therefore judged cell sensitization on the basis of the ratio IC_50_ (no NAC)/IC_50_ + NAC (Table S5). The sensitizing efficacy of (or fold sensitization by) NAC for **C1**–**C5** (group 1) was estimated as two to three orders of magnitude. The K562 cells were exceptionally sensitive to the combinations (Figure 3).

In striking contrast to the group 1 complexes, NAC was less potent in sensitizing the cells to the compounds of groups 2 and 3. The cytotoxicity of **C6**–**C8** in combination with NAC increased moderately. In HCT116 cells, the values of fold sensitization were ~4–5 for **C6** and **C7** (Figure 3). In K562 cells, these complexes were more active alone and together with NAC; the respective values of fold sensitization were within one order of magnitude. Compound **C8** was weakly potent in HCT116; the addition of NAC did not increase the cytotoxicity. In K562 cells, the cytotoxicity of this agent was somewhat bigger; still, the sensitizing effect of NAC was minor. Finally, in both cell lines, the copper–porphyrin complexes **C9**–**C11**, alone or with NAC, were virtually Inactive. Thus, cell-based experiments corroborated the results of the electrochemical measurements; the most efficient cell sensitization by NAC was achieved with complexes in which the reduction potential of Cu(II) was low.

The HCT116 (left column) and K562 (right column) cell lines were treated with **C1**–**C11** alone (blue curves) or in combination with 1 mM NAC (red curves) for 72 h, followed by MTT assays. Each value was the mean ± SD of three measurements.

Thus, the electronic properties of the ligand, as expected, evoked a dramatic effect on the ability of copper in the metal–organic complexes to be reduced. The low donor ability of uncharged 5-pyridylimidazolonone-thiohydantoin ligands **C1**–**C5** and the chloride anions with a strongly electronegative chlorine atom allow for Cu^+2^ → Cu^+1^ transition in the presence of NAC at potential values +0.35–0.55 V. Such an easy reduction of Cu^+2^ is accompanied by a robust ROS generation and cytotoxicity. Complexes **C6**–**C8** with more donor anionic thiosemicarbazone ligands that carry soft donor sulfur atoms are reduced already at negative potentials (~−0.5 V) and can therefore be converted, although only to a smaller extent, to Cu^1+^ complexes by NAC. Finally, porphyrin ligands containing harder donor nitrogen atoms most seriously impede the copper reduction in **C9**–**C11**; the redox transition occurred only at ~−1 V. Accordingly, NAC was unable to sensitize cells to these complexes. The influence of the electronic effects of ligands may be explained by changes in the HOMO energy of the metal–ligand-conjugated electron system. Measurements of the potential of Cu^+2^ → Cu^+1^ redox transition in copper–organic complexes can be used as an express method for predicting the cytotoxicity upon activation by the reducing agent.

To address the issue of the differential cytotoxicity of Cu(II) complexes with various scaffolds [4], we dissected the role of the organic environment geometry vs. the electrochemical reduction potential. In so doing, we compared the copper complexes with different spatial structures. Of note, in vivo, the copper-containing complexes most likely change the geometry of their coordination environment upon binding to serum albumin or other transport proteins. However, some arguments can favor the fact that the copper complexes discussed herein basically retain the geometry of their coordination environment in solutions. Thus, the UV–Vis spectra of coordination compounds **C1**–**C3** [31] are consistent with the geometry of the coordination environment established for the same complexes in the crystalline state. Similarly, for the complex **C5** described in [33], UV–Vis spectroscopy in DMF solutions confirmed the tetrahedral geometry of the coordination environment. For **C4** [32], the EPR study revealed that the spectra of all the complexes were similar and typical for mononuclear S = 1/2 Cu(II) complexes. The EPR spectra of **C4** in frozen solutions showed uniaxial spectra consistent with Cu(II) ions in square planar or axially elongated octahedral crystal fields, with the unpaired electron in the d_x_2-_y_2 orbital, indicating the approximate geometry of Cu(II) species generated from the **C4** complex. For copper–porphyrin complexes **C9**–**C11** [35], the UV–Visible absorption spectra in DMSO solutions are consistent with the data on many porphyrin complexes usually reported as compounds with a square planar (in some cases, somewhat distorted) environment of metal ions.

Importantly, this study demonstrated that the ability to kill cells with the combination of the Cu–organic complex and the reducing agent is determined precisely by the redox potential of the copper cation and does not explicitly depend on the geometry of the metal coordination environment and the nature of the ligand’s donor atoms. Thus, among the complexes of group 1 that demonstrated a significant activation by NAC, there are both tetrahedral (compounds **C1**–**C3** and **C5**) and square planar (**C4**) complexes. The donor fragments in this group included the pyridine/imidazolone combination (**C1**–**C3**), as well as pyridyl/pyridylbenzimidazole (**C4**) or pyridyl/pyridylbenzothiazole (**C5**). Indeed, the close values of Cu^+2^ → Cu^+1^ redox transitions rather than differences in the spatial structure of **C1**–**C5** determine their similar behavior in the presence of the reducing agent. Thus, in order to obtain Cu^2+^ coordination compounds with a pronounced ability to be activated by NAC or other reducing agents, it is preferable to use uncharged organic ligands that carry hard electronegative inorganic moieties.

Previously, we have analyzed the mechanism of cytotoxicity evoked by combinations of CuO nanoparticles or Cu(II)–organic compounds with physiological-reducing agents [4]. This complex mechanism is triggered by Cu^+2^ → Cu^+1^ redox transition and involves the perturbations of the plasma membrane leading to the loss of its integrity. We claim that this mechanism differs from an approach common for targeted therapy, namely, the inhibition of an intracellular moiety vital for tumor cells but tolerable for nonmalignant counterparts. In contrast, drug combinations that cause Cu^+2^ → Cu^+1^ redox transition are likely to be applicable when the conventional therapies are no longer efficient. In these situations, the oxidative damage of the plasma membrane emerges as a method of choice, since the necrotic mechanism remains functional in cells otherwise resistant to many chemotherapeutics [60,61]. Furthermore, the ROS-mediated elimination of tumor-associated stromal cells is therapeutically beneficial, because these species support tumor growth [62,63]. Our data suggest that ROS-induced salvation therapy upon Cu^2+^ reduction would be useful in an advanced disease.

## 3. Materials and Methods

### 3.1. General

All chemicals purchased from Merck (Darmstadt, Germany), Lancaster and ABCR were reagent grade and used without purification. Melting points were determined using an OptiMelt MPA100–Automated melting point system (Stanford Res. Syst., Sunnyvale, CA, USA), 1 °C/min, 0.1 °C resolution. NMR spectra were acquired on a Bruker (Billerica, MA, USA) Avance 400 at room temperature; the chemical shifts δ were referenced to the solvents (CDCl_3_: δH = 7.26 ppm; δC = 77.16 ppm; DMSO-*d*6: δH = 2.50 ppm; δC = 39.6 ppm). Infrared spectra were recorded on a Thermo (Waltham, MA, USA) Nicolet iS5 FTIR, the number of scans was 32, resolution 4 cm^−1^ and sampling ATR. High-resolution mass spectra were recorded on a G3 QTof (Waters, Milford, MA, USA) mass spectrometer. For solutions with a concentration of 0.1–9 µg mL^−1^ in 1% formic acid in acetonitrile, direct injection with a syringe pump into the ion source was used (5 µL min^−1^). Elemental analysis of CHNS/O was performed using a Perkin Elmer Model 2400 Series II (Perkin Elmer, Waltham, MA, USA).

### 3.2. X-ray Diffraction Analysis

Data were collected on a STOE (Darmstadt, Germany) diffractometer, a Pilatus (Stans, Switzerland) 100 K detector, the focusing mirror collimation of Cu Kα (1.54186 Å) radiation and the rotation method mode. The structures were solved and refined with the SHELX program [64]. Molecular graphics was prepared using MERCURY software 2020.3.0 [65]. Tables S1–S4 present lengths [E], angles [◦], torsion angles [◦] and hydrogen bonds. Figures S1 and S2 show the unit cell and thermal ellipsoid plots at a 50% probability level for compound **7** (see the Supplementary Materials).

### 3.3. MALDI

Mass spectra of matrix-activated laser desorption/ionization (MALDI) were recorded on a BrukerAutoflex II instrument (resolution FWHM 18000) equipped with a nitrogen laser with a working wavelength 337 nm and a time-of-flight mass analyzer operating in the reflectron mode (accelerating voltage 20 kV). Samples were applied on a polished steel substrate. Spectra were recorded in the positive ion mode. The resulting spectrum was the sum of 50 measurements at different points in the sample. *Trans*-2-[3-(4-*tert*-butylphenyl)-2-methyl-2-propenylidene] malononitrile (DCTB) (Acros, Organics, Thermo Fisher Sci., Waltham, MA, USA; 99%) was the preferred matrix to facilitate ionization.

### 3.4. Electrochemistry

An IPC Pro M potentiostat (Volta, Saint-Petersburg, Russia) was used for electrochemical studies. The working electrode was a glassy carbon disk (d = 2 mm), and the reference electrode was Ag/AgCl/KCl (sat.). The auxiliary electrode was a platinum plate, and the supporting electrolyte was 0.1 M Bu_4_NClO_4_ solution in DMF. Potential scan rates were 100 mV/s and 20 mV/s^−1^ in the cyclic voltammetry (CV) and voltammetry with a rotating disk electrode (RDE) methods, respectively. Measurements were carried out in a dry argon atmosphere; samples were dissolved in a de-aerated solvent.

### 3.5. Cell Culture and Cytotoxicity Assays

Human HCT116 colon carcinoma and K562 chronic myelogenous leukemia cells lines (American Type Culture Collection, Manassas, VA, USA) were cultured in Dulbecco’s modified Eagle’s medium or RPMI-1640, respectively, supplemented with 10% fetal bovine serum (HyClone, Logan, UT, USA) and 50 μg/mL gentamicin at 37 °C, 5% CO_2_ in a humidified atmosphere. Copper–organic complexes **C1**–**C11** were dissolved in dimethyl sulfoxide as 10 mM stock solutions. Aqueous dilutions were prepared on the day of the experiments. NAC was dissolved in culture media as 50 mM stock solution, then pH was adjusted to 7.2–7.4 with 1 M NaOH. The cytotoxicity of **C1**–**C11** alone or in combination with NAC was determined with MTT assays [4].

### 3.6. Synthesis

The synthetic procedures are presented in the Supplementary Materials.

## 4. Conclusions

Our results demonstrated that, for triggering the cytotoxicity of copper (II)–organic complexes by NAC, the reduction potential in the complex must be lower than the oxidation potential of the reducing agent. This difference gives rise to an explosive ROS generation and cytotoxicity. At the same time, the geometry of the copper coordination environment, i.e., tetrahedral or square planar, showed no decisive effect on the ability of the complex to be reduced; therefore, no cell sensitization was observed. Based on this, the measurement of the potential of Cu^+2^ → Cu^+1^ redox transition in copper complexes with the organic ligand can be used as an express method for predicting whether their cytotoxicity will be triggered by NAC or another reducing agent.

## Figures and Tables

**Table 1:** Electrochemical reduction potentials E_Red_, E_1/2_ of copper–organic complexes **C1**–**C11**.

	Comp.	E_Red_, V	E_1/2_, V
**Group 1**	C1	0.33/0.54	ND
	C2	0.42/0.55	0.49
	C3	0.37/0.55	0.43
	C4	ND	0.33
	C5	0.34/0.52	ND
**Group 2**	C6	−0.58/−0.48	−0.49
	C7	−0.57/−0.50	−0.53
	C8	−0.52/−0.46	−0.50
**Group 3**	C9	−1.08/−1.16	−1.12
	C10	−0.79/−0.85	−0.82
	C11	−1.05/−0.97	−1.02

ND, not determined.

## Data Availability

Data are contained within the article and Supplementary Materials.

## Supplementary Materials

The following supporting information can be downloaded at https://www.mdpi.com/article/10.3390/molecules29051032/s1. Figure S1: The unit cell for (**7**). Figure S2: Crystal structure of (**7**), thermal ellipsoids are at 50% level. Figure S3: UV-Vis spectra of complexes **C6**–**C8** (5 × 10^−5^ M) in DMF solution. Table S1: Crystal data and structure refinement for compound **7**. Table S2: Hydrogen bonds for (**7**) [Å and °]. Table S3: Bond lengths [Å] and angles [°] for (**7**). Table S4: Torsion angles [°] for (**7**). Table S5: Calculated IC_50_ values (µM) for copper-organic complexes.
