# Supplementary material for: The Copper Reduction Potential Determines the Reductive Cytotoxicity: Relevance to the Design of Metal–Organic Antitumor Drugs
Source: Molecules. 2024 Feb 27;29(5):1032. doi: 10.3390/molecules29051032 (PMC10934576; doi:10.3390/molecules29051032)
Supplement: Supplementary file 1 [file molecules-29-01032-s001.zip › molecules-2824986-supplementary.pdf]

Supplementary Materials

The Copper Reduction Potential Determines the Reductive Cytotoxicity: Relevance to the Design of Metal–Organic Antitumor Drugs

Elena K. Beloglazkina, Anna A. Moiseeva, Sergey A. Tsymbal, Dmitry A. Guk, Mikhail A. Kuzmin, Olga O. Krasnovskaya, Roman S. Borisov, Elena S. Barskaya, Victor A. Tafeenko,Victoria M. Alpatova, Andrei V. Zaitsev, Alexander V. Finko, Valentina A. Ol'shevskaya, Alexander A. Shtil*

*Corresponding author. E-mail: shtilaa@yahoo.com

Table of Content

**Contents**	**Page**
Synthesis	S2
NMR, FTIR and HRMS spectra of **L6–L8**	S5
FTIR and MALDI spectra of **C6–C8**	S43
X-ray data (Tables S1–S4 and Figures S1 and S2)	S49
Figure S3. UV–Vis spectra of complexes **C6 – C8**	S52
Table S5. Calculated IC_{50} values (μM) for copper–organic complexes	S53

Synthesis

Copper complexes of 5-pyridylmethyleneimidazolones **C1** – **C3** (Figure 1) were obtained according to the procedure described in [31]. The pyridylbenzimidazole complex **C4** was synthesized according to [32] and the pyridylbenzothiazole complex **C5** according to [33]. To obtain ligands **L6–L8** and complexes **C6–C8**, the transformations depicted in Scheme 1 were performed. Ligands **L6–L8** were designed according to [34]. Details of the chemical synthesis are given below. The copper–porphyrin complexes **C9–C11** were synthesized as reported in [35].

N-phenethylhydrazinecarbothioamide (**1**) was synthesized as described [36]. In a 50 mL round-bottom flask, 2-phenylethylisothiocyanate (1.28 g, 7.85 mmol) was dissolved, then 98% hydrazine hydrate (785 µl, 16.17 mmol) was added dropwise and stirred at room temperature for 2 h. The solvent was evaporated in vacuo. The residue was recrystallized from 70% aqueous methanol. Yield 1.19 g (78%). White solid, mp = 115 – 116 °C. ^{1}H NMR (400 MHz, $CDCl_3$) δ 2.92 (t, J = 7.1 Hz, 2H, CH_2Ph), 3.73 (br.s, 2H, NH_2), 3.86 (q, J = 7.1 Hz, 2H, CH_2NH), 7.18–7.25 (m, 3H, H_{Ar}), 7.27–7.34 (m, 2H, H_{Ar}), 7.48 (t, J = 5.8 Hz, 1H, $NHNH_2$), 8.04 (br. s, 1H, CH_2NH).

2-(3-oxobutan-2-ylidene)-N-phenethylhydrazine-1-carbothioamide (**2**). In a round-bottom flask equipped with a dropping funnel and a thermometer, diacetyl (1.14 mL, 13 mmol) was dissolved in dry THF, and catalytic amounts of concentrated HCl were added. A solution of **1** (1.14 g, 4.35 mmol) in 5 mL dry THF was slowly added to this solution at 0 °C. The reaction was carried out with constant stirring for 2 h; TLC control, eluent EtOAc:petroleum ether (1:4). At the end of the reaction, the solvent was evaporated in vacuo. The product was isolated by column chromatography on SiO_2, and the eluent was EtOAc:petroleum ether (1:4). Yield 0.962 g (84%). Pale yellow solid, mp = 78 – 79 °C. ^{1}H NMR (400 MHz, $CDCl_3$): δ 1.96 (s, 3H, CH_3CN), 2.19 (s, 3H, CH_3CO), 3.00 (t, J = 6.7 Hz, 2H, CH_2Ph), 4.01 (q, J = 6.6 Hz, 2H, CH_2NH), 7.21 – 7.36 (m, 5H, H_{Ar}), 7.53 (s, 1H, NH), 8.72 (s, 1H, NH). ^{13}C NMR (400 MHz, $CDCl_3$) δ 8.9, 24.4, 34.8, 45.6, 127.0, 128.8 (2C), 129.0 (2C), 138.2, 144.9, 177.7, 196.4. FTIR (Diamond, ν/cm^{-1}): 701, 753, 996, 1130, 1175, 1209, 1232, 1298, 1360, 1428, 1454, 1503, 1538, 1608, 1688, 2937, 3026, 3174, 3337. HRMS $C_{13}H_{17}N_3OS$ (ESI TOF+MS) m/z: 264.1164 (Calc. for 264.1165 $[M+H]^+$).

N-methyl-N-phenylhydrazinecarbothioamide (**3**) was synthesized as described [37]. A solution (1.77 g, 7.33 mmol) of 2-((methyl(phenyl)carbamothioyl)thio)acetic acid was treated with 98% hydrazine hydrate (2 mL) and water (1 mL) and then heated in a water bath at 85 °C for 30 min. The precipitate was separated by filtration, washed with water, dried and then recrystallized from a mixture of 5 mL EtOH and 5 mL water. Yield 1.06 g (81%). White solid, mp = 123–124 °C. ^{1}H NMR (400 MHz, DMSO-*d6*) δ 3.42 (s, 3H, CH_3N), 4.80 (br.s, 2H, $NHNH_2$), 7.10–7.49 (m, 5H, H_{Ar}), 8.60 (br. s, 1H, $NHNH_2$).

(E)-N-methyl-2-((E)-3-(2-(phenethylcarbamothioyl)hydrazineylidene)butan-2-ylidene)-N-phenylhydrazine-1-carbothioamide (**4**). In a round-bottom flask, a mixture of **2** (1.46 g, 5.6 mmol) and **3** (1 g, 5.6 mmol) was refluxed under argon in dry methanol 20 mL in the presence of catalytic amounts of acetic acid. The reaction was carried out for 6 h until a precipitate formed. The reaction mixture was cooled to room temperature, and the precipitate was filtered off and washed with a small amount of methanol. Yield 1.4 g (60%). White solid, mp = 193–194 °C. ^{1}H NMR (400 MHz, DMSO-*d6*): δ 2.17 (s, 3H, CH_3C), 2.19 (s, 3H, CH_3C), 2.91 (t, J = 7.6 Hz, 2H, CH_2Ph), 3.56 (s, 3H, CH_3N), 3.73–3.86 (m, 2H, CH_2NH), 7.14–7.56 (m, 10H, H_{Ar}), 8.40 (t, J = 5.9 Hz, 1H, CH_2NH), 9.32 (br. s, 1H, NH), 10.35 (br. s, 1H, NH). ^{13}C NMR (100 MHz, DMSO-*d6*): δ 11.6, 11.9, 34.5, 43.0, 45.2, 126.2, 126.7, 127.9, 128.5 (2C), 128.6, 129.9, 129.9, 139.1, 143.0, 147.4, 149.3, 175.5, 177.7, 179.7, 206.6. FTIR (Diamond, ν/cm^{-1}): 698, 1065, 1133, 1208, 1267, 1366, 1409, 1471, 1490, 1529, 1595, 2946, 3218, 3338.

(E)-4-(4-aminostyryl)-N,N-dimethylaniline (**5**) was synthesized as described [38]. (E)-*N,N*-dimethyl-4-(4-nitrostyryl)aniline (2.27 g, 8.48 mmol), $SnCl_2$ (9.5 g, 50.01 mmol) and 50 ml of ethanol were mixed in a flask. The reaction mixture was refluxed under argon for 5 h, the solvent was evaporated in vacuo and the residue was resuspended in water. The solution was adjusted to pH 10–11, and the product was extracted with diethyl ether (2 × 100 mL). The solvent was evaporated. The product was used without purification. Yield 1.7 g (84%). Yellow solid, mp = 148–149 °C. ^{1}H NMR (400 MHz, DMSO-*d6*) δ 2.89 (s, 6H, Me×2), 5.18 (s, 2H, NH_2), 6.54 (d, J = 8.5 Hz, 2H, H_{Ar}), 6.68 (d, J = 8.9 Hz, 2H, H_{Ar}), 6.78 (s, 2H, CH×2), 7.20 (d, J = 8.5 Hz, 2H, H_{Ar}), 7.32 (d, J = 8.9 Hz, 2H, H_{Ar}).

(E)-N-(4-((E)-4-(dimethylamino)styryl)phenyl)-2-((E)-3-(2-(phenethylcarbamothioyl)hydrazineylidene)butan-2-ylidene)hydrazine-1-carbothioamide (**L6**). In a round-bottom flask, a refluxed mixture of **4** (30 mg, 73 mmol) and **5** (20 mg, 84 mmol) was boiled under argon for 20 h in 10 ml of dry acetonitrile in the presence of catalytic amounts of acetic acid. The mixture was cooled to room temperature, and the precipitate was filtered off and washed with acetonitrile. Yield 27 mg (60%). Orange solid, mp = 220–221 °C. ^{1}H NMR (400 MHz, DMSO-*d6*) δ 2.22 (s, 3H, CH_3CNNH), 2.25 (s, 3H, CH_3CNNH), 2.88–2.92 (m, 8H, CH_3N×2 and CH_2Ph), 3.78 (q, J =

6.7 Hz, 2H, CH2NH), 6.71 (d, J = 8.5 Hz, 2H, H_{Ar}), 6.96 (d, J = 16.4 Hz, 1H, CHPh), 7.11 (d, J = 16.3 Hz, 1H, CHPh), 7.20–7.33 (m, 5H, H_{Ar}), 7.42 (d, J = 8.4 Hz, 2H, H_{Ar}), 7.43–7.57 (m, 4H, H_{Ar}), 8.42 (t, J = 5.7 Hz, 1H, CH_2NH), 9.95 (s, 1H, NH), 10.39 (s, 1H, NH), 10.60 (s, 1H, NH). ^{13}C NMR (100 MHz, DMSO-*d6*) δ 11.8, 12.0, 34.5, 40.0 (2C), 45.2, 112.2 (2C), 123.0, 125.0, 125.4 (2C), 125.4 (2C), 126.2, 127.4 (2C), 128.4, 128.5 (2C), 128.6 (2C), 135.0, 137.4, 139.1, 147.9, 149.0, 149.9, 176.4, 177.7. FTIR (Diamond, ν/cm^{-1}): 825, 967, 1078, 1135, 1185, 1253, 1343, 1419, 1490, 1525, 1582, 1607, 2792, 2847, 2881, 2935, 3021, 3222, 3295, 3368. HRMS $C_{30}H_{35}N_7S_2$ (ESI TOF+MS) m/z: 558.2462 (Calc. for 558.2468 [M+H]$^+$).

N-allylhydrazinecarbothioamide (6) was synthesized as described [39]. Allyl isothiocyanate (2 g, 20.17 mmol) was dissolved in a 50 mL round-bottom flask, then hydrazine hydrate (2.01 mL, 20.17 mmol) was added dropwise. The mixture was stirred at room temperature for 2 h, and then, the solvent was evaporated in vacuo. The residue was recrystallized from 70% aqueous methanol. Yield 1.9 g (75%). White solid, mp = 96 °C. ^{1}H NMR (400 MHz, $CDCl_3$) δ 3.81 (br.s, 2H, NH_2), 4.27 (t, J = 5.7 Hz, 2H, CH_2NH), 5.09–5.27 (m, 2H, CH_2CH), 5.81–6.00 (m, 1H, CH_2CH), 7.52 (s, 1H, NH), 8.07 (br.s, 1H, NH). ^{13}C NMR (101 MHz, $CDCl_3$) δ 46.41, 116.77, 133.84, 182.14.

(E)-N-allyl-2-(3-oxobutan-2-ylidene)hydrazine-1-carbothioamide (**7**). In a 50 ml round-bottom flask equipped with an addition funnel and thermometer, diacetyl (3.81 mL, 43.44 mmol) was dissolved in dry THF, then catalytic amounts of concentrated HCl were added. A solution of **6** (1.9 g, 14.48 mmol) in dry THF was slowly added to this solution at 0 °C. The reaction was carried out with stirring for 2 h. TLC control, eluent EtOAc:petroleum ether (1:4). After the completion of the reaction, the solvent was evaporated in vacuo. The product was isolated by column chromatography on SiO_2, eluent EtOAc:petroleum ether (1:4). Yield 2.3 g (80%). Yellow solid, mp = 107–108 °C. ^{1}H NMR (400 MHz, $CDCl_3$) δ 2.01 (s, 3H, MeCN), 2.41 (s, 3H, MeCO), 4.38 (t, J = 5.6 Hz, 2H, CH_2NH), 5.23–5.31 (m, 2H, CH_2CH), 5.92–6.01 (m, 1H, CH_2CH), 7.58 (s, 1H, NH), 8.71 (s, 1H, NH). ^{13}C NMR (100 MHz, $CDCl_3$): δ 9.2, 24.6, 47.0, 117.5, 132.7, 145.3, 178.3, 196.4. (Diamond, ν/cm^{-1}): 621, 682, 834, 899, 912, 961, 1010, 1042, 1105, 1141, 1178, 1274, 1364, 1419, 1434, 1505, 1541, 1588, 1648, 1676, 2909, 2987, 3009, 3090, 3220, 3341. HRMS $C_8H_{13}N_3OS$ (ESI TOF+MS) m/z: 200.0852 (Calc. for 200.0852 [M+H]$^+$).

(E)-2-((E)-3-(2-(allylcarbamothioyl)hydrazineylidene)butan-2-ylidene)-N-methyl-N-phenylhydrazine-1-carbothioamide (**8**). In a round-bottom flask, a mixture of **3** (1.134 g, 6.26 mmol) and **7** (1.25 g, 6.26 mmol) was refluxed under argon in dry methanol 20 mL in the presence of catalytic amounts of acetic acid. The reaction was carried out for 6 h until the formation of a precipitate and cooled to room temperature. The precipitate was filtered off and washed with a small amount of methanol. Yield 1.8 g (79%). White solid, mp = 199–200 °C. ^{1}H NMR (400 MHz, DMSO-*d6*) δ 2.19 (s, 3H, MeCN), 2.22 (s, 3H, MeCN), 3.56 (s, 3H, MeN), 4.23 (t, J = 5.7 Hz, 2H, CH_2NH), 5.08–5.16 (m, 2H, CH_2CH), 5.86–5.95 (m, 1H, CH_2CH), 7.33–7.57 (m, 5H, H_{Ar}), 8.56 (t, J = 6.0 Hz, 1H, CH_2NH), 9.32 (s, 1H, NH), 10.35 (s, 1H, NH). ^{13}C NMR (100 MHz, DMSO-*d6*) δ 11.6, 12.0, 43.0, 46.0, 115.6, 126.7, 127.9, 130.0, 134.8, 143.1, 147.6, 149.5, 175.6, 176.9, 178.1, 179.7. FTIR (Diamond, ν/cm^{-1}): 619, 669, 696, 742, 770, 808, 851, 874, 908, 932, 954, 1055, 1106, 1130, 1207, 1238, 1264, 1290, 1306, 1359, 1414, 1462, 1529, 1585, 1594, 1649, 2912, 2977, 3184, 3346.

(E)-N-allyl-2-((E)-3-(2-((4-((E)-4-(dimethylamino)styryl)phenyl)carbamothioyl)hydrazineylidene)butan-2-ylidene)hydrazine-1-carbothioamide (**L7**). In a round-bottom flask, a mixture of **8** (1.21 g, 3.32 mmol) and **5** (0.792 g, 3.32 mmol) was refluxed under argon in dry acetonitrile (30 mL) in the presence of catalytic amounts of acetic acid. The reaction was carried out for 20 h and cooled to room temperature. The precipitate was filtered off and washed with acetonitrile. Yield 1.06 g (65%). Orange solid, mp = 221–222 °C. ^{1}H NMR (400 MHz, DMSO-*d6*) δ 2.27 (s, 3H, MeCN), 2.29 (s, 3H, MeCN), 2.93 (s, 6H, MeN×2), 4.24 (s, 2H, CH_2NH), 5.13 (dd, J = 13.5, 19.6 Hz, 2H, CH_2CH), 5.87–5.97 (m, 1H, CH_2CH), 6.72 (d, J = 8.4 Hz, 2H, H_{Ar}), 6.97 (d, J = 16.4 Hz, 1H, CHPh), 7.11 (d, J = 16.4 Hz, 1H, CHPh), 7.43 (d, J = 8.4 Hz, 2H, H_{Ar}), 7.49–7.76 (m, 4H, H_{Ar}), 8.58 (t, 1H, CH_2NH), 9.96 (s, 1H, NH), 10.37 (s, 1H, NH), 10.60 (s, 1H, NH). ^{13}C NMR (100 MHz, DMSO-*d6*) δ 11.8, 12.1 40.0 (2C), 46.0, 112.2 (2C), 115.6, 123.0, 125.0, 125.3 (2C), 125.4 (2C), 127.5 (2C), 128.4, 134.8, 135.1, 137.4, 148.1, 149.2, 149.9, 176.5, 178.0. FTIR (Diamond, ν/cm^{-1}): 625, 644, 678, 707, 721, 742, 783, 802, 822, 862, 928, 950, 966, 1012, 1059, 1130, 1166, 1191, 1201, 1249, 1321, 1351, 1412, 1485, 1518, 1580, 1605, 2789, 2839, 2879, 2974, 3017, 3194, 3289, 3364. HRMS $C_{25}H_{31}N_7S_2$ (ESI TOF+MS) m/z: 494.2158 (Calc. for 494.2155 [M+H]$^+$).

(E)-N-allyl-2-((E)-3-hydrazineylidenebutan-2-ylidene)hydrazine-1-carbothioamide (**9**). In a round-bottom flask, **7** (573 mg, 2.88 mmol) was dissolved in 10 mL of dry methanol. The reaction mixture was cooled to 0 °C, catalytic amounts of acetic acid were added and a solution of hydrazine hydrate (280 μl, 5.75 mmol) in 5 mL of dry methanol was added dropwise. The product was isolated by column chromatography on SiO_2, eluent EtOAc:petroleum ether (1:1). Yield 515 mg (84%). White

solid, mp = 147–148 °C. ^{1}H NMR (400 MHz, $CDCl_3$) δ 1.94 (s, 3H, Me), 2.07 (s, 3H, Me), 4.36 (t, J = 5.7 Hz, 2H, $\underline{CH_2}$NH), 5.23 (dd, J = 24.2, 14.4 Hz, 2H, $\underline{CH_2}$CH), 5.67 (s, 2H, NH_2), 5.91–6.00 (m, 1H, $CH_2\underline{CH}$), 7.50 (s, 1H, NH), 8.61 (s, 1H, NH). ^{13}C NMR (100 MHz, $CDCl_3$) δ 8.7, 10.3, 46.9, 116.9, 133.5, 145.1, 148.5, 178.1. FTIR (Diamond, ν/cm^{-1}): 907, 918, 942, 985, 1082, 1125, 1172, 1212, 1242, 1290, 1317, 1369, 1387, 1417, 1425, 1505, 1538, 1568, 1629, 2918, 2954, 2986, 3081, 3210, 3284, 3366 HRMS $C_8H_{15}N_5S$ (ESI TOF+MS) m/z: 214.1118 (Calc. for 214.1121 $[M+H]^+$).

2-(4-isothiocyanatophenyl)benzo[d]thiazole (**10**). In a three-necked flask, 4-(benzothiazol-2-yl)aniline (50 mg, 0.22 mmol) was dissolved in 1 mL of DCM, then 1 mL of water was added. Simultaneously, 1.5 mL of a $NaHCO_3$ solution (55.7 mg, 0.66 mmol) and 1 mL of a solution of thiophosgene (50.81 mg, 0.44 mmol) in 1 mL of DCM were added with vigorous stirring for 1 h at room temperature. Then, the organic phase was separated, and the solvent was evaporated. The product was isolated by column chromatography on SiO_2, eluent EtOAc:petroleum ether (1:10). Yield 57 mg (96%). White solid, mp = 154–155 °C. ^{1}H NMR (400 MHz, $CDCl_3$) δ 7.30 (d, J = 8.6 Hz, 2H, H_{Ar}), 7.40 (t, J = 7.6 Hz, 1H, H_{Ar}), 7.50 (t, J = 8.3 Hz, 1H, H_{Ar}), 7.89 (d, J = 7.7 Hz, 1H, H_{Ar}), 8.00–8.11 (m, 3H, H_{Ar}). ^{13}C NMR (100 MHz, $CDCl_3$) δ 121.8, 123.6, 125.7, 126.46, 126.49, 126.7, 128.8 (2C), 132.6, 133.7, 135.3, 137.4, 154.3, 166.3. FTIR (Diamond, ν/cm^{-1}): 550, 594, 617, 631, 693, 717, 727, 754, 811, 837, 865, 924, 965, 1016, 1071, 1106, 1128, 1171, 1226, 1240, 1251, 1289, 1315, 1412, 1435, 1456, 1479, 1517, 1557, 1573, 1600, 1651, 1667, 1682, 1699, 1732, 1799, 2044, 2105, 2178, 3053. HRMS $C_{14}H_8N_2S_2$ (ESI TOF+MS) m/z: 269.0191 (Calc. for 269.0202 $[M+H]^+$).

(E)-N-allyl-2-((E)-3-(2-((4-(benzo[d]thiazol-2-yl)phenyl)carbamothioyl)hydrazineylidene)butan-2-ylidene)hydrazine-1-carbothioamide (**L8**). In a round-bottom flask, **9** (50 mg, 0.234 mmol) and **10** (62 mg, 0.234 mmol) flasks was refluxed in dry methanol (5 ml) for 24 h. The precipitate was filtered off and washed with methanol. Yield 46 mg (40%). Yellow solid, mp = 221–222 °C. ^{1}H NMR (400 MHz, DMSO-*d*$_6$) δ 2.29 (s, 3H, Me), 2.31 (s, 3H, Me), 4.25 – 4.26 (m, 2H, $\underline{CH_2}$NH), 5.13 (dd, J = 19.2, 14.1 Hz, 2H, $\underline{CH_2}$CH), 5.88 – 5.96 (m, 1H, $CH_2\underline{CH}$), 7.48–7.53 (m, 2H, H_{Ar}), 7.89 (d, *J* = 8.2 Hz, 2H, H_{Ar}), 8.05–8.16 (m, 4H, H_{Ar}), 8.60 (t, *J* = 6.1 Hz, 1H, $CH_2\underline{NH}$), 10.16 (s, 1H, NH), 10.42 (s, 1H, NH), 10.82 (s, 1H, NH). ^{13}C NMR (100 MHz, DMSO-*d*$_6$) δ 11.9, 12.2, 46.0, 115.6, 122.4, 122.8, 125.3 (2C), 125.4, 126.7, 127.1 (2C), 129.4, 134.5, 134.8, 141.8, 148.0, 149.8, 153.7, 166.8, 176.4, 178.1. FTIR (Diamond, ν/cm^{-1}): 758, 822, 836, 965, 1074, 1133, 1177, 1201, 1253, 1317, 1356, 1428, 1491, 1529, 1583, 1606, 2915, 2981, 3025, 3203, 3306, 3362 HRMS $C_{22}H_{23}N_7S_3$ (ESI TOF+MS) m/z: 482.1246 (Calc. for 482.1249 $[M+H]^+$).

General procedure for the preparation of coordination compounds **C6–C8**. Ligands **L6–L8** (1 eq.), $Cu(OAc)_2{\times}H_2O$ (1.1 eq.) and dry DMF (1–2 mL) were loaded into a round-bottom flask. The reaction mixture was stirred at room temperature. After 2 h, 1–2 mL of distilled water was added. The precipitate was separated by centrifugation, washed with water and dried on air or under reduced pressure.

*Compound **C6***. Yield 4.2 mg (74 %). Brown solid, mp = 250–251 °C. FTIR (Diamond, ν/cm^{-1}): 607, 536, 698, 748, 822, 858, 960, 1090, 1163, 1216, 1316, 1353, 1402, 1440, 1518, 1607, 1658, 2917, 3275, 3380. UV–Vis (λ, nm, (ε, l·mol^{-1}·cm^{-1})): 365 (46800), 485 (23000). MALDI-MS $C_{30}H_{33}CuN_7S_2$ m/z: 619.43 (Calc. for 619.31 $[M]^+$). Elemental analysis calc. for $C_{30}H_{33}CuN_7S_2$: C, 58.18; H, 5.37; Cu, 10.26; N, 15.83; S, 10.35; found C, 58.43; H, 5.20; N, 15.95; S, 10.31.

*Compound **C7***. Yield 4 mg (72 %). Reddish-brown solid, mp = 238–239 °C. FTIR (Diamond, ν/cm^{-1}): 560, 538, 615, 684, 734, 826, 934, 964, 993, 1093, 1178, 1217, 1248, 1320, 1361, 1404, 1462, 1506, 1557, 1520, 1585, 1625, 1607, 1661, 2916, 3015, 3162, 3254. UV–Vis (λ, nm, (ε, l·mol^{-1}·cm^{-1})): 369 (39400), 485 (19200). MALDI-MS $C_{25}H_{29}CuN_7S_2$ m/z: 555.45 (Calc. for 555.22 $[M]^+$). Elemental analysis calc. for $C_{25}H_{29}CuN_7S_2$: C, 54.08; H, 5.26; Cu, 11.45; N, 17.66; S, 11.55; found C, 54.37; H, 5.32; N, 17.51; S, 11.49.

*Compound **C8***. Yield 3.9 mg (70 %). Reddish-brown solid, mp = 270–271 °C. FTIR (Diamond, ν/cm^{-1}): 549, 611, 623, 649, 667, 700, 725, 753, 822, 852, 839, 930, 968, 995, 1086, 1163, 1176, 1189, 1212, 1255, 1316, 1402, 1417, 1428, 1455, 1471, 1543, 1489, 1599, 2980, 3221, 3419. UV–Vis (λ, nm, (ε, l·mol^{-1}·cm^{-1})): 384 (49800), 485 (21200). MALDI-MS $C_{22}H_{21}CuN_7S_3$ m/z: 543.25 (Calc. for 543.19 $[M]^+$). Elemental analysis calc. for $C_{22}H_{21}CuN_7S_3$: C, 48.65; H, 3.90; Cu, 11.70; N, 18.05; S, 17.71; found C, 48.57; H, 4.22; N, 18.10; S, 17.79.

NMR, FTIR and HRMS Spectra

NMR ^{1}H *N-phenethylhydrazinecarbothioamide* (**1**).

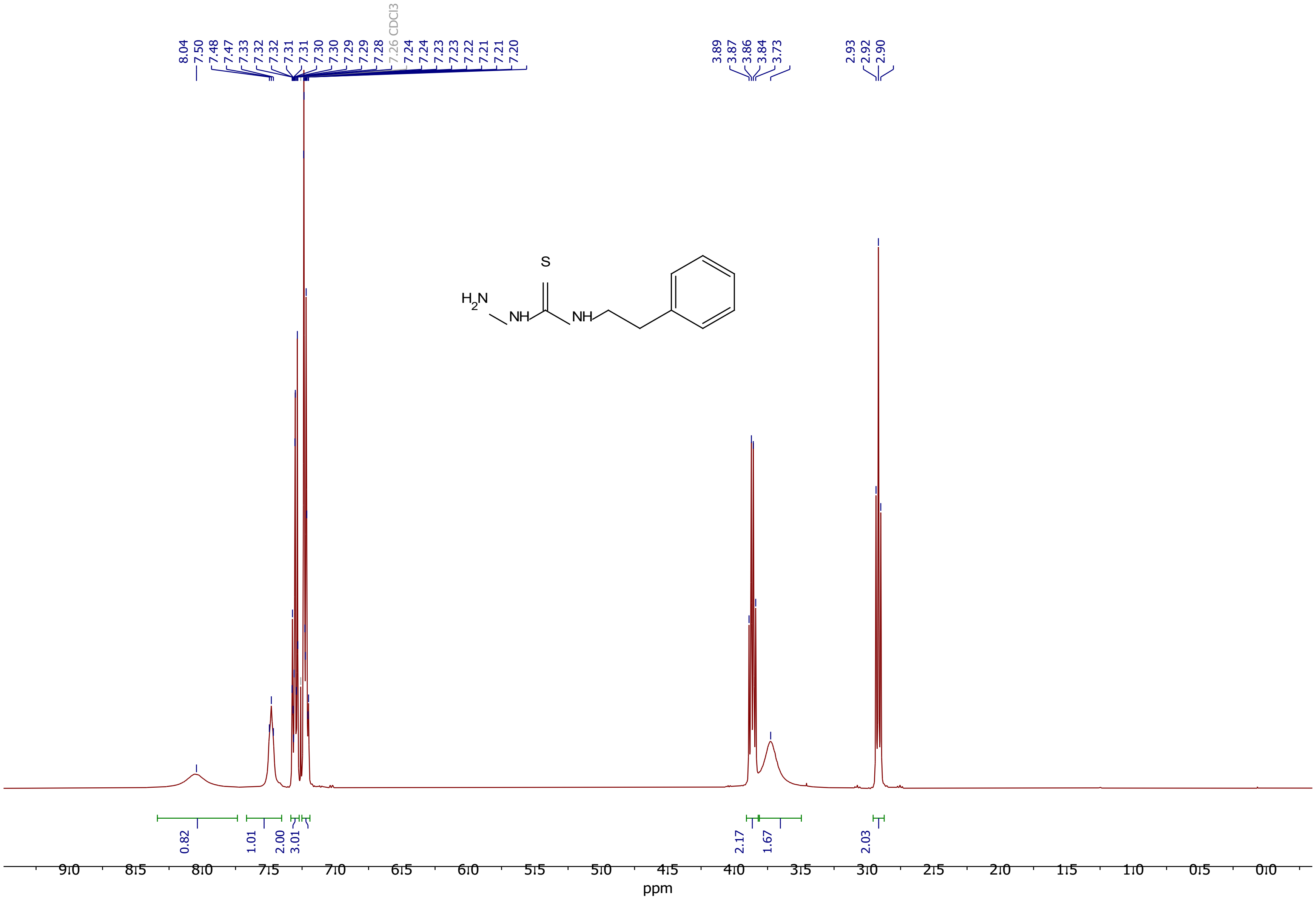

NMR ^{1}H *2-(3-oxobutan-2-ylidene)-N-phenethylhydrazine-1-carbothioamide* (**2**).

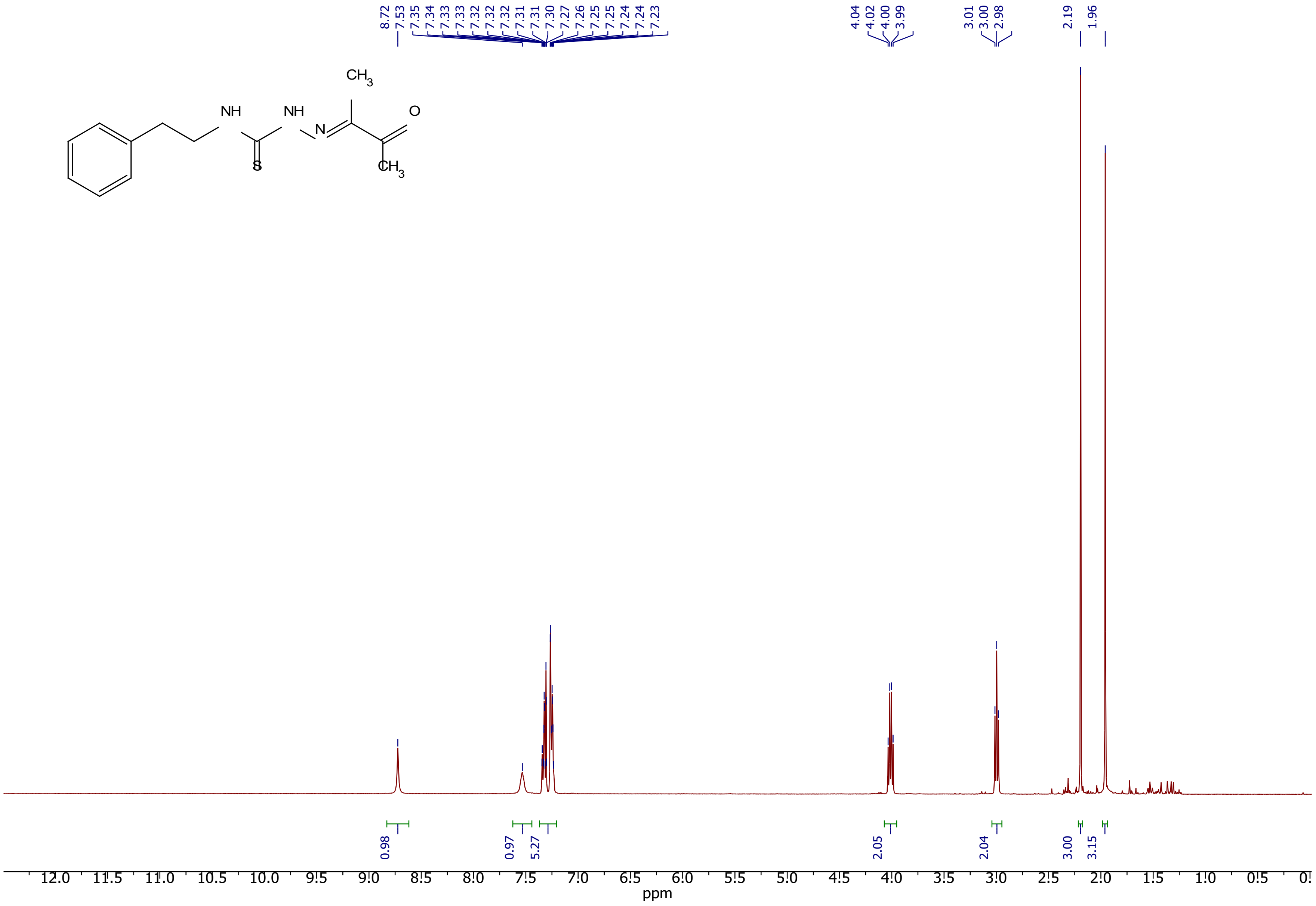

NMR ^{13}C *2-(3-oxobutan-2-ylidene)-N-phenethylhydrazine-1-carbothioamide* (**2**).

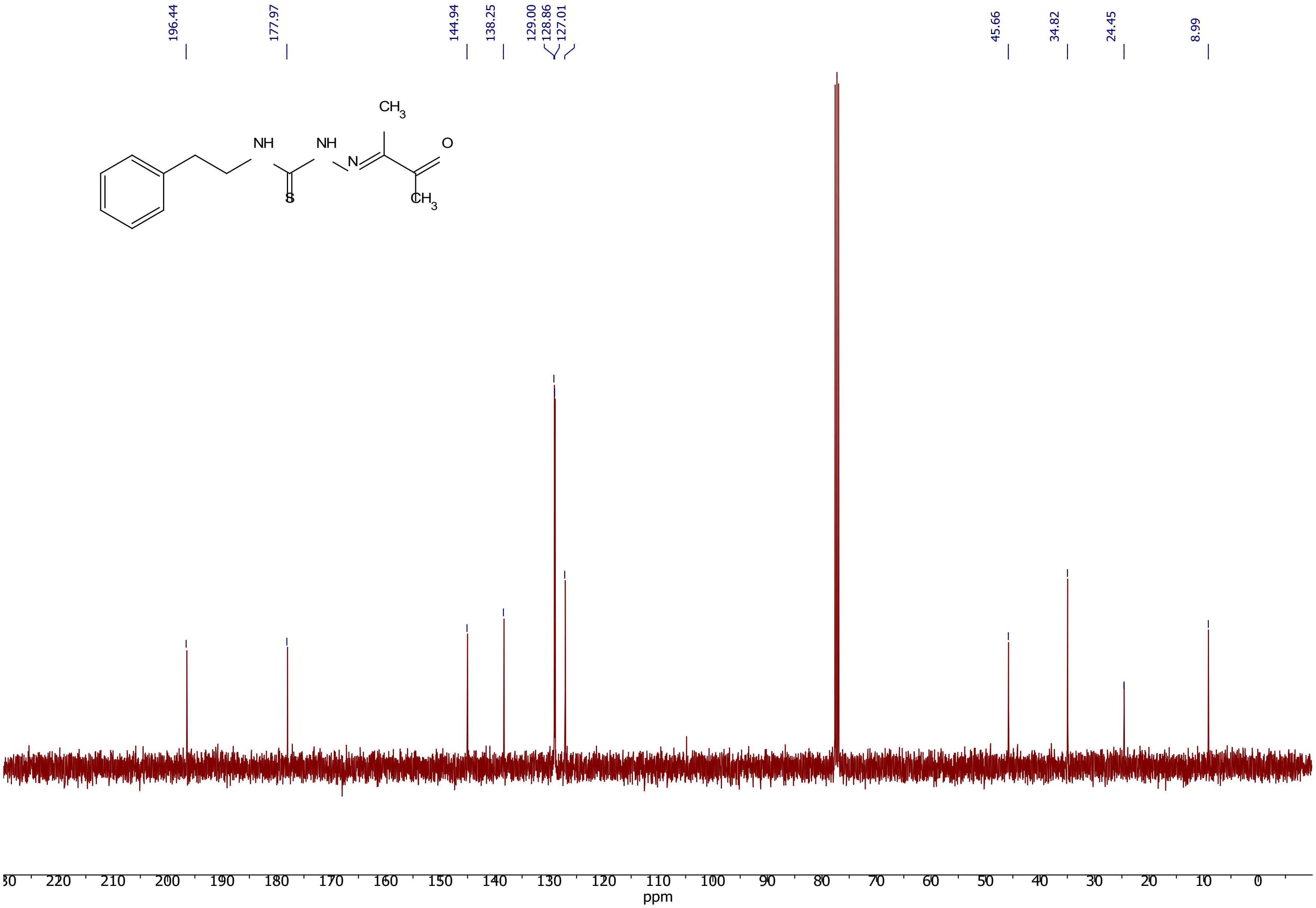

FTIR *2-(3-oxobutan-2-ylidene)-N-phenethylhydrazine-1-carbothioamide* (**2**).

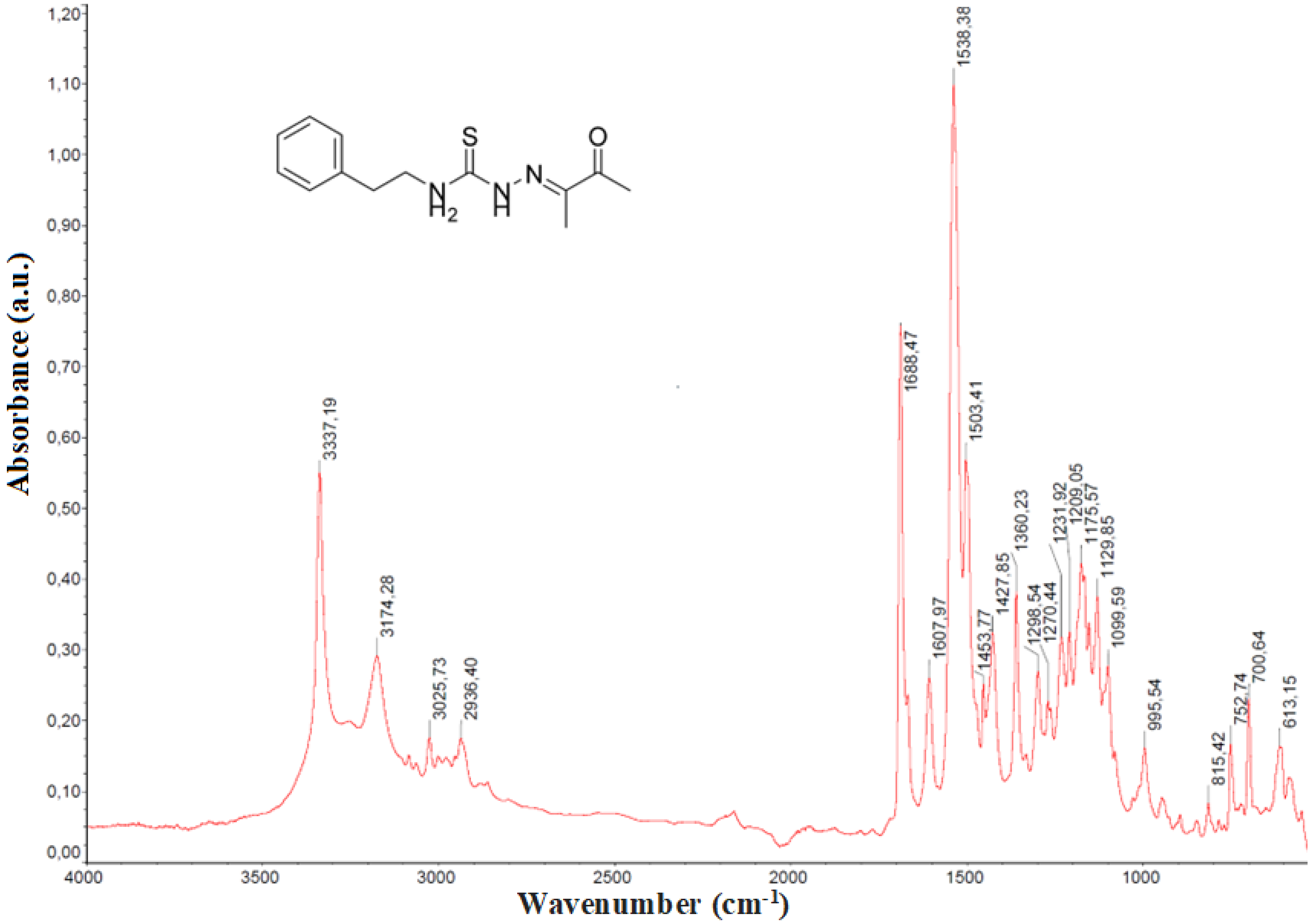

HRMS *2-(3-oxobutan-2-ylidene)-N-phenethylhydrazine-1-carbothioamide* (**2**).

Acquisition Parameter					
Source Type	ESI	Ion Polarity	Positive	Set Nebulizer	1.0 Bar
Focus	Active			Set Dry Heater	200 °C
Scan Begin	50 m/z	Set Capillary	4500 V	Set Dry Gas	4.0 l/min
Scan End	1800 m/z	Set End Plate Offset	-500 V	Set Divert Valve	Waste

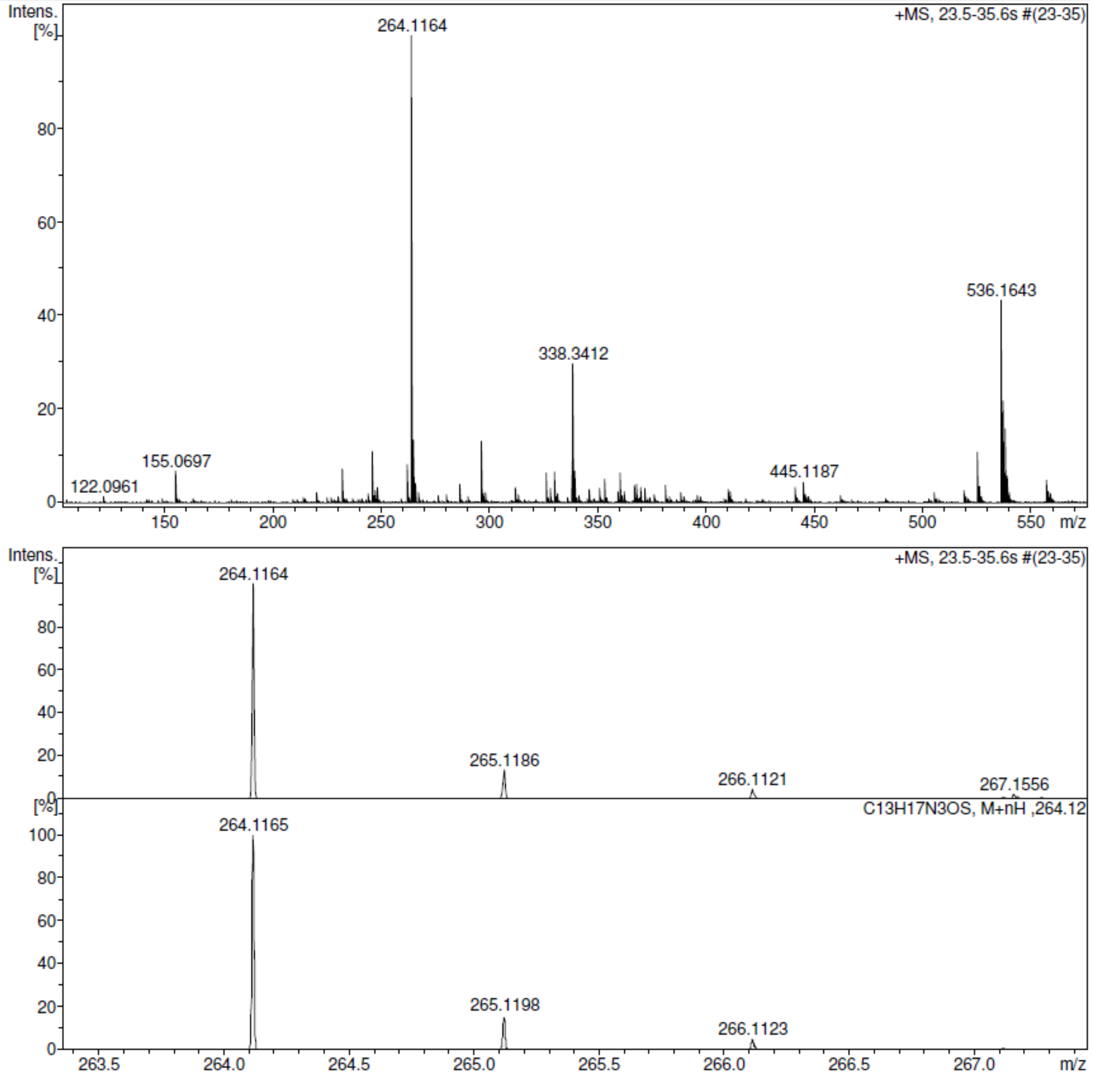

NMR ¹H *N-methyl-N-phenylhydrazinecarbothioamide* (**3**).

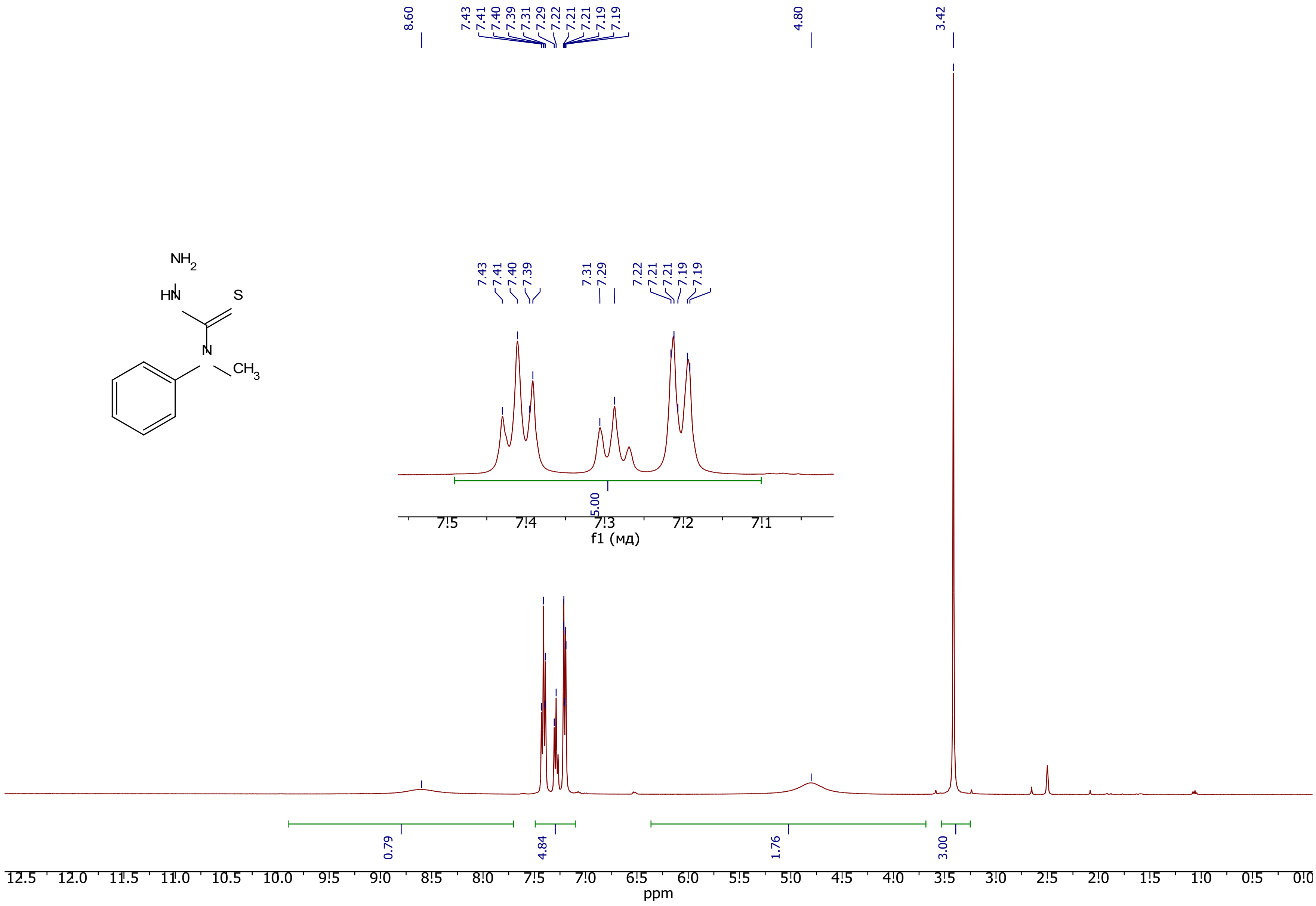

NMR ^{1}H *(E)-N-methyl-2-((E)-3-(2-(phenethylcarbamothioyl)hydrazineylidene)butan-2-ylidene)-N-phenylhydrazine-1-carbothioamide* (**4**).

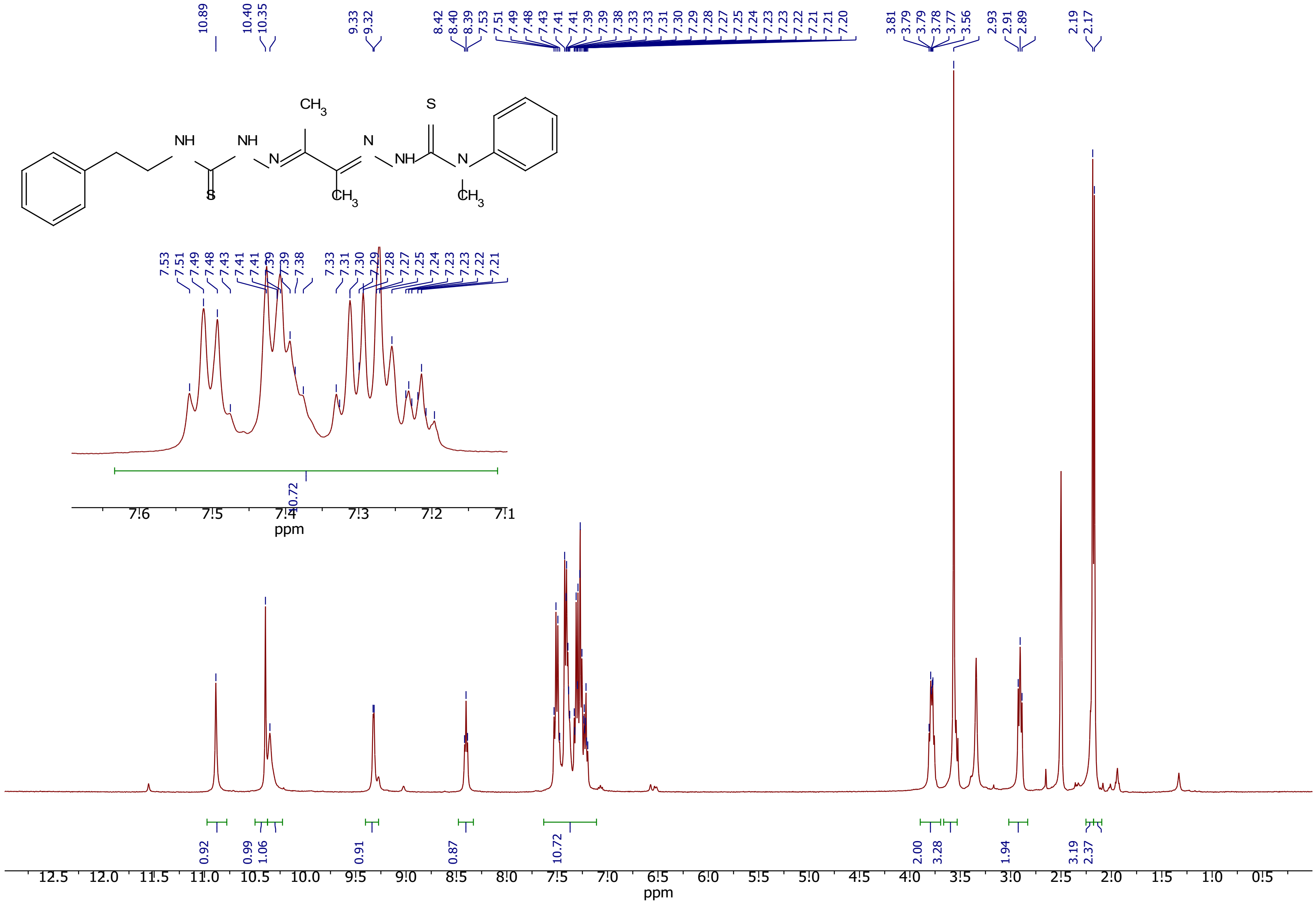

NMR ^{13}C *(E)-N-methyl-2-((E)-3-(2-(phenethylcarbamothioyl)hydrazineylidene)butan-2-ylidene)-N-phenylhydrazine-1-carbothioamide* (**4**).

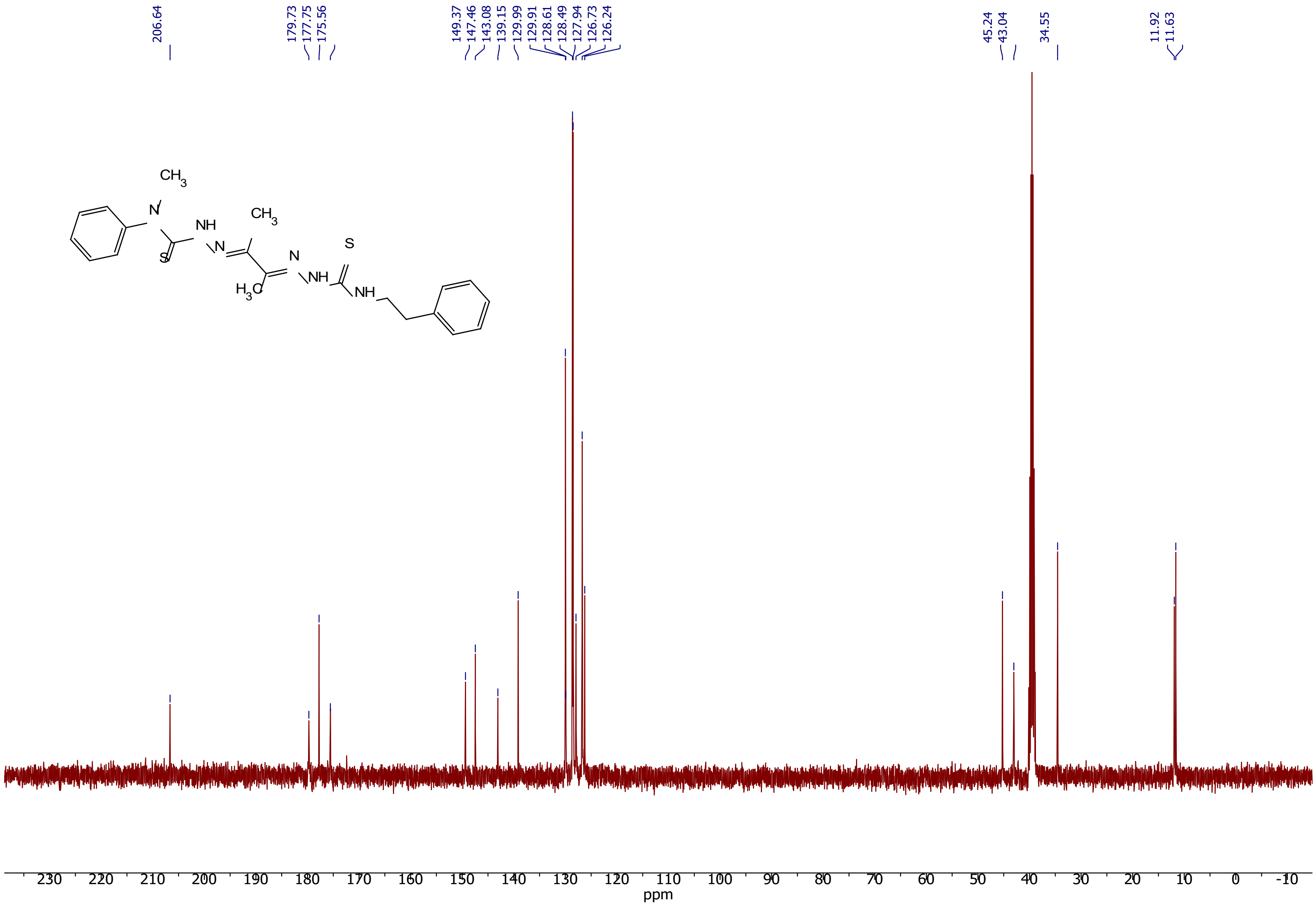

FTIR *(E)-N-methyl-2-((E)-3-(2-(phenethylcarbamothioyl)hydrazineylidene)butan-2-ylidene)-N-phenylhydrazine-1-carbothioamide* (**4**).

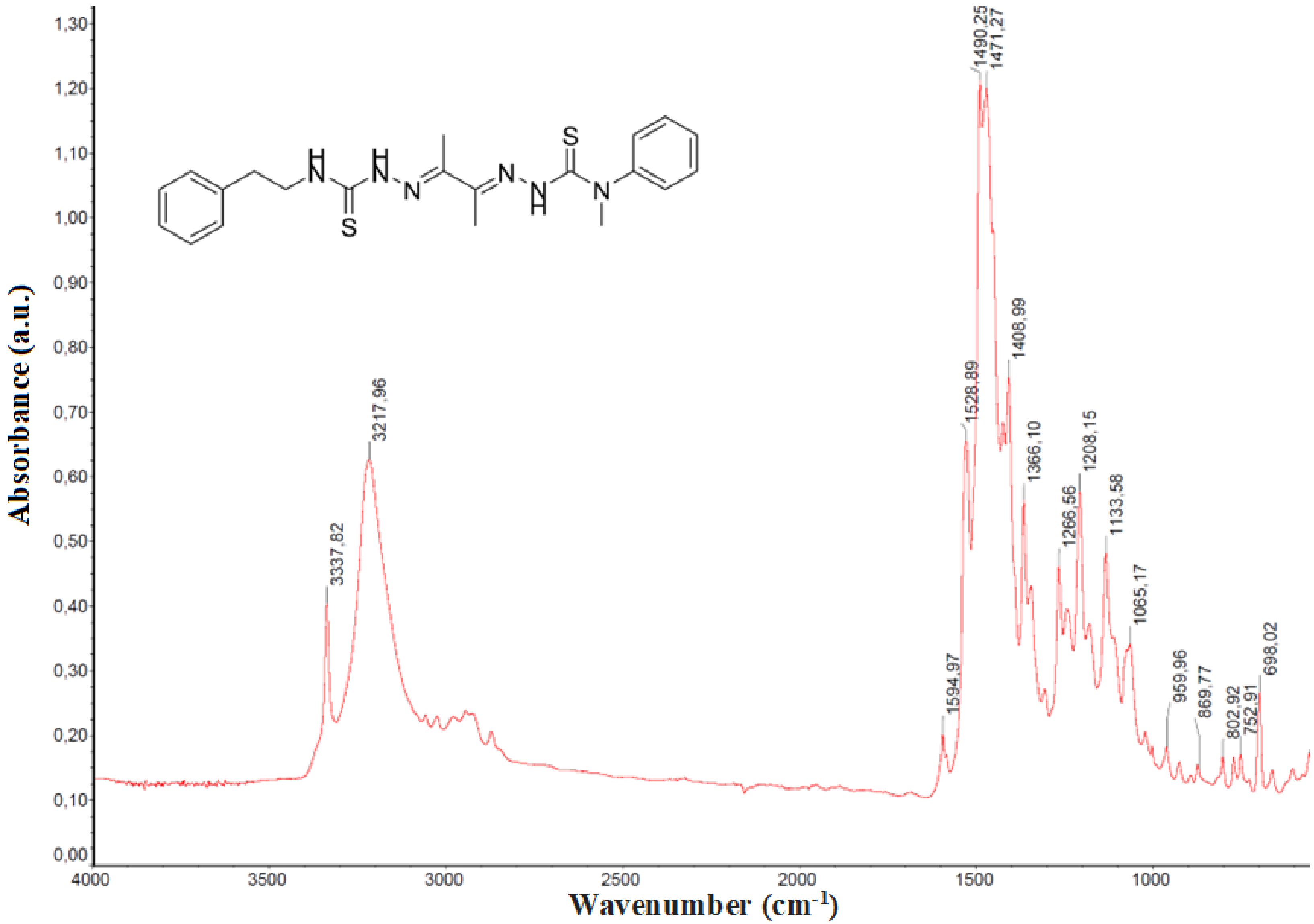

NMR ^{1}H *(E)-4-(4-aminostyryl)-N,N-dimethylaniline* (**5**).

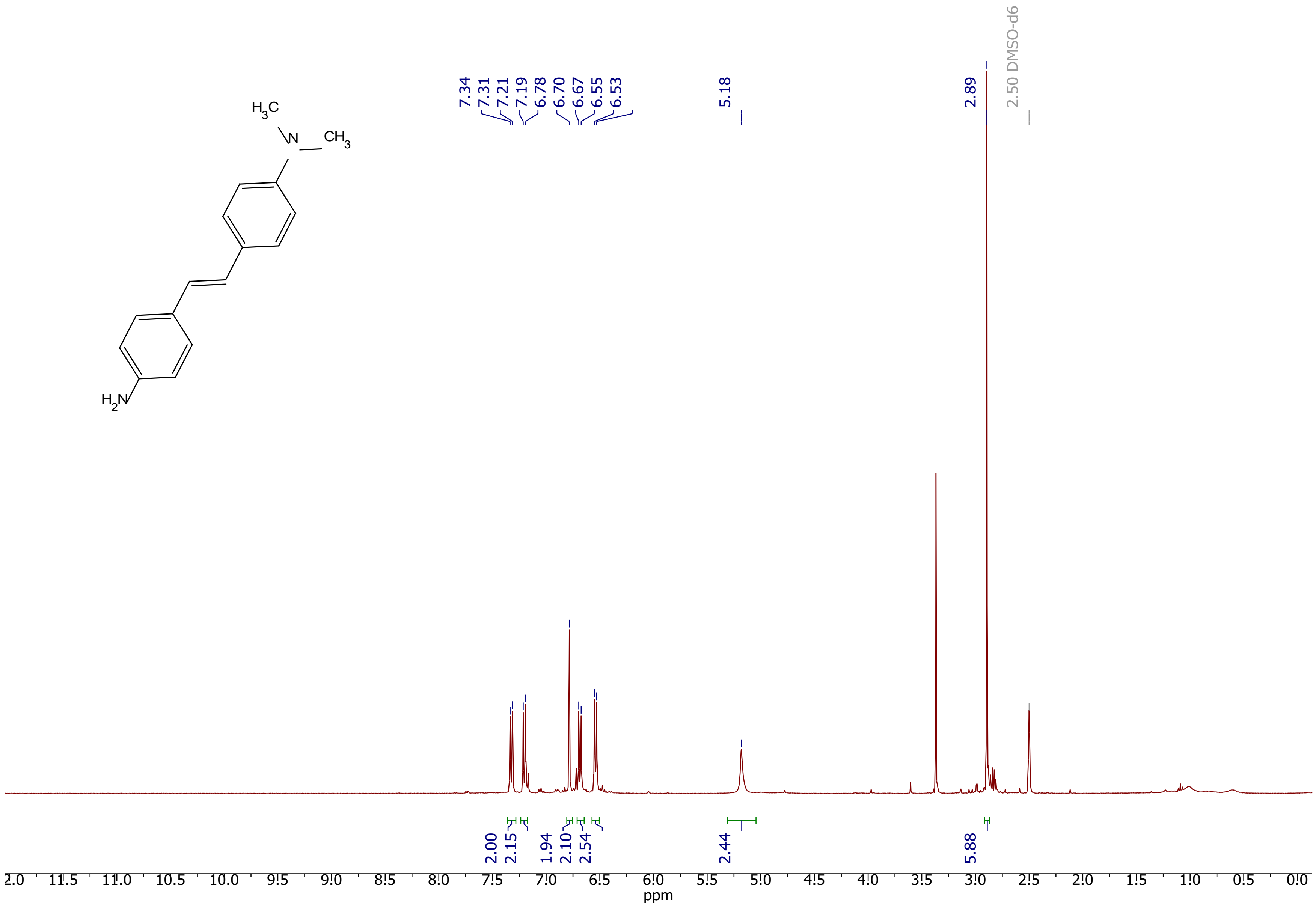

NMR ^{1}H *(E)-N-(4-((E)-4-(dimethylamino)styryl)phenyl)-2-((E)-3-(2-(phenethylcarbamothioyl)hydrazineylidene)butan-2-ylidene)hydrazine-1-carbothioamide* (**L6**).

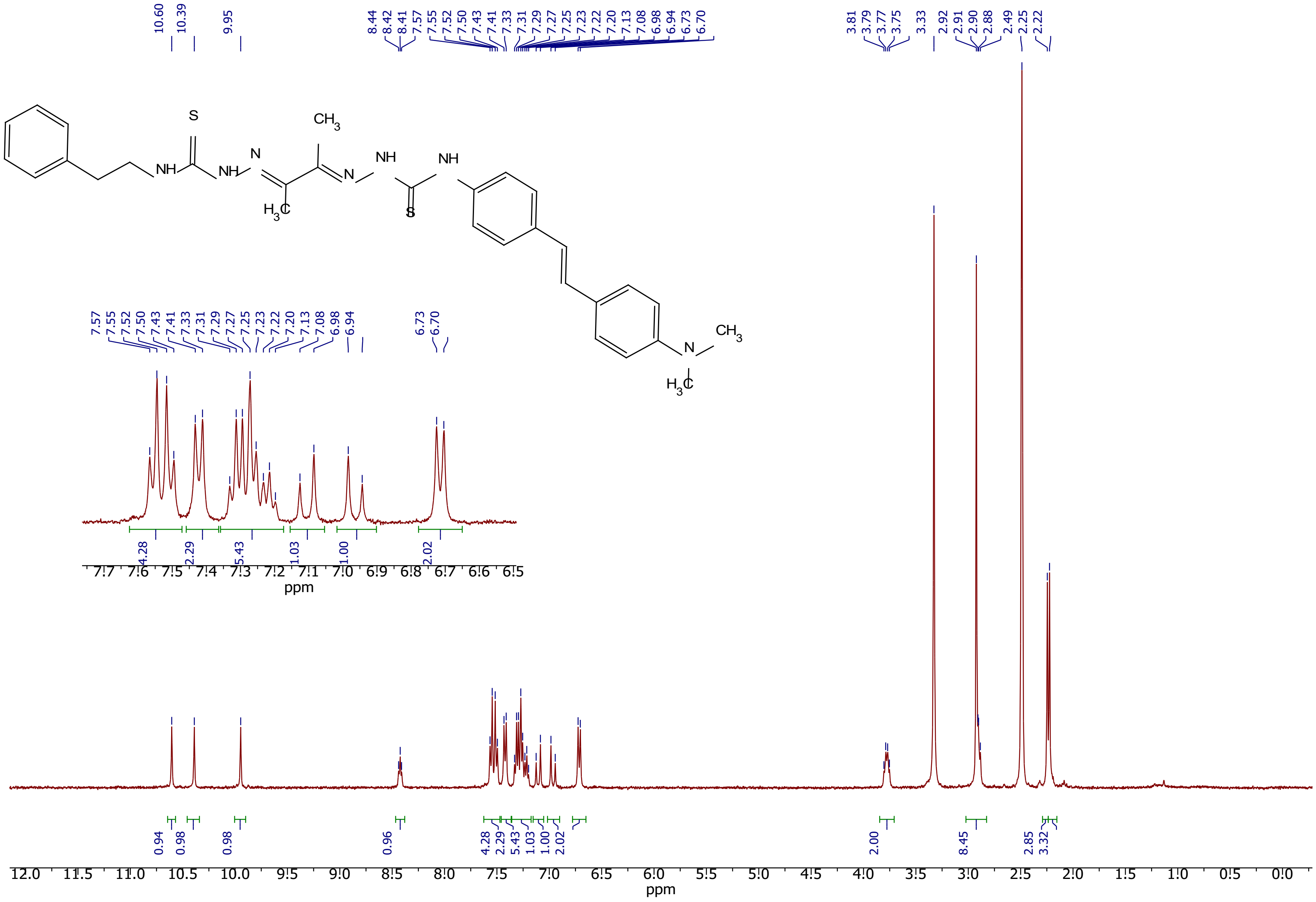

NMR ^{13}C *(E)-N-(4-((E)-4-(dimethylamino)styryl)phenyl)-2-((E)-3-(2-(phenethylcarbamothioyl)hydrazineylidene)butan-2-ylidene)hydrazine-1-carbothioamide* (**L6**).

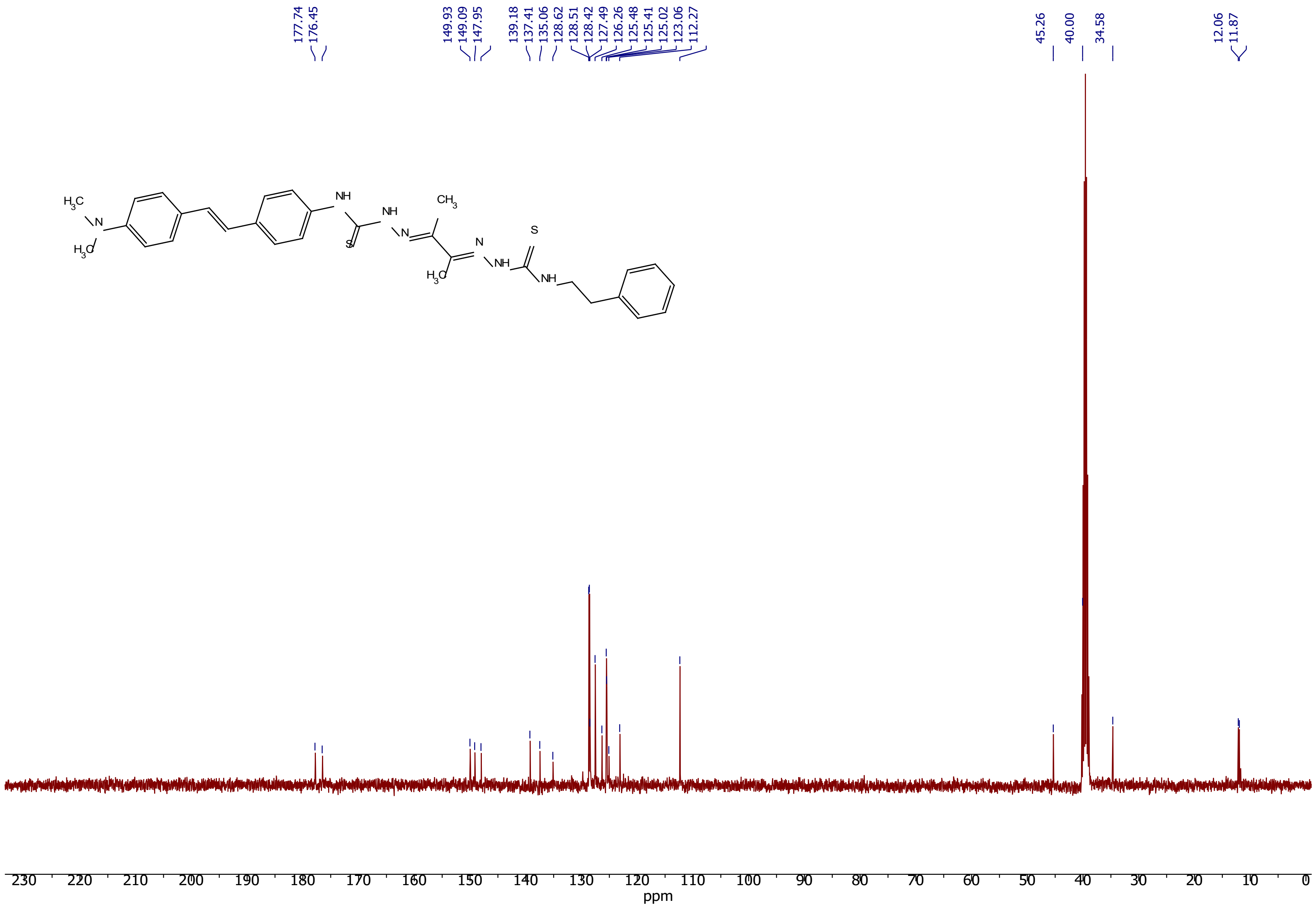

FTIR *(E)-N-(4-((E)-4-(dimethylamino)styryl)phenyl)-2-((E)-3-(2-(phenethylcarbamothioyl)hydrazineylidene)butan-2-ylidene)hydrazine-1-carbothioamide* (**L6**).

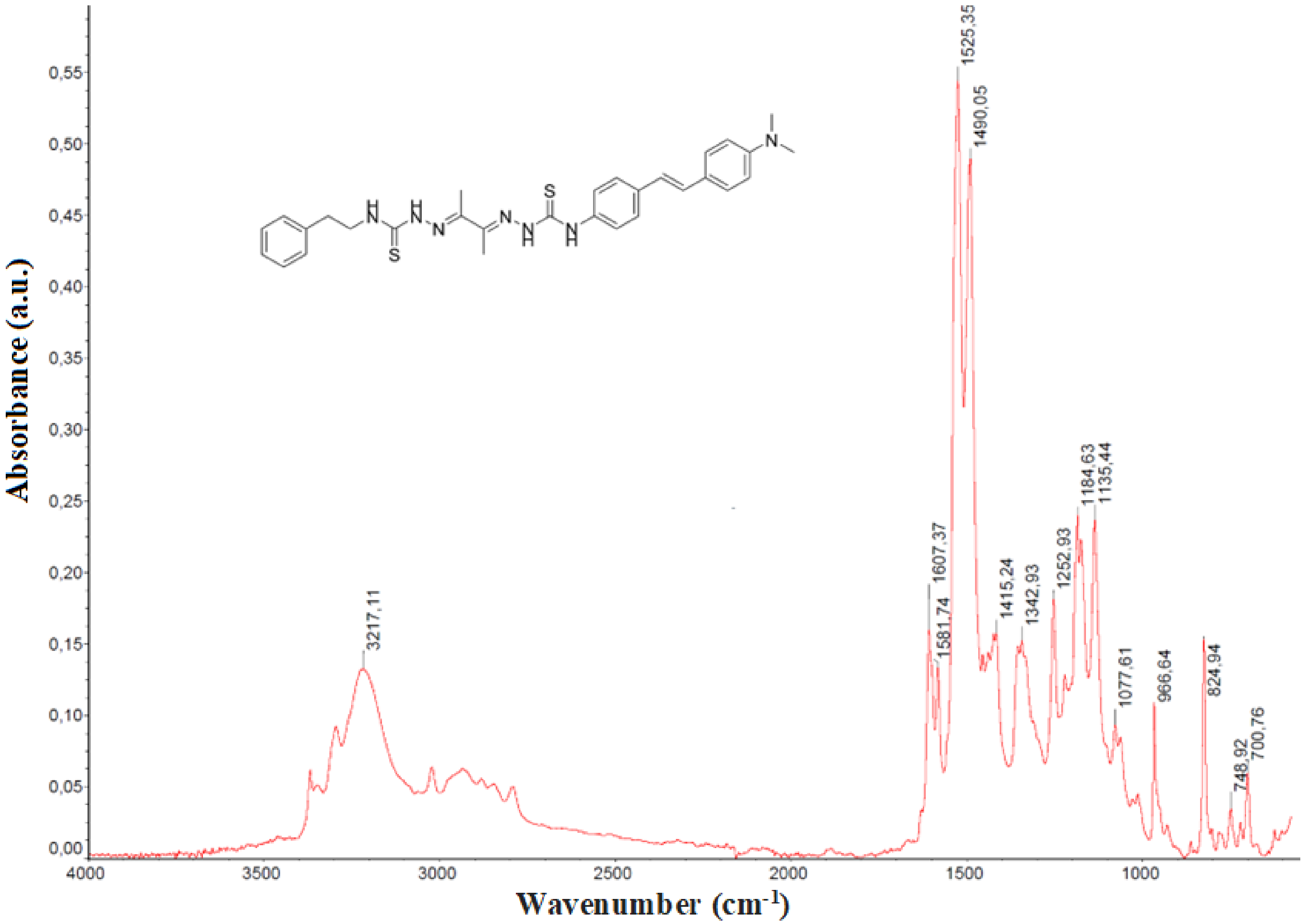

HRMS *(E)-N-(4-((E)-4-(dimethylamino)styryl)phenyl)-2-((E)-3-(2-(phenethylcarbamothioyl)hydrazineylidene)butan-2-ylidene)hydrazine-1-carbothioamide* (**L6**).

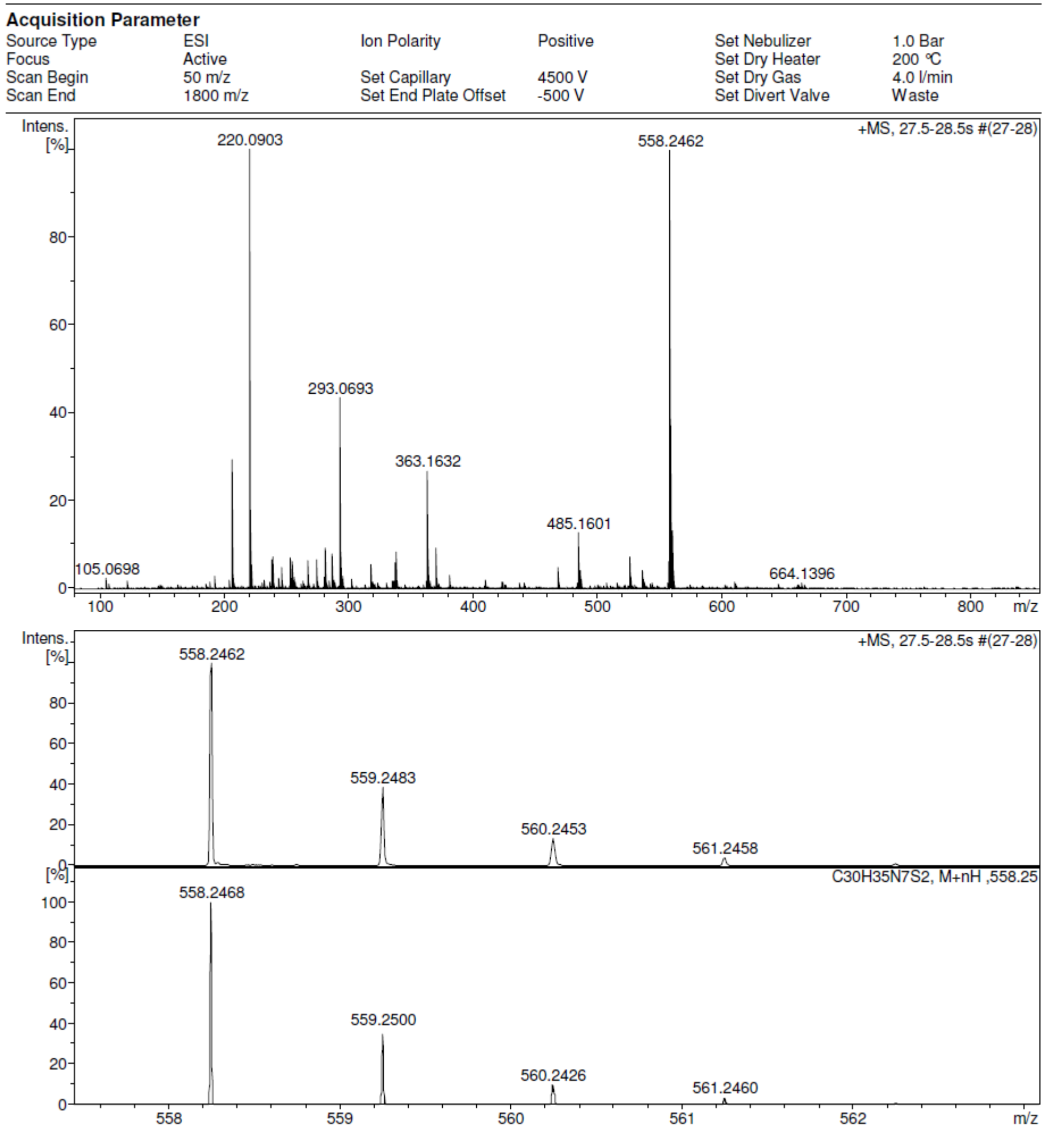

NMR ^{1}H *N-allylhydrazinecarbothioamide* (**6**).

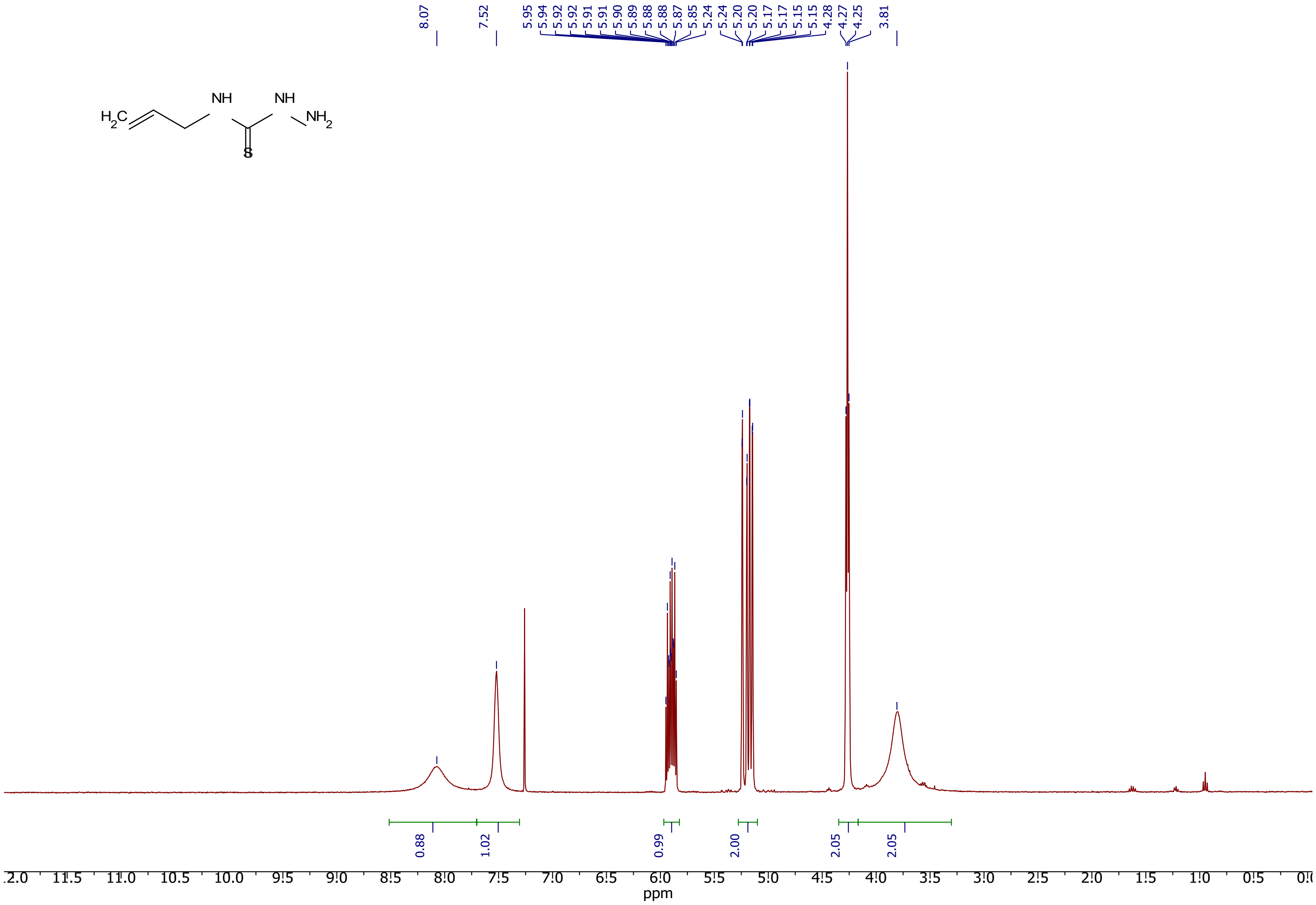

NMR ¹H *(E)-N-allyl-2-(3-oxobutan-2-ylidene)hydrazine-1-carbothioamide* (**7**).

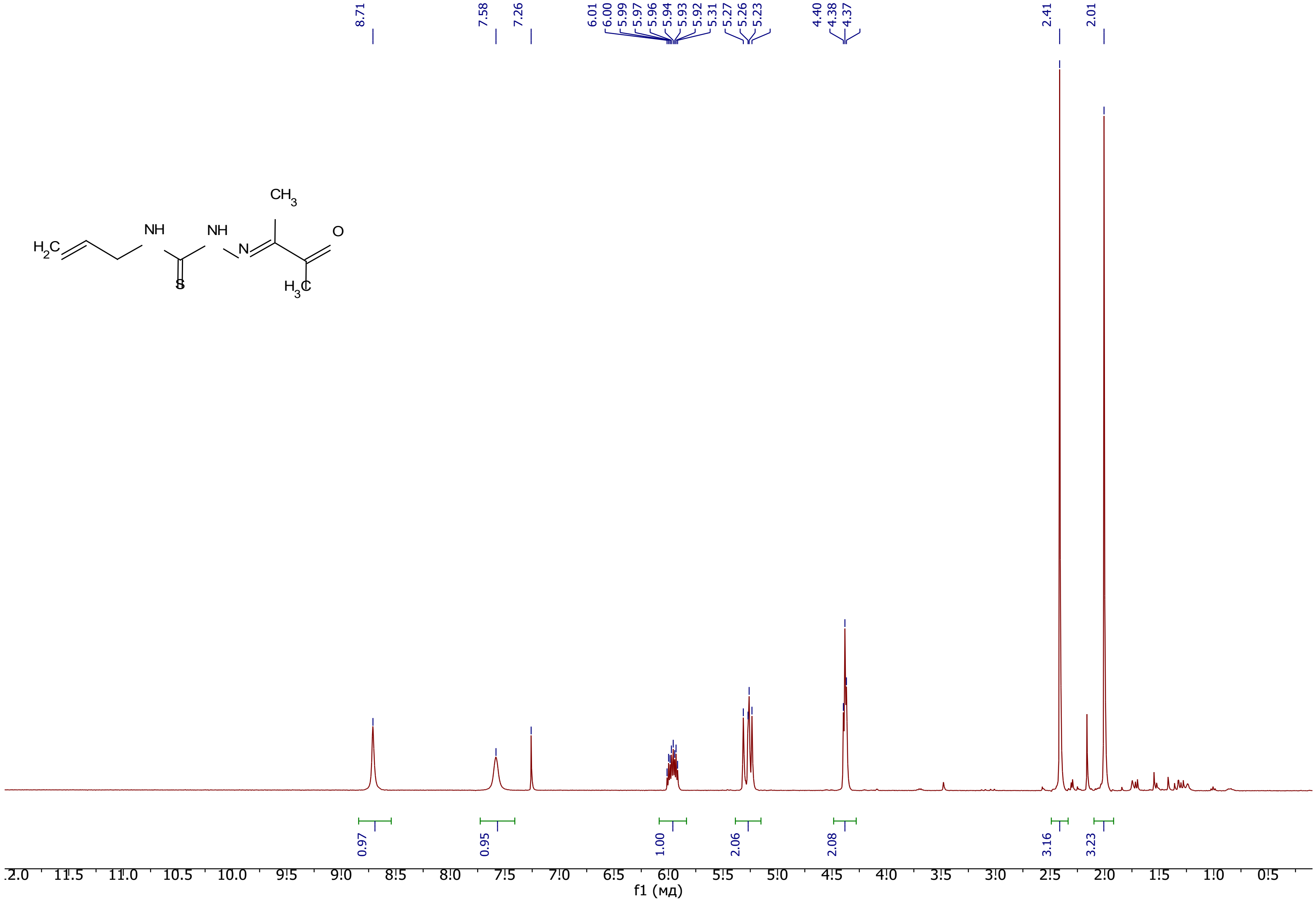

NMR ^{13}C *(E)-N-allyl-2-(3-oxobutan-2-ylidene)hydrazine-1-carbothioamide* (**7**).

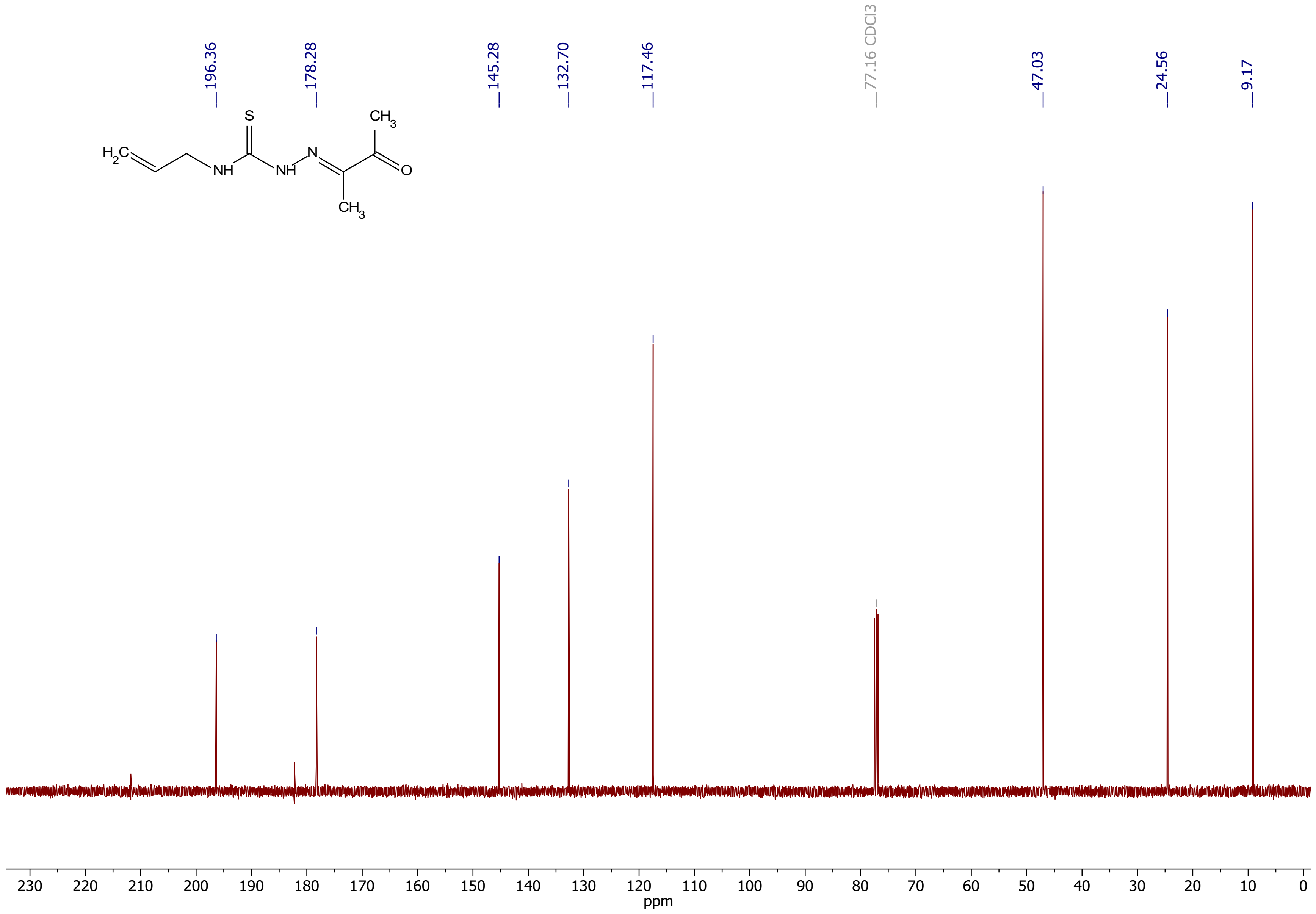

FTIR *(E)-N-allyl-2-(3-oxobutan-2-ylidene)hydrazine-1-carbothioamide* (**7**).

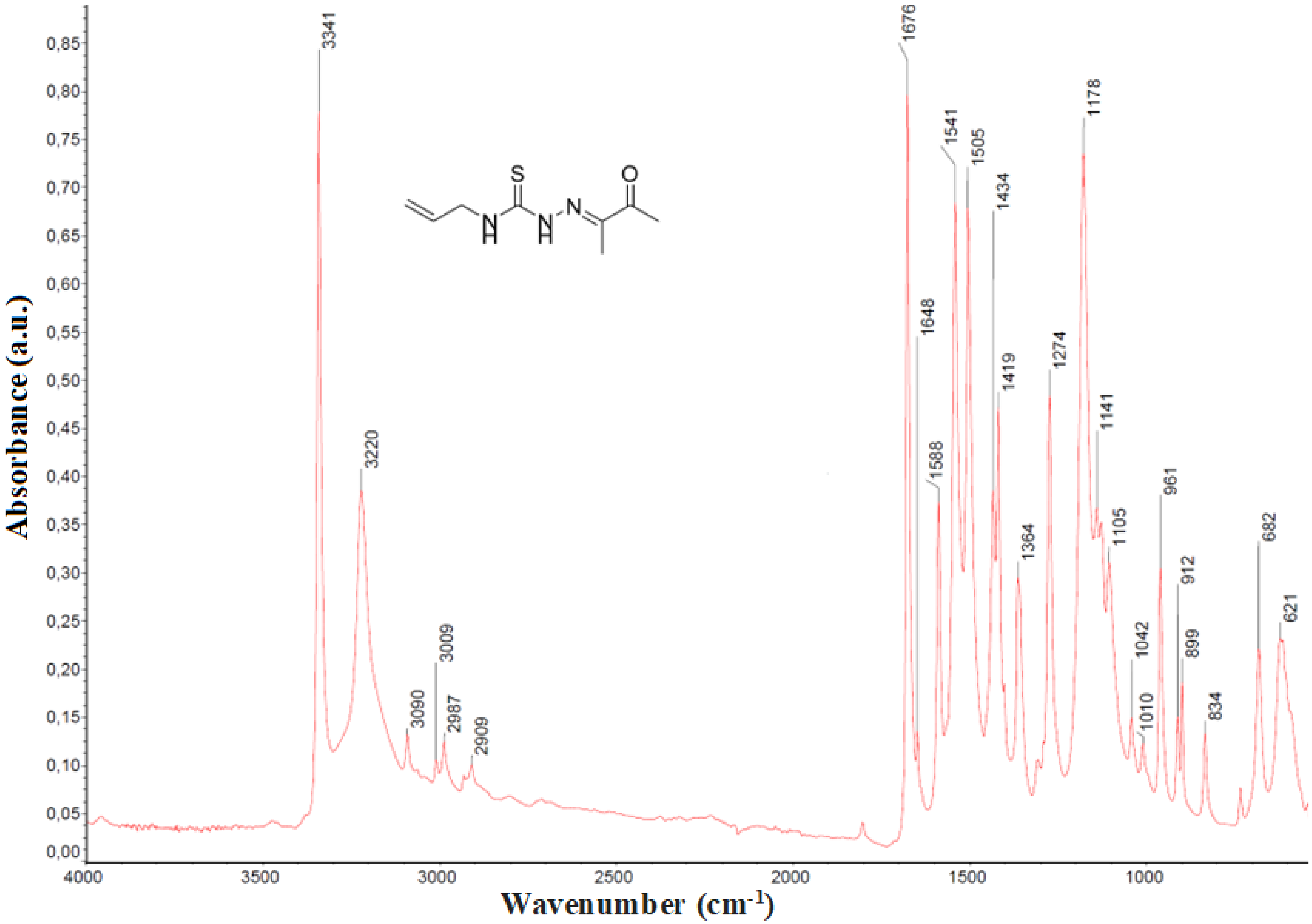

HRMS *(E)-N-allyl-2-(3-oxobutan-2-ylidene)hydrazine-1-carbothioamide* (**7**).

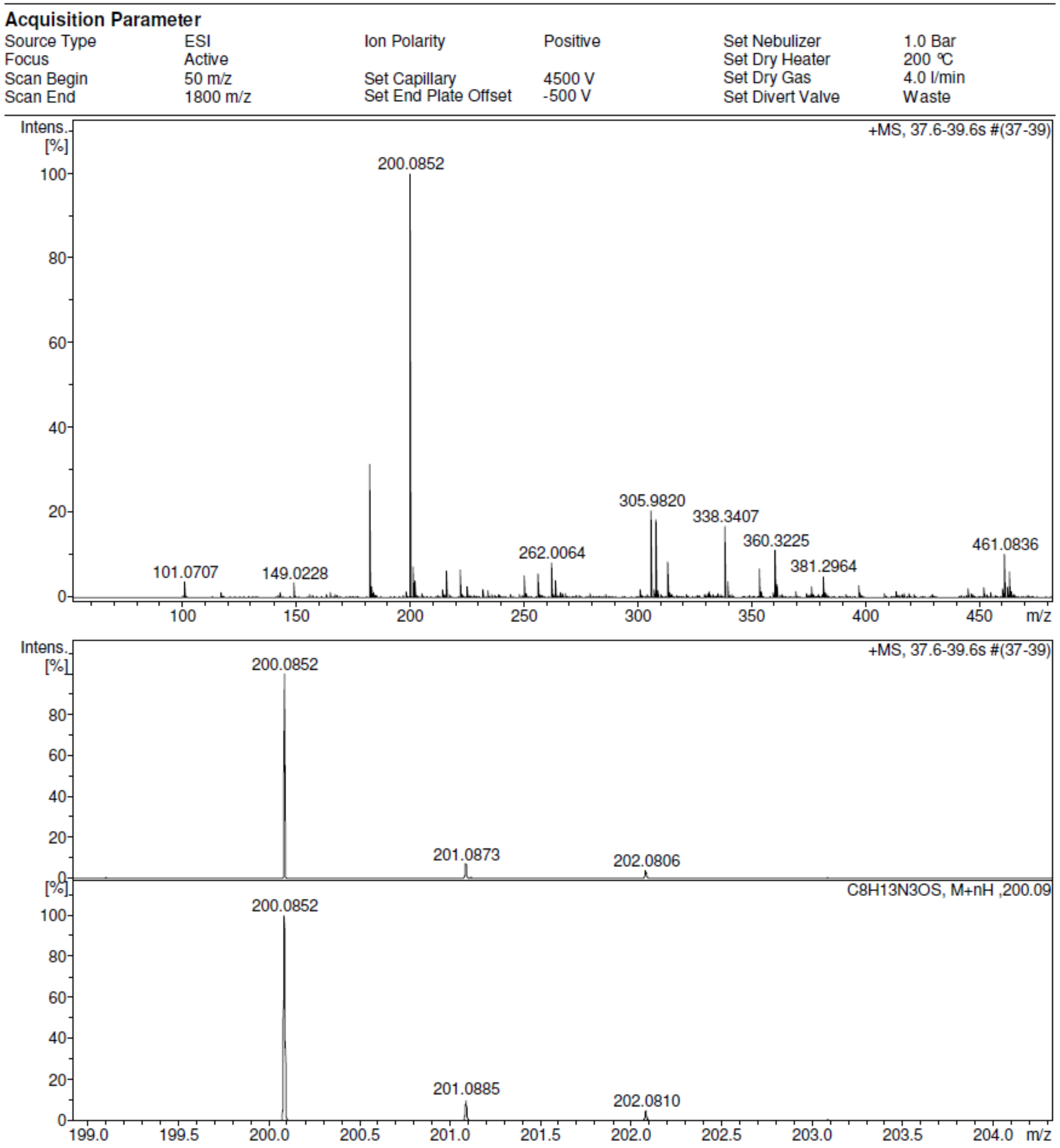

NMR ^{1}H *(E)-2-((E)-3-(2-(allylcarbamothioyl)hydrazineylidene)butan-2-ylidene)-N-methyl-N-phenylhydrazine-1-carbothioamide* (**8**).

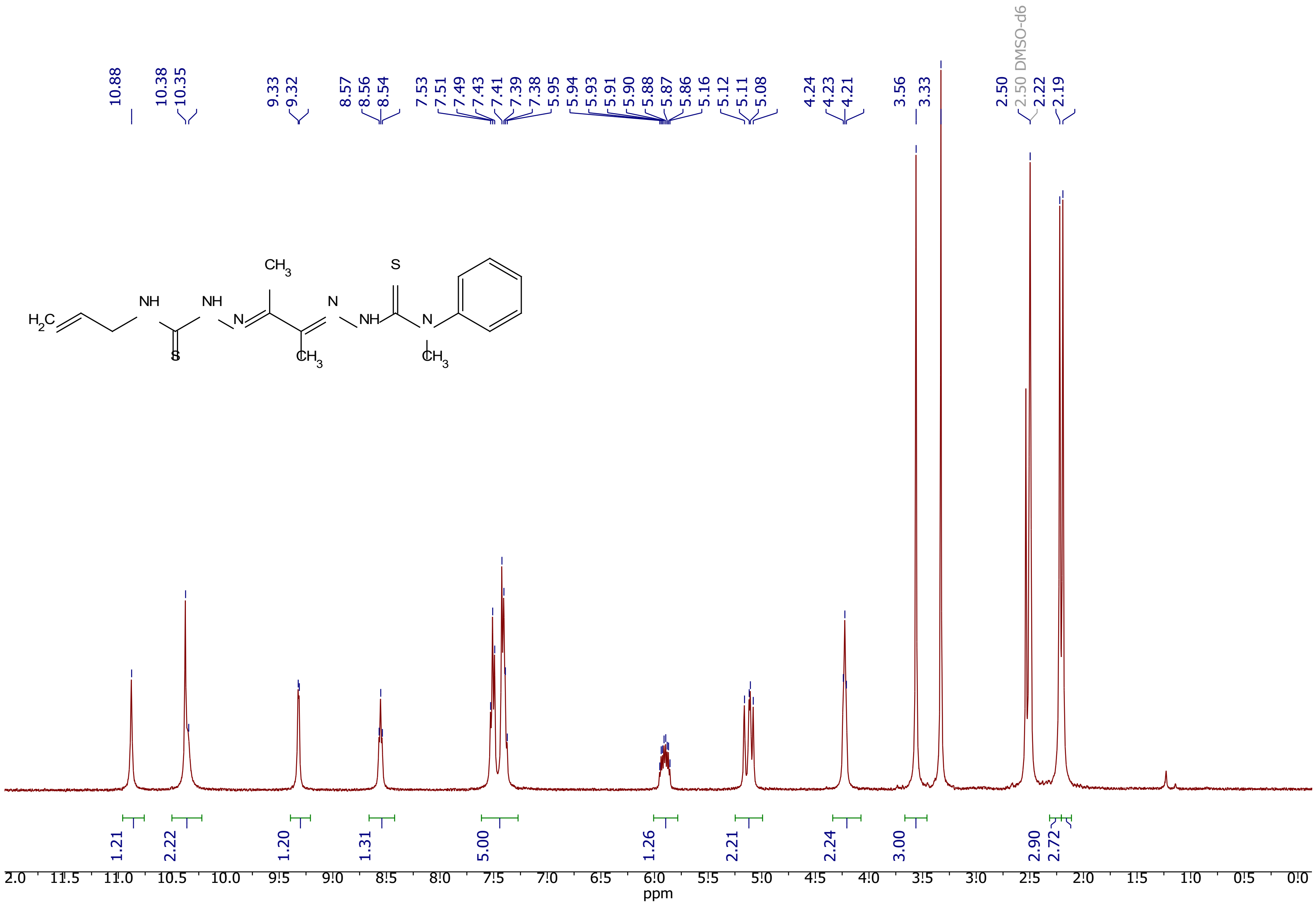

NMR ^{13}C *(E)-2-((E)-3-(2-(allylcarbamothioyl)hydrazineylidene)butan-2-ylidene)-N-methyl-N-phenylhydrazine-1-carbothioamide* (**8**).

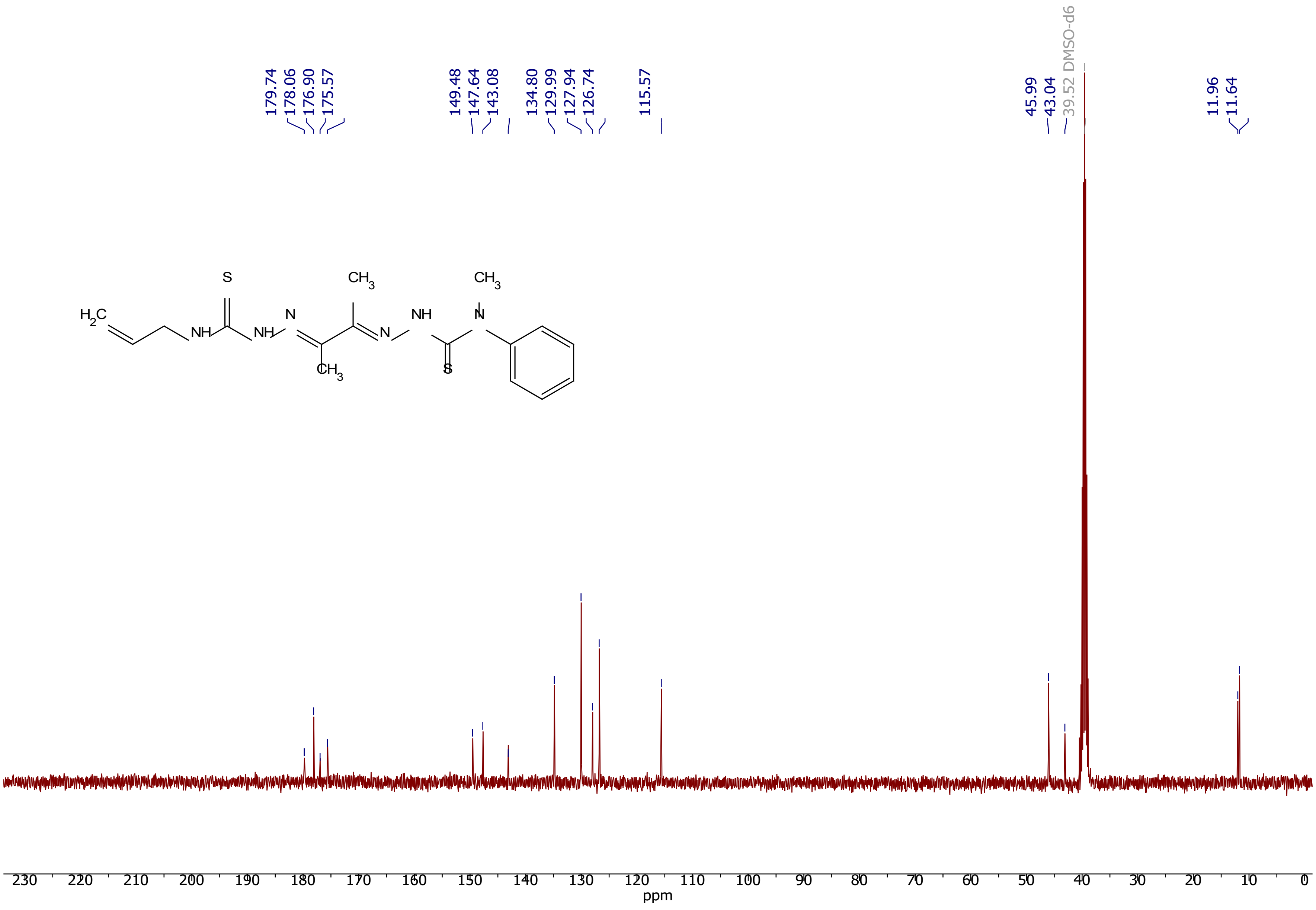

FTIR *(E)-2-((E)-3-(2-(allylcarbamothioyl)hydrazineylidene)butan-2-ylidene)-N-methyl-N-phenylhydrazine-1-carbothioamide* (**8**).

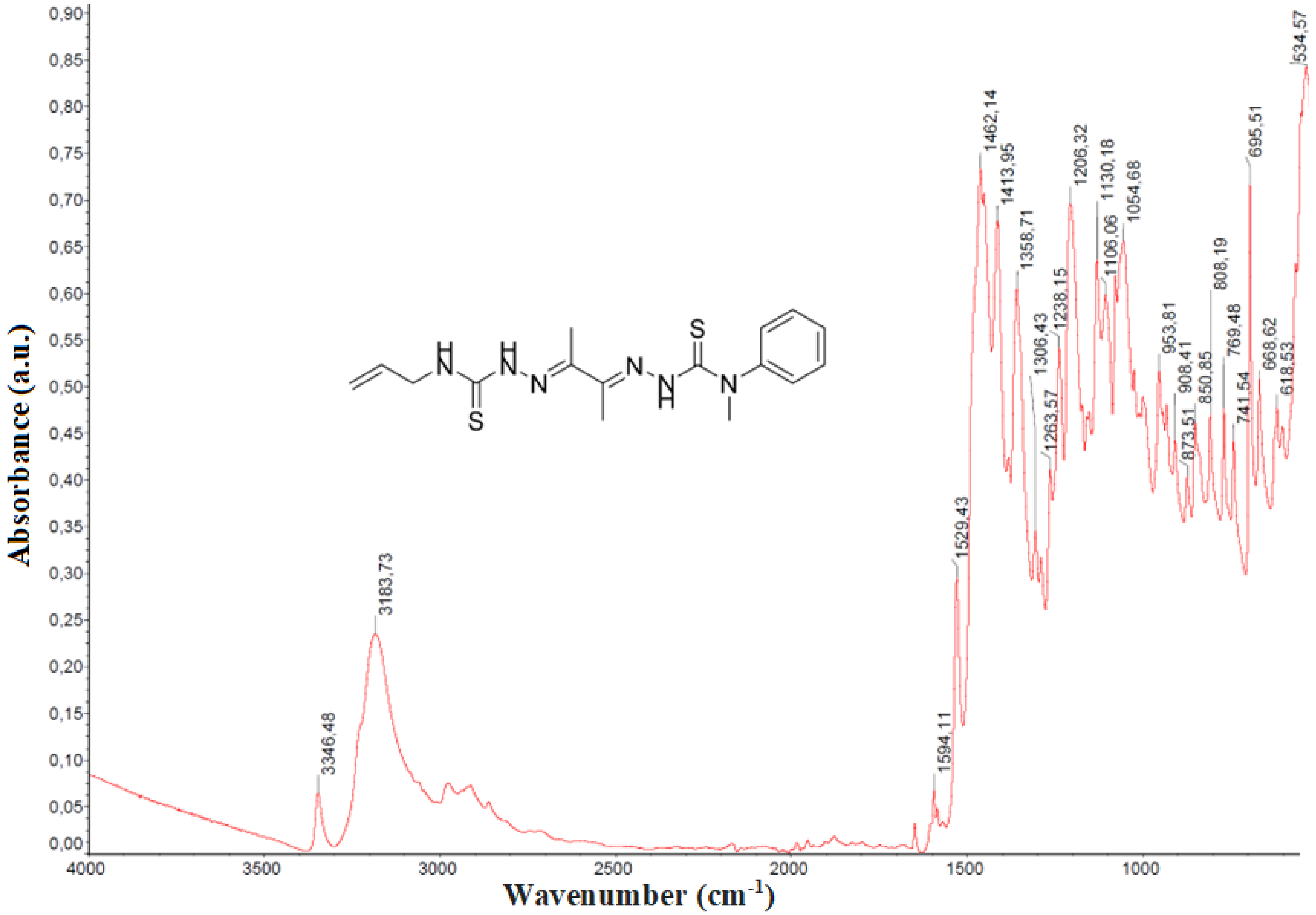

NMR ^{1}H *(E)-N-allyl-2-((E)-3-(2-((4-((E)-4-(dimethylamino)styryl)phenyl)carbamothioyl)hydrazineylidene)butan-2-ylidene)hydrazine-1-carbothioamide* (**L7**).

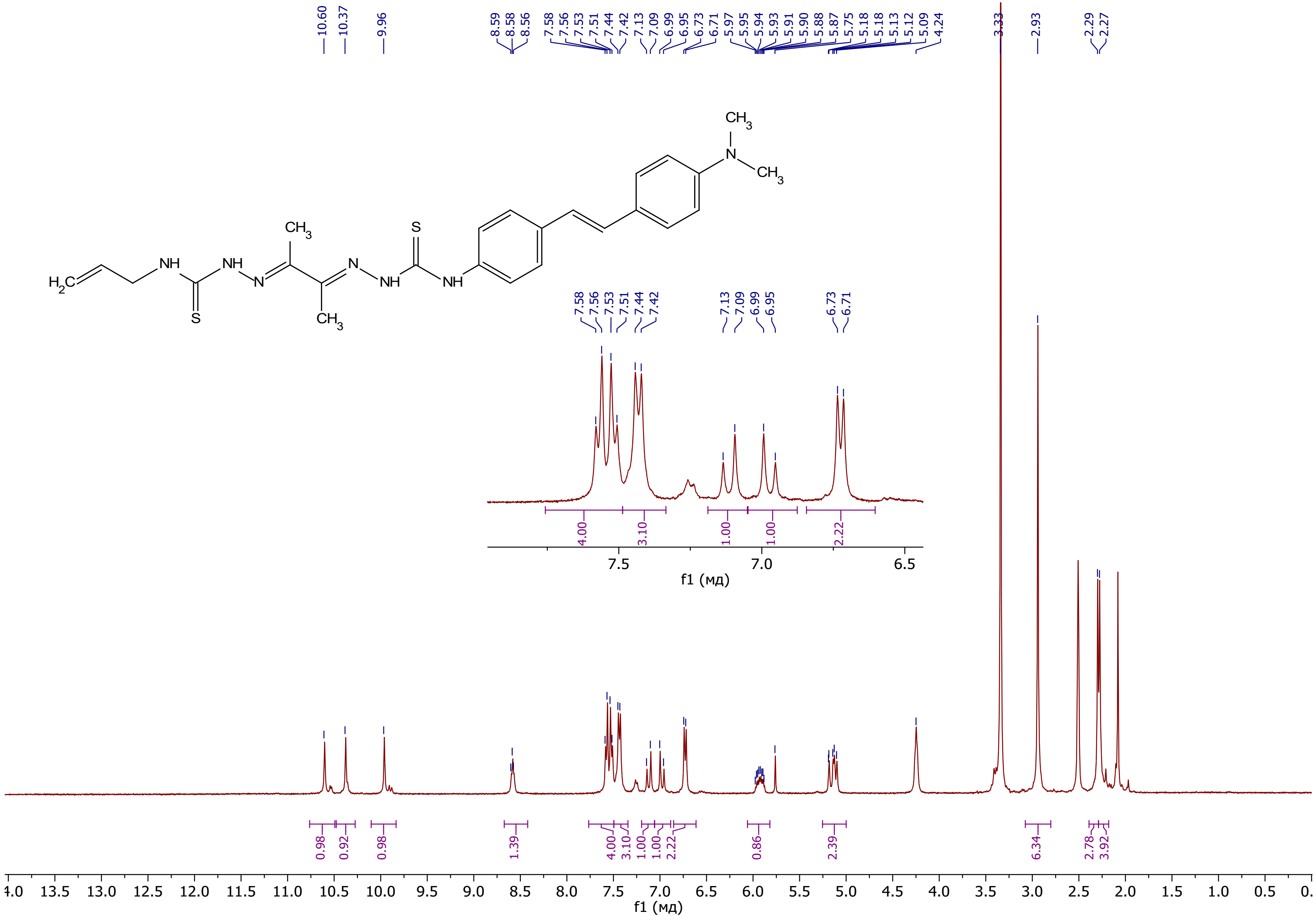

NMR ^{13}C *(E)-N-allyl-2-((E)-3-(2-((4-((E)-4-(dimethylamino)styryl)phenyl)carbamothioyl)hydrazineylidene)butan-2-ylidene)hydrazine-1-carbothioamide* (**L7**).

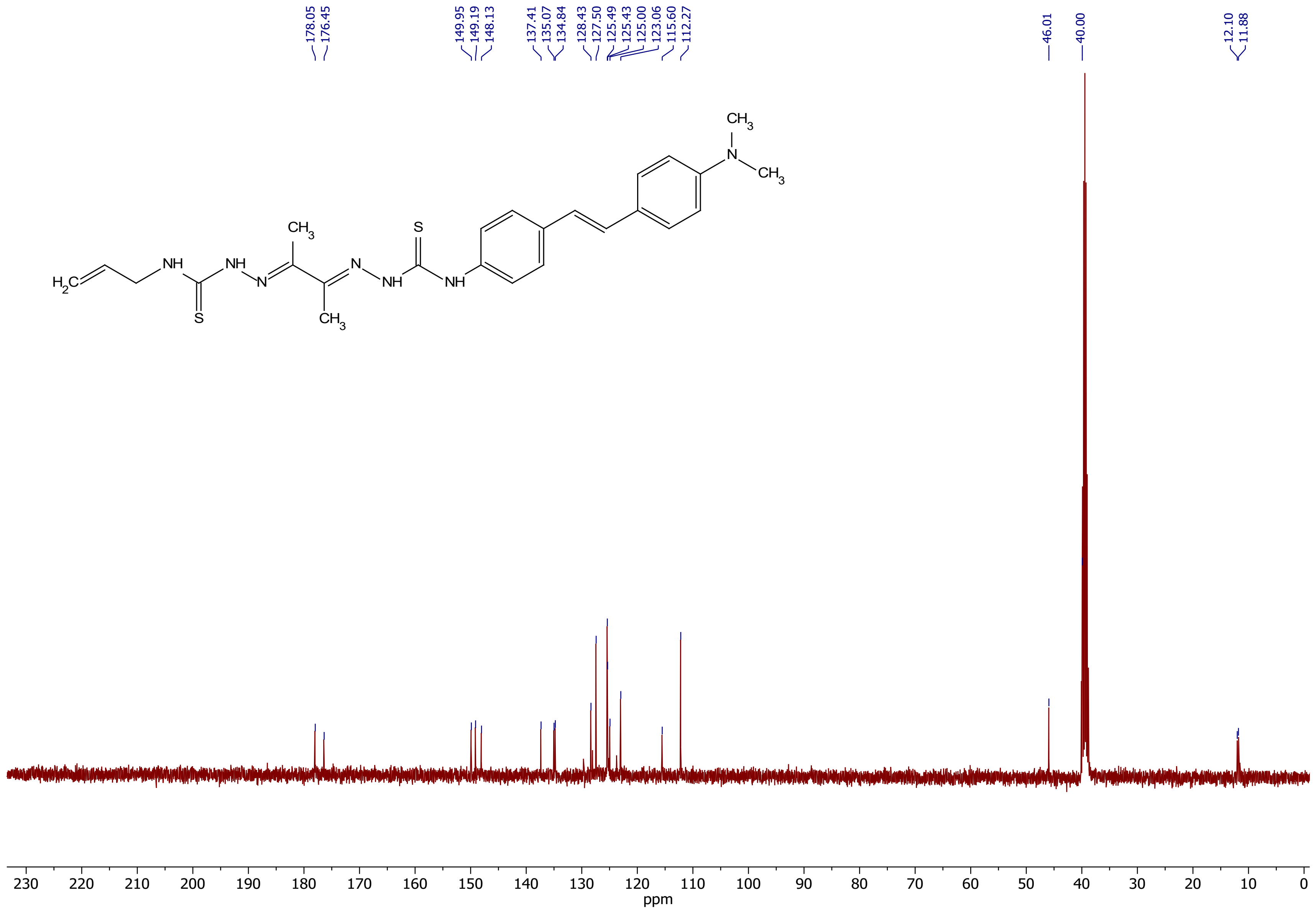

FTIR *(E)-N-allyl-2-((E)-3-(2-((4-((E)-4-(dimethylamino)styryl)phenyl)carbamothioyl)hydrazineylidene)butan-2-ylidene)hydrazine-1-carbothioamide* (**L7**).

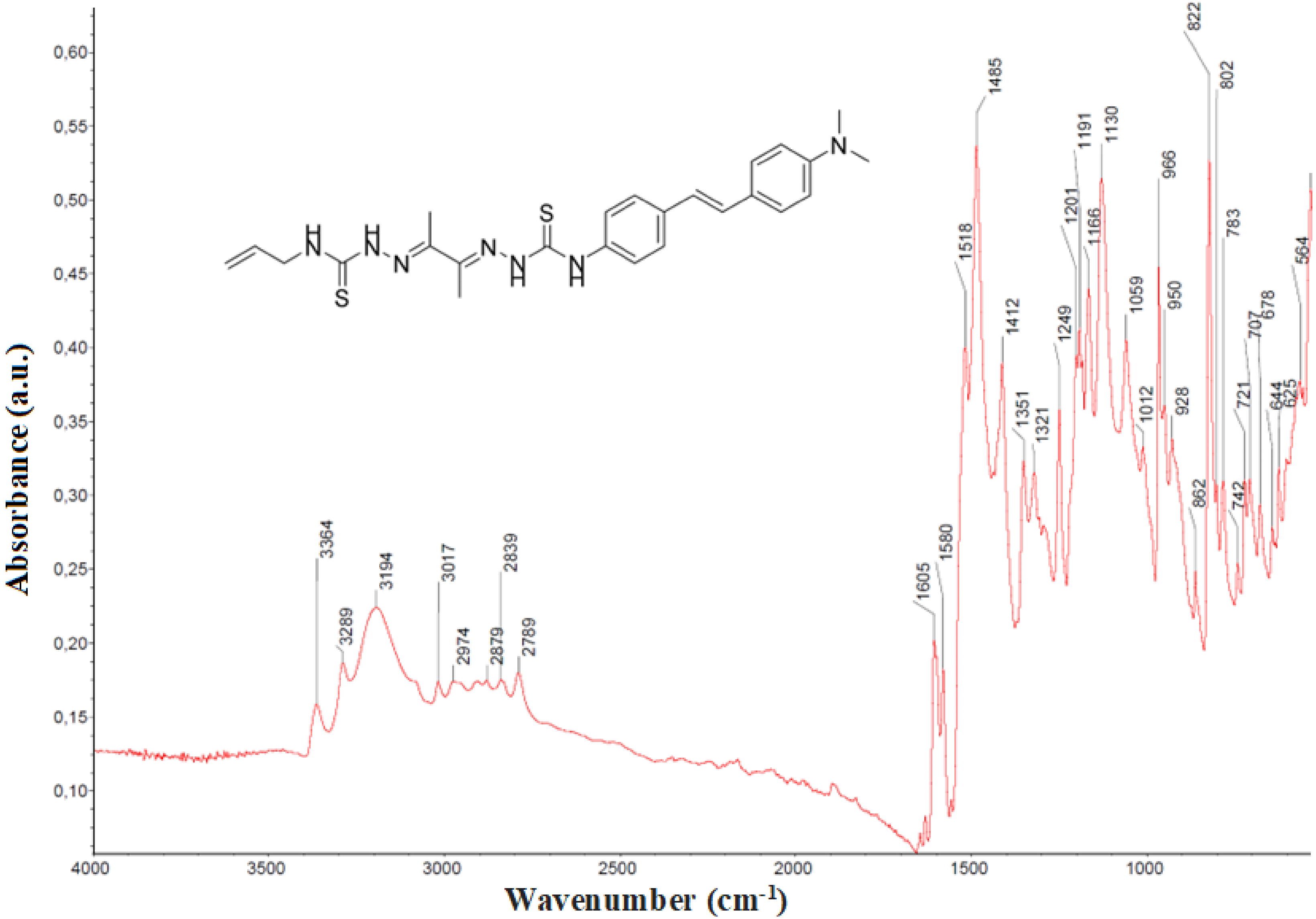

HRMS *(E)-N-allyl-2-((E)-3-(2-((4-((E)-4-(dimethylamino)styryl)phenyl)carbamothioyl)hydrazineylidene)butan-2-ylidene)hydrazine-1-carbothioamide* (**L7**).

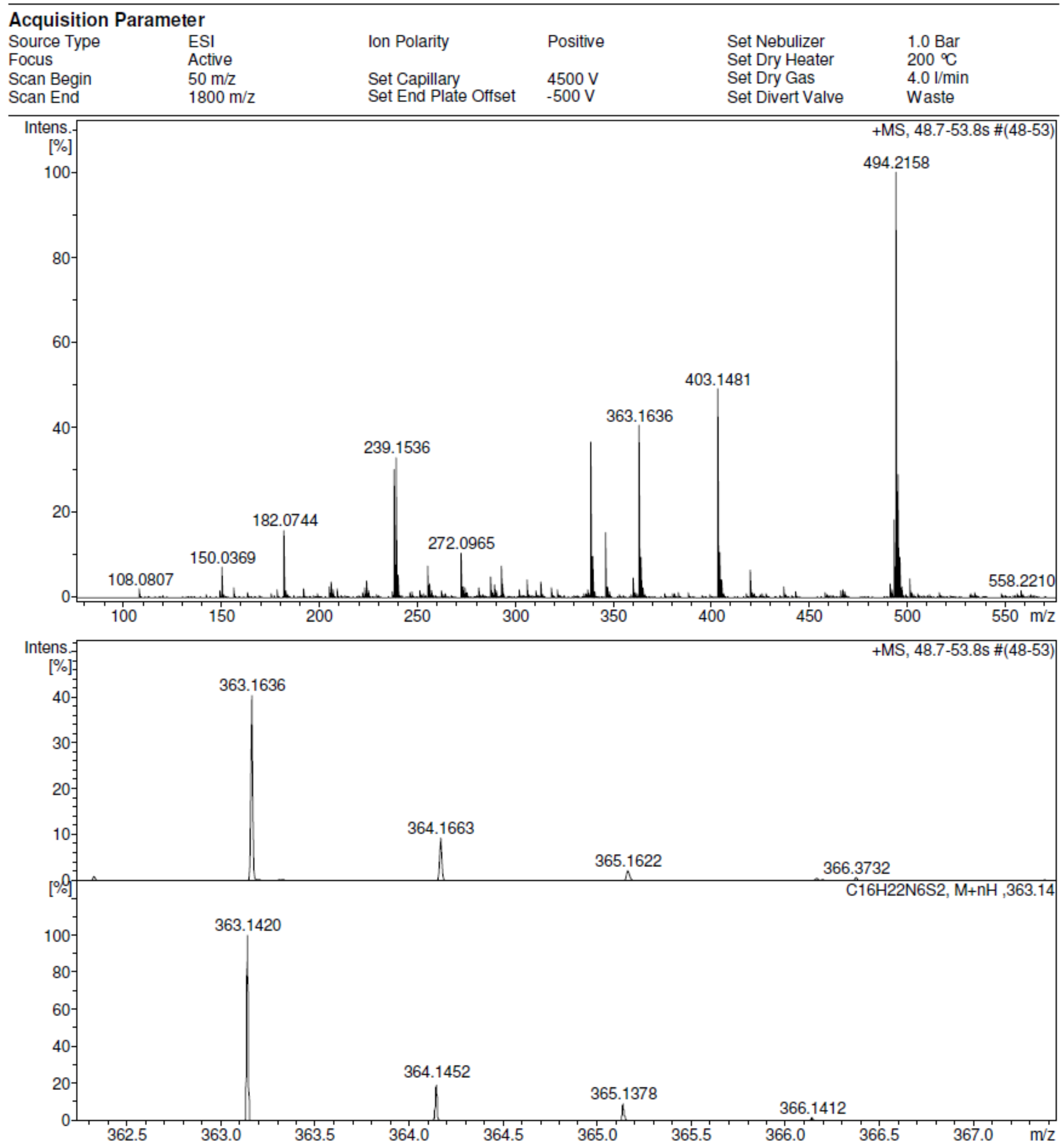

NMR ^{1}H *(E)-N-allyl-2-((E)-3-hydrazineylidenebutan-2-ylidene)hydrazine-1-carbothioamide* (**9**).

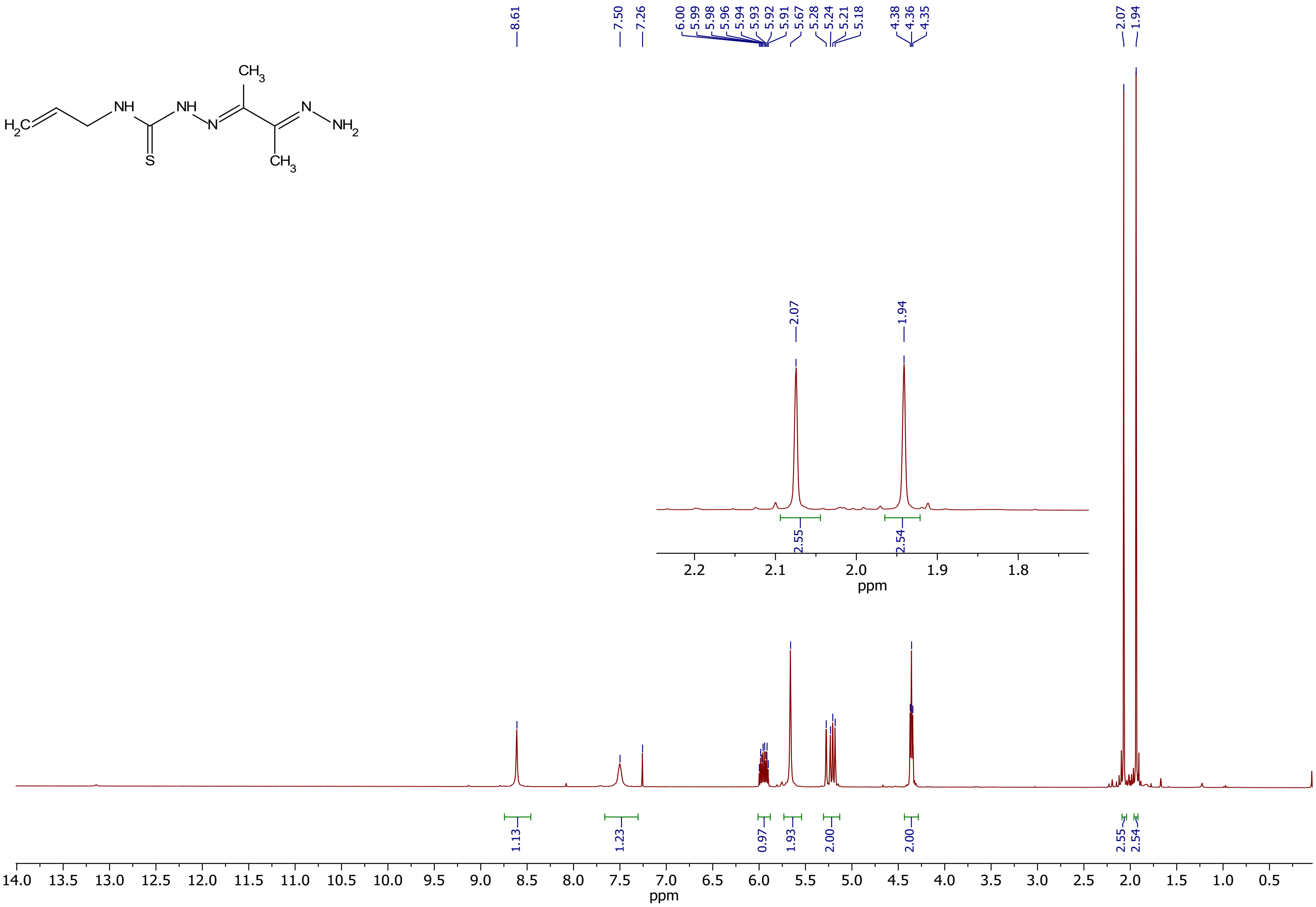

NMR ¹³C *(E)-N-allyl-2-((E)-3-hydrazineylidenebutan-2-ylidene)hydrazine-1-carbothioamide* (**9**).

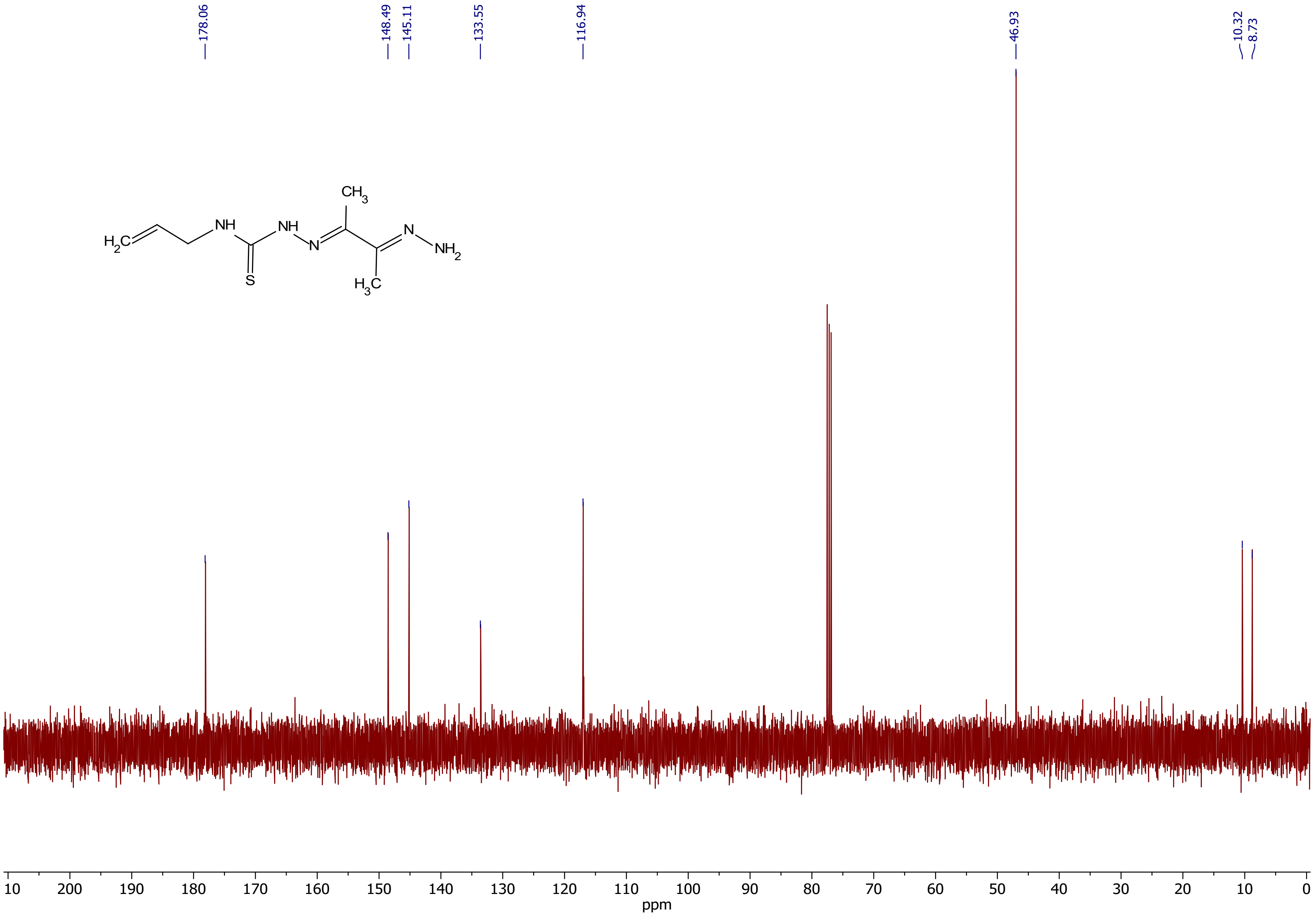

FTIR *(E)-N-allyl-2-((E)-3-hydrazineylidenebutan-2-ylidene)hydrazine-1-carbothioamide* (**9**).

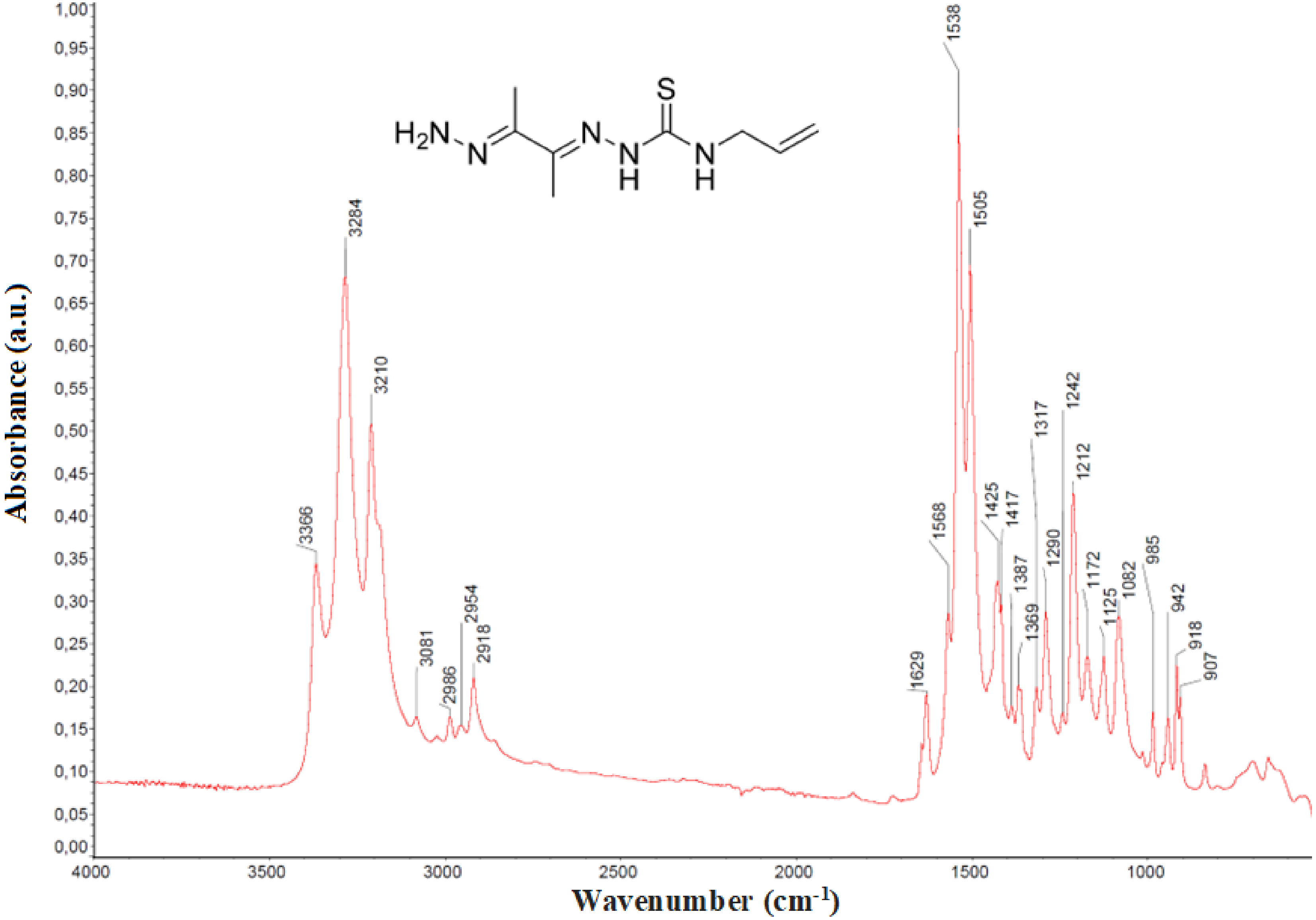

HRMS *(E)-N-allyl-2-((E)-3-hydrazineylidenebutan-2-ylidene)hydrazine-1-carbothioamide* (**9**).

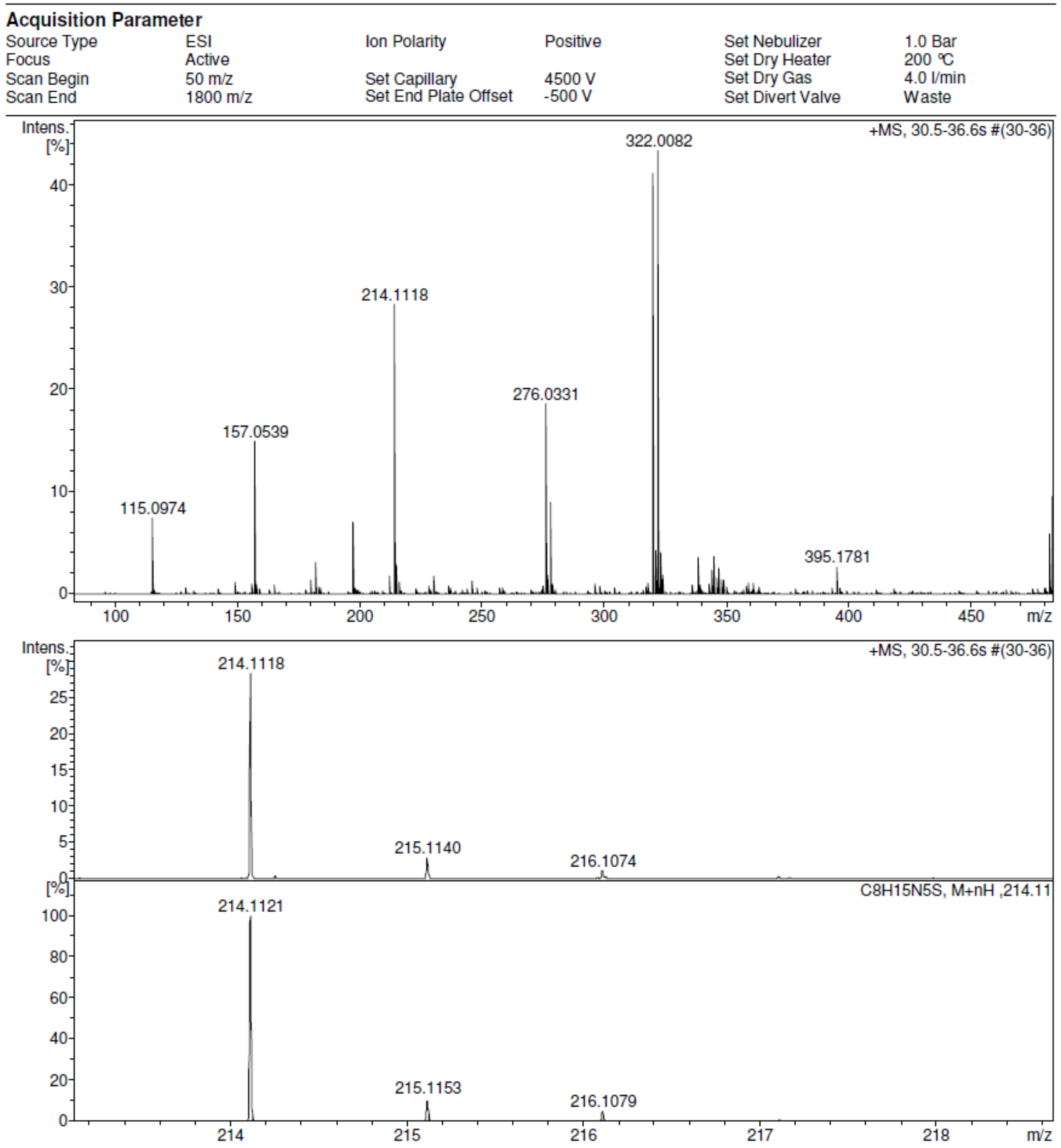

NMR ^{1}H *2-(4-isothiocyanatophenyl)benzo[d]thiazole* (**10**).

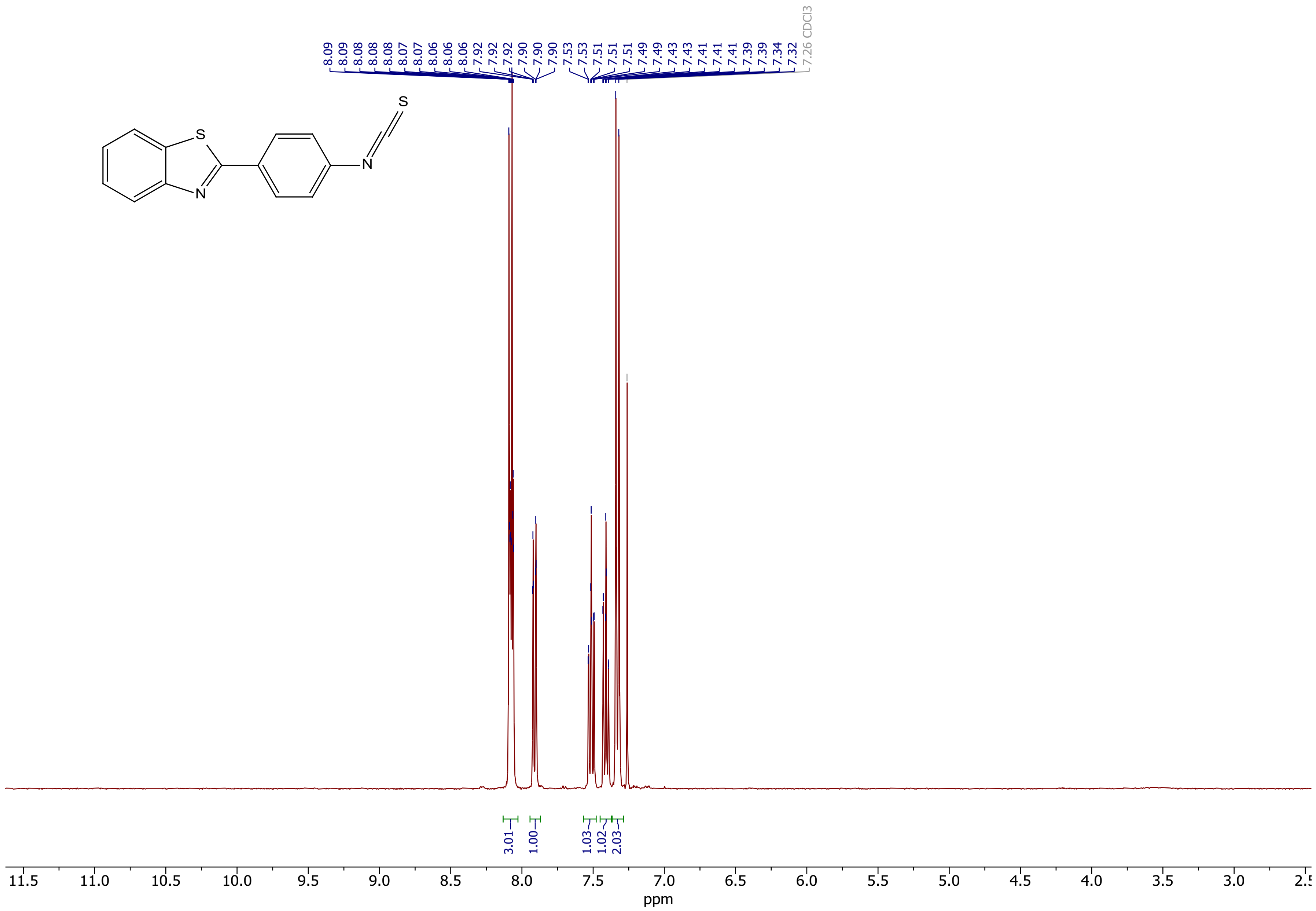

NMR ^{13}C *2-(4-isothiocyanatophenyl)benzo[d]thiazole* (**10**).

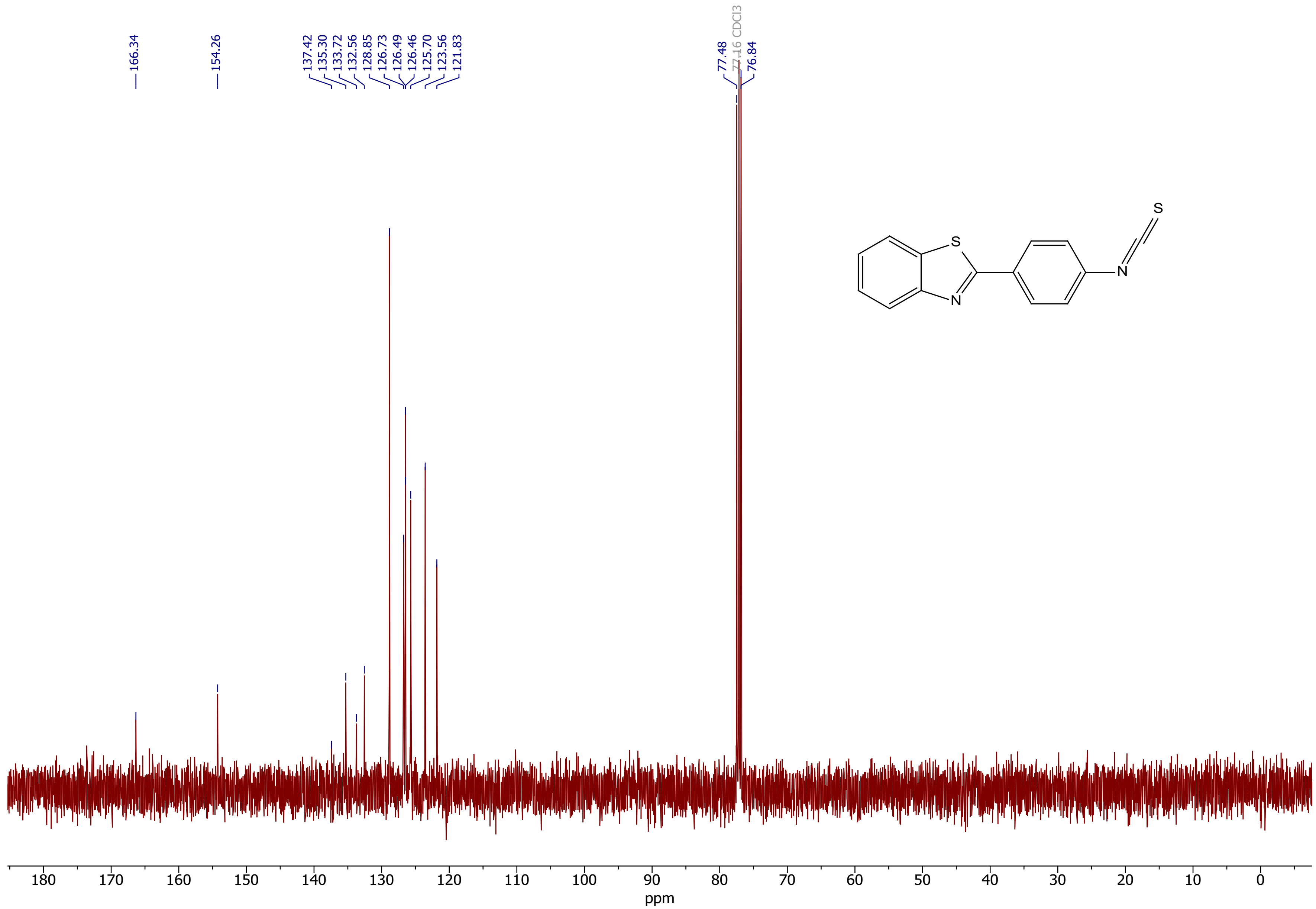

FTIR *2-(4-isothiocyanatophenyl)benzo[d]thiazole* (**10**).

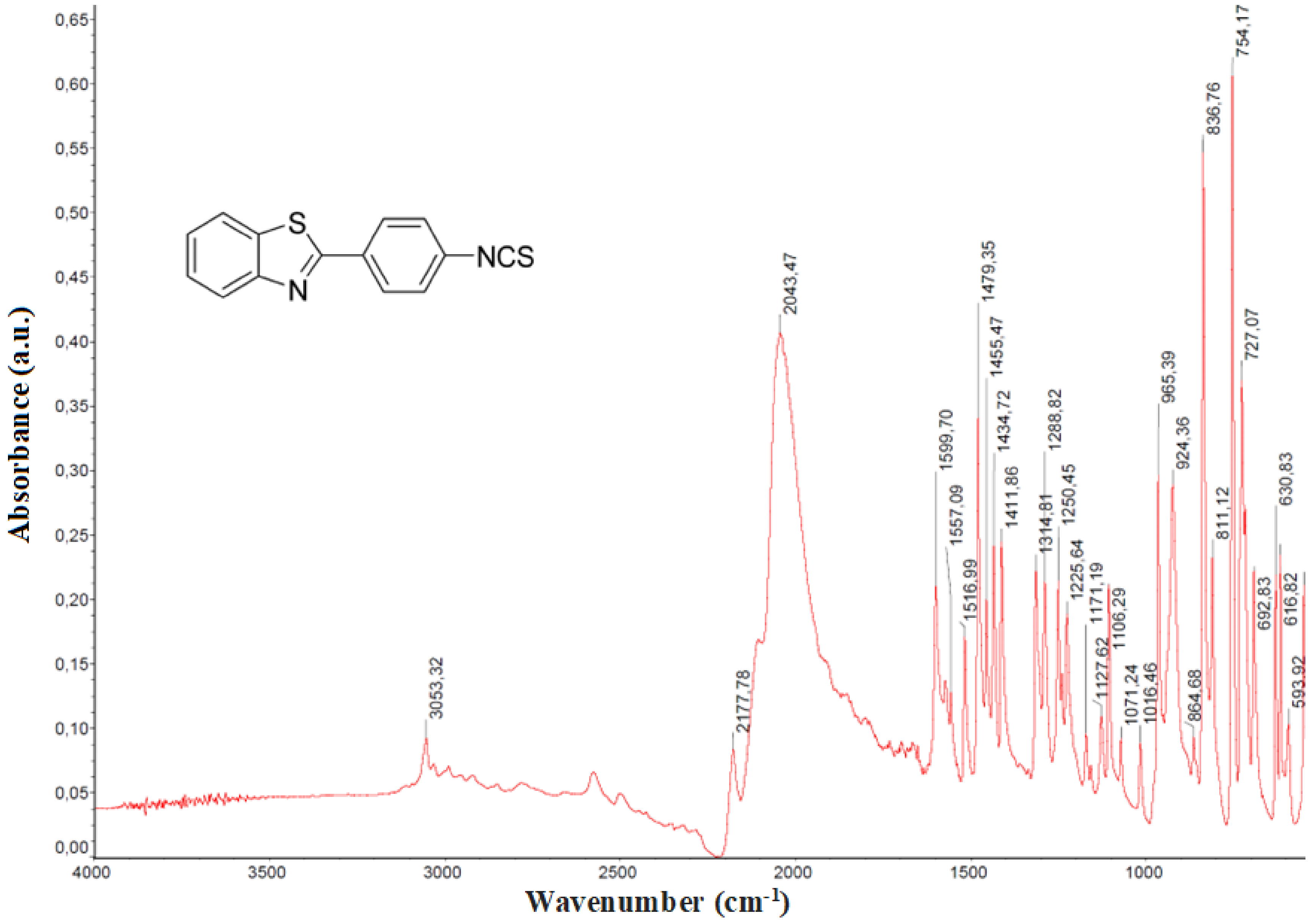

HRMS *2-(4-isothiocyanatophenyl)benzo[d]thiazole* (**10**).

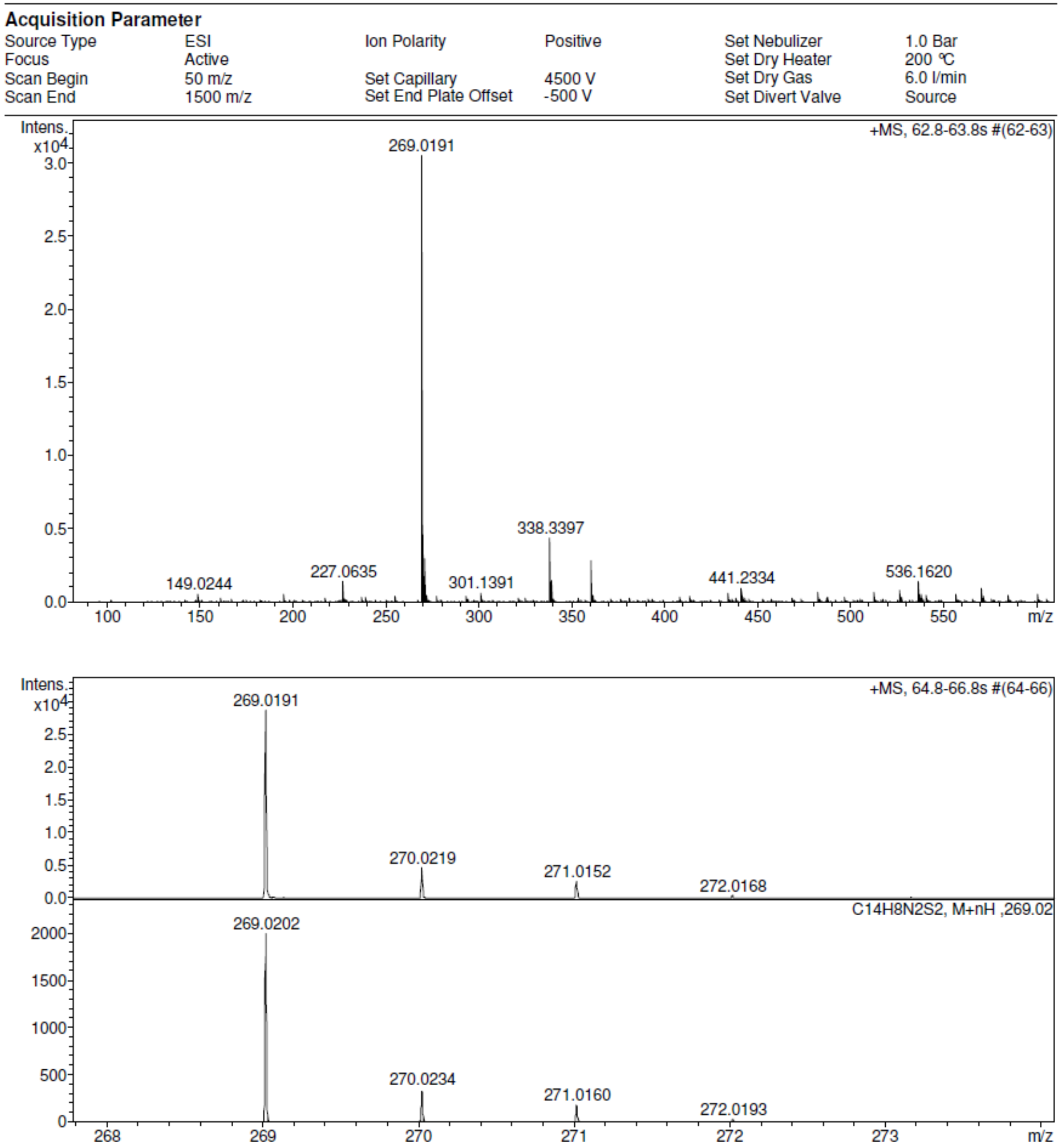

NMR ^{1}H *(E)-N-allyl-2-((E)-3-(2-((4-(benzo[d]thiazol-2-yl)phenyl)carbamothioyl)hydrazineylidene)butan-2-ylidene)hydrazine-1-carbothioamide* (**L8**).

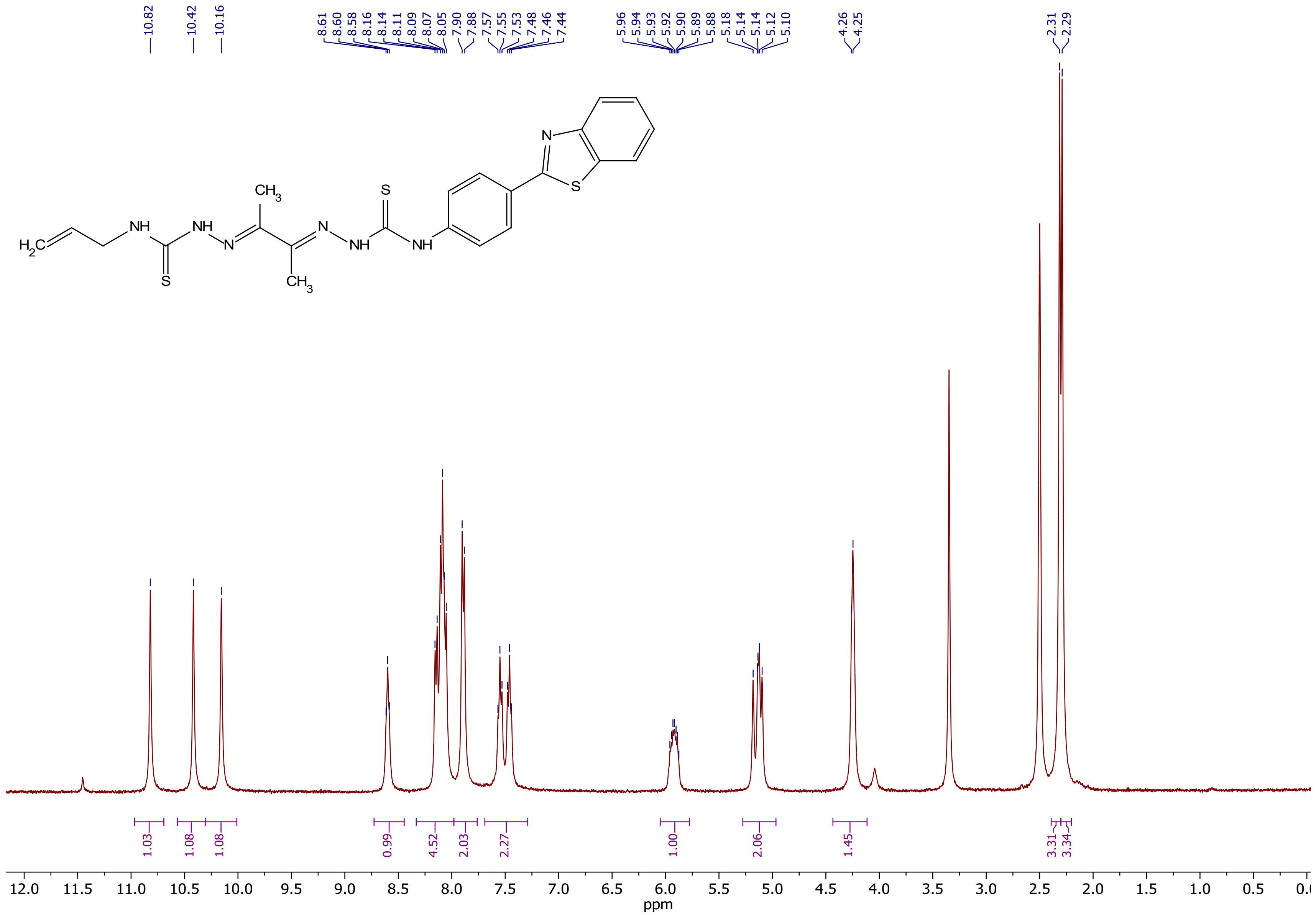

NMR ^{13}C *(E)-N-allyl-2-((E)-3-(2-((4-(benzo[d]thiazol-2-yl)phenyl)carbamothioyl)hydrazineylidene)butan-2-ylidene)hydrazine-1-carbothioamide* (**L8**).

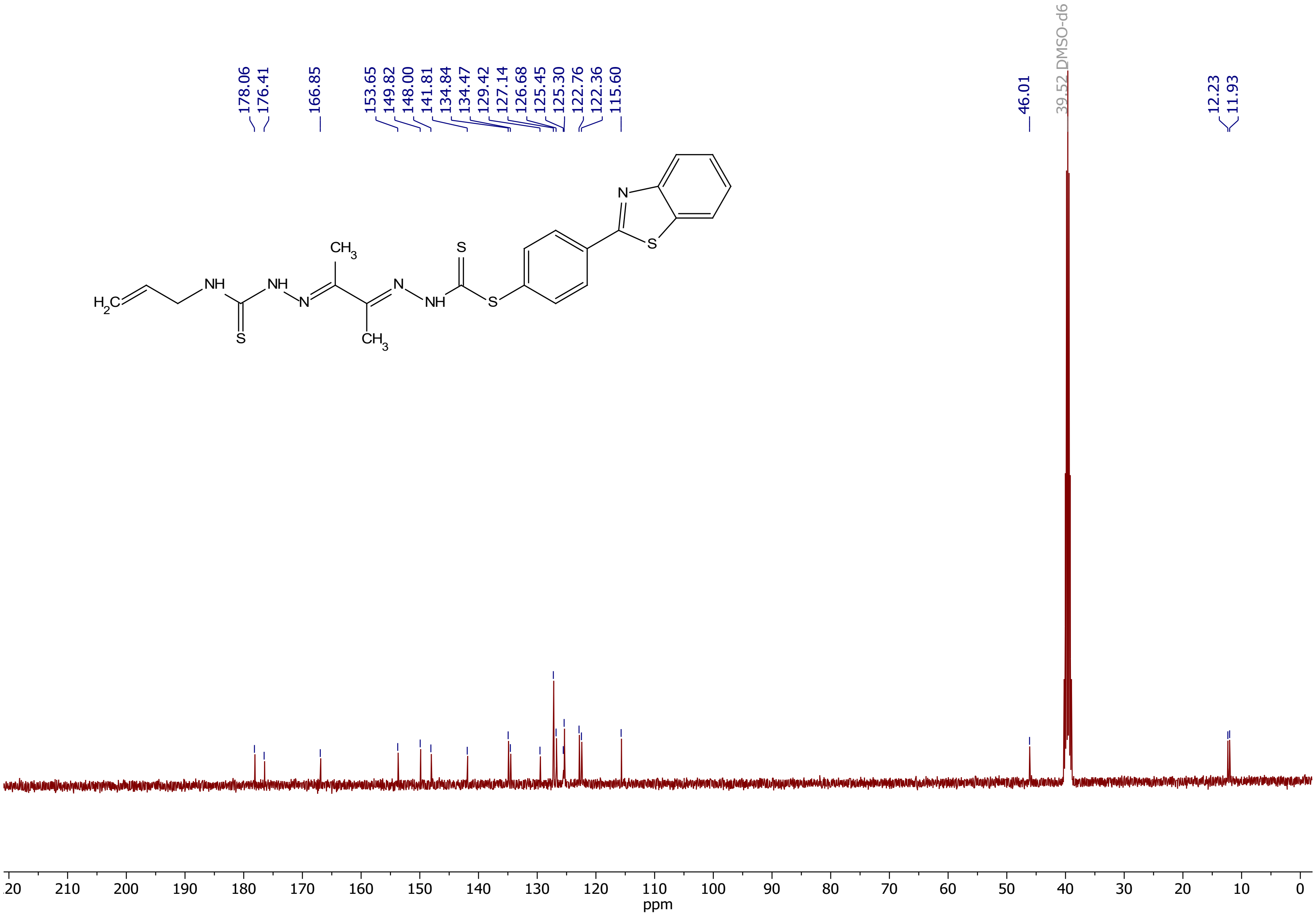

FTIR *(E)-N-allyl-2-((E)-3-(2-((4-(benzo[d]thiazol-2-yl)phenyl)carbamothioyl)hydrazineylidene)butan-2-ylidene)hydrazine-1-carbothioamide* (**L8**).

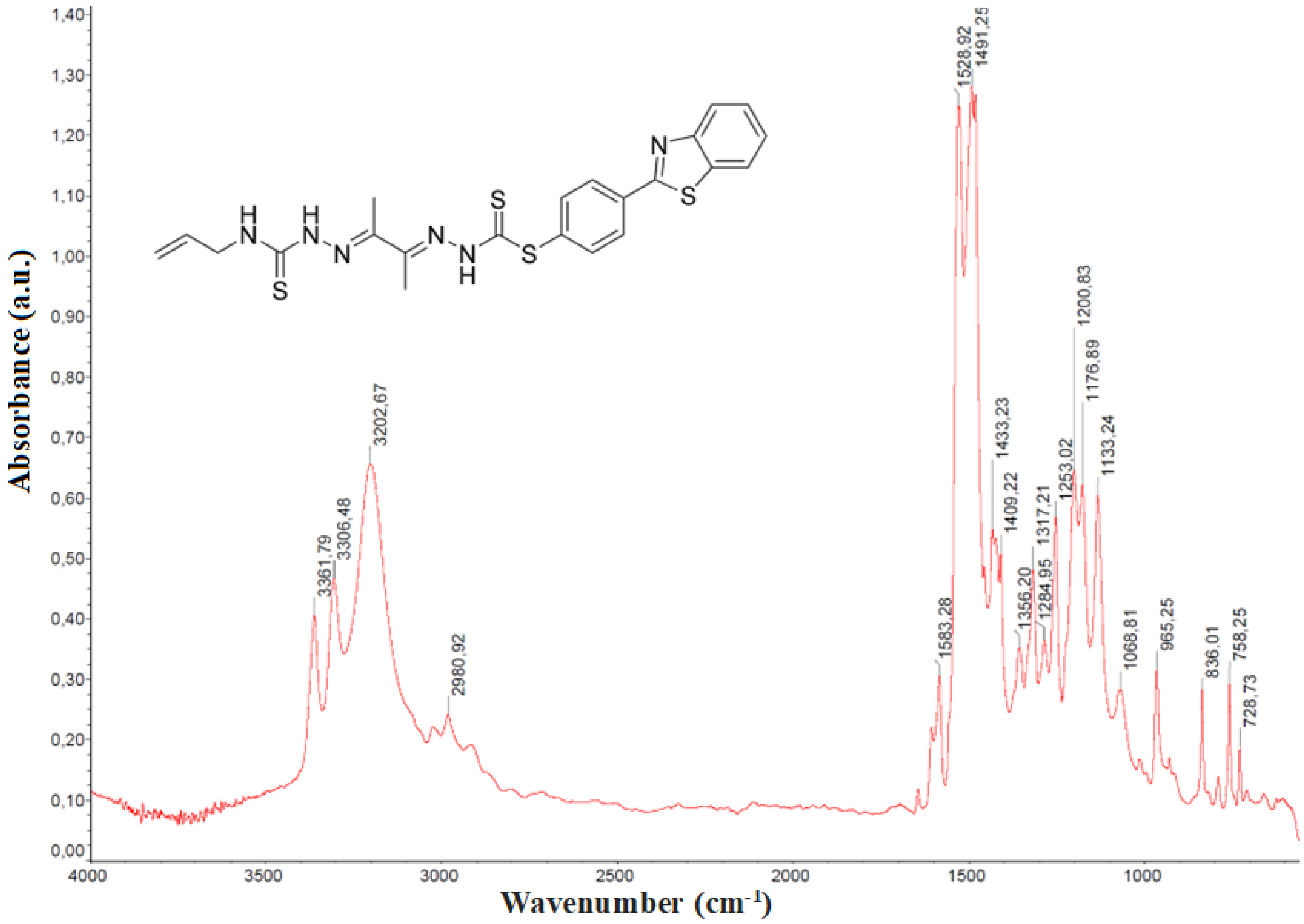

HRMS *(E)-N-allyl-2-((E)-3-(2-((4-(benzo[d]thiazol-2-yl)phenyl)carbamothioyl)hydrazineylidene)butan-2-ylidene)hydrazine-1-carbothioamide* (**L8**).

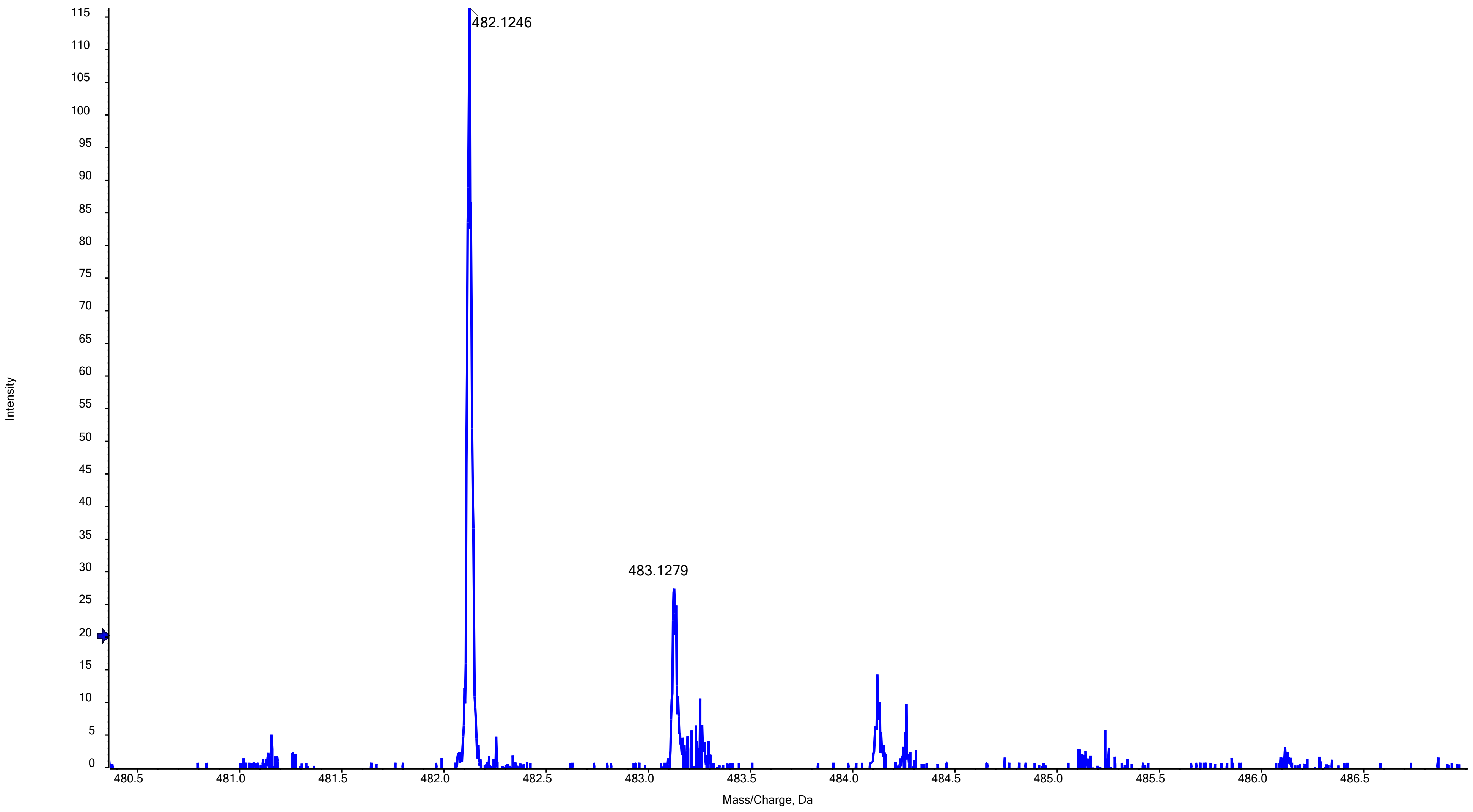

FTIR (**C6**).

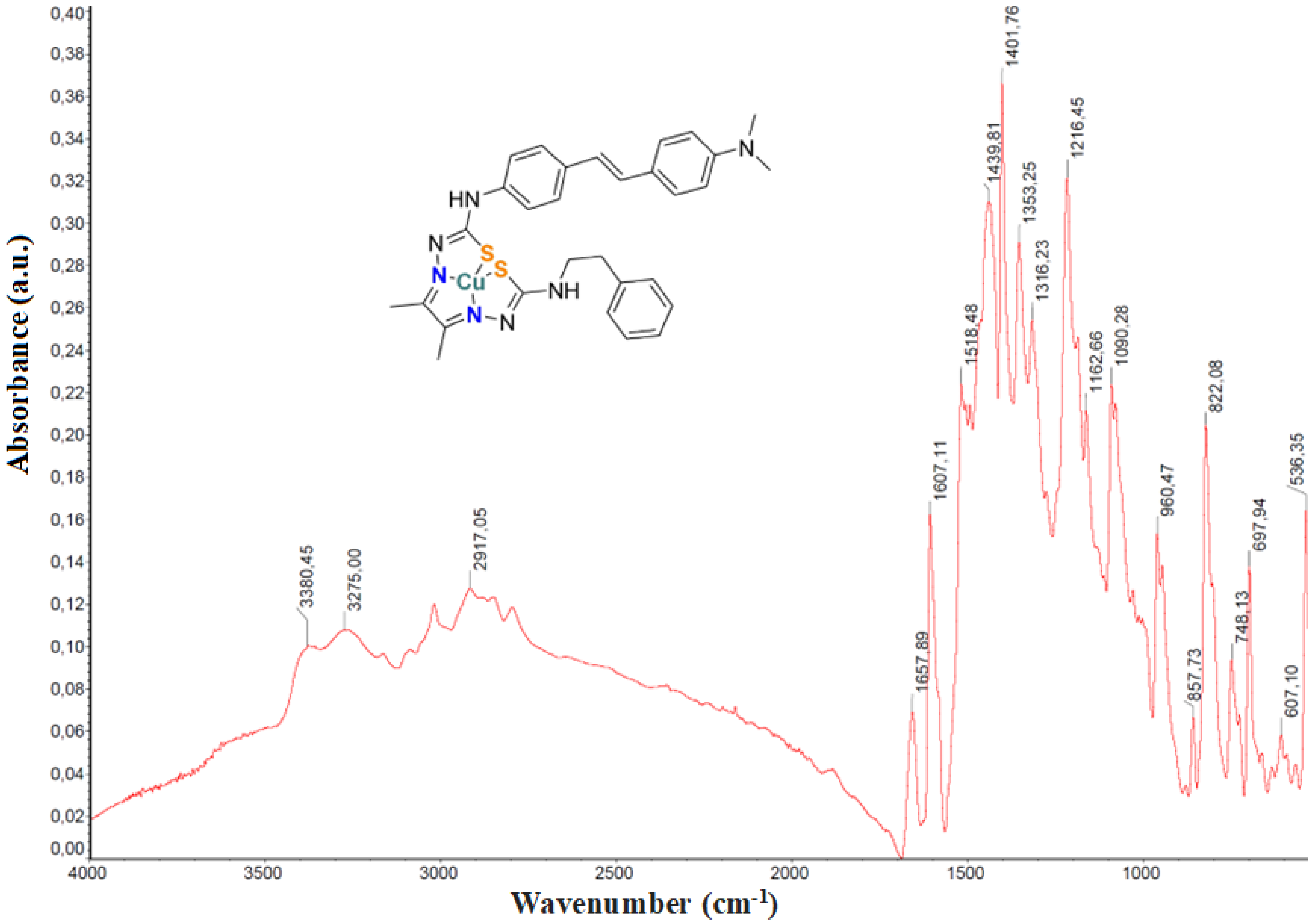

Absorbance (a.u.)
Wavenumber (cm-1)
0,40
0,38
0,36
0,34
0,32
0,30
0,28
0,26
0,24
0,22
0,20
0,18
0,16
0,14
0,12
0,10
0,08
0,06
0,04
0,02
0,00
4000
3500
3000
2500
2000
1500
1000
3380,45
3275,00
2917,05
1657,89
1607,11
1518,48
1439,81
1401,76
1353,25
1316,23
1216,45
1162,66
1090,28
960,47
857,73
822,08
748,13
697,94
607,10
536,35
HN
N
N
Cu
S
S
NH
N
N
N

FTIR (**C7**).

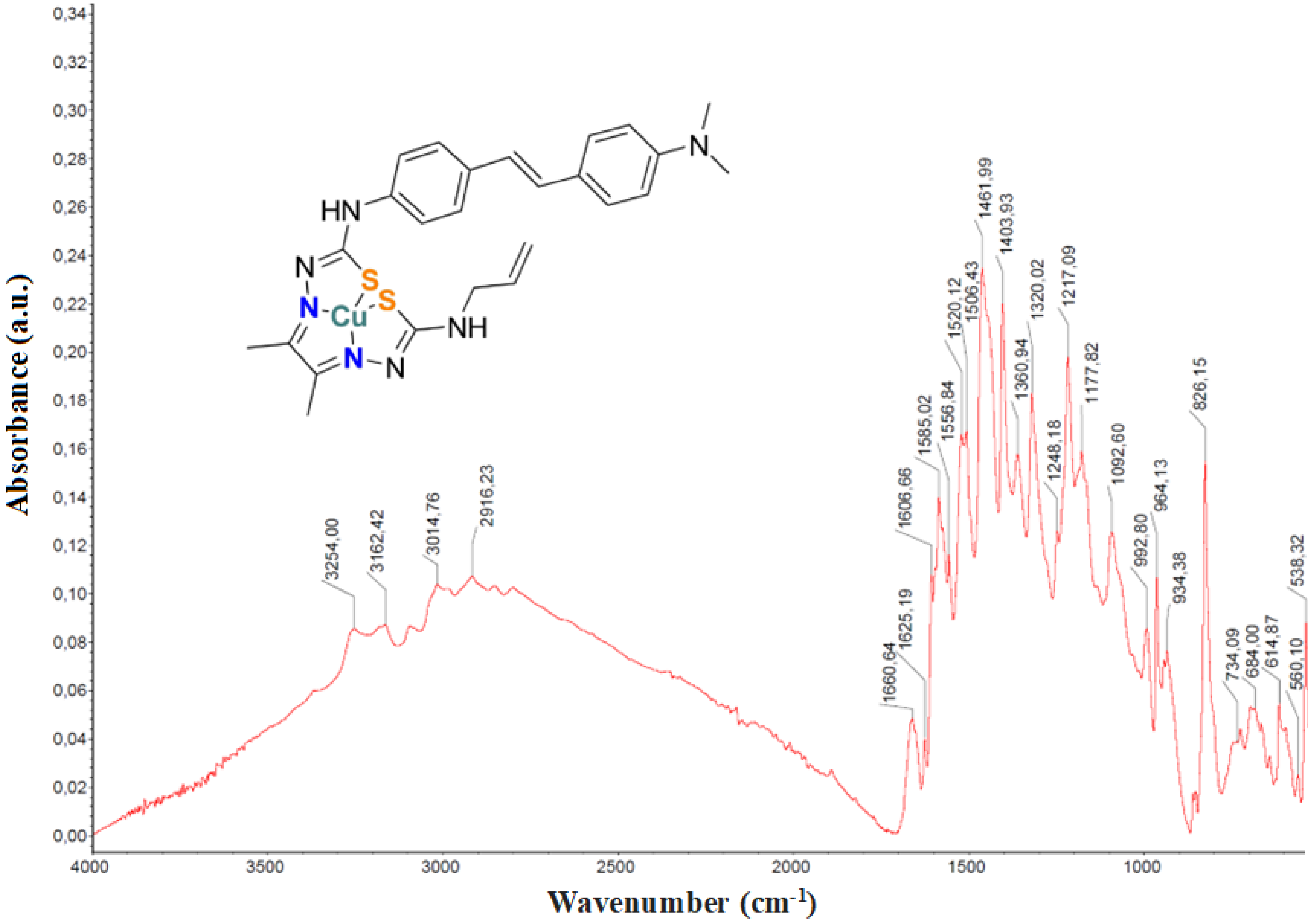

Absorbance (a.u.)
Wavenumber (cm-1)
0,00
0,02
0,04
0,06
0,08
0,10
0,12
0,14
0,16
0,18
0,20
0,22
0,24
0,26
0,28
0,30
0,32
0,34
4000
3500
3000
2500
2000
1500
1000
3254,00
3162,42
3014,76
2916,23
1660,64
1625,19
1606,66
1585,02
1556,84
1520,12
1506,43
1461,99
1403,93
1360,94
1320,02
1248,18
1217,09
1177,82
1092,60
992,80
964,13
934,38
826,15
734,09
684,00
614,87
560,10
538,32
HN
NH
N
S
Cu

FTIR (**C8**).

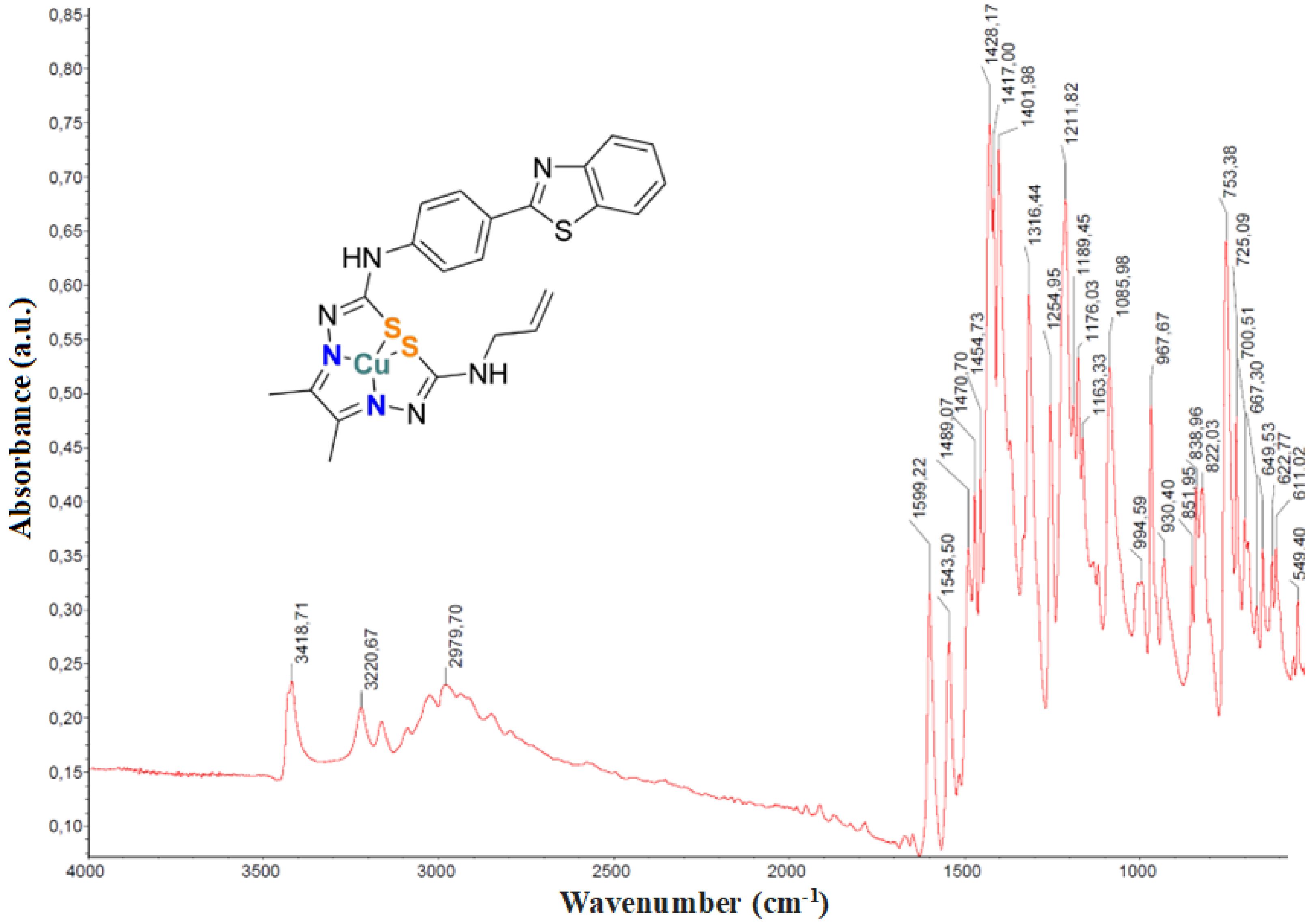
Absorbance (a.u.)
Wavenumber (cm-1)
3418,71
3220,67
2979,70
1599,22
1543,50
1489,07
1470,70
1454,73
1428,17
1417,00
1401,98
1316,44
1254,95
1211,82
1189,45
1176,03
1163,33
1085,98
994,59
967,67
930,40
851,95
838,96
822,03
753,38
725,09
700,51
667,30
649,53
622,77
611,02
549,40
HN
N
S
NH
Cu

MALDI (**C6**).

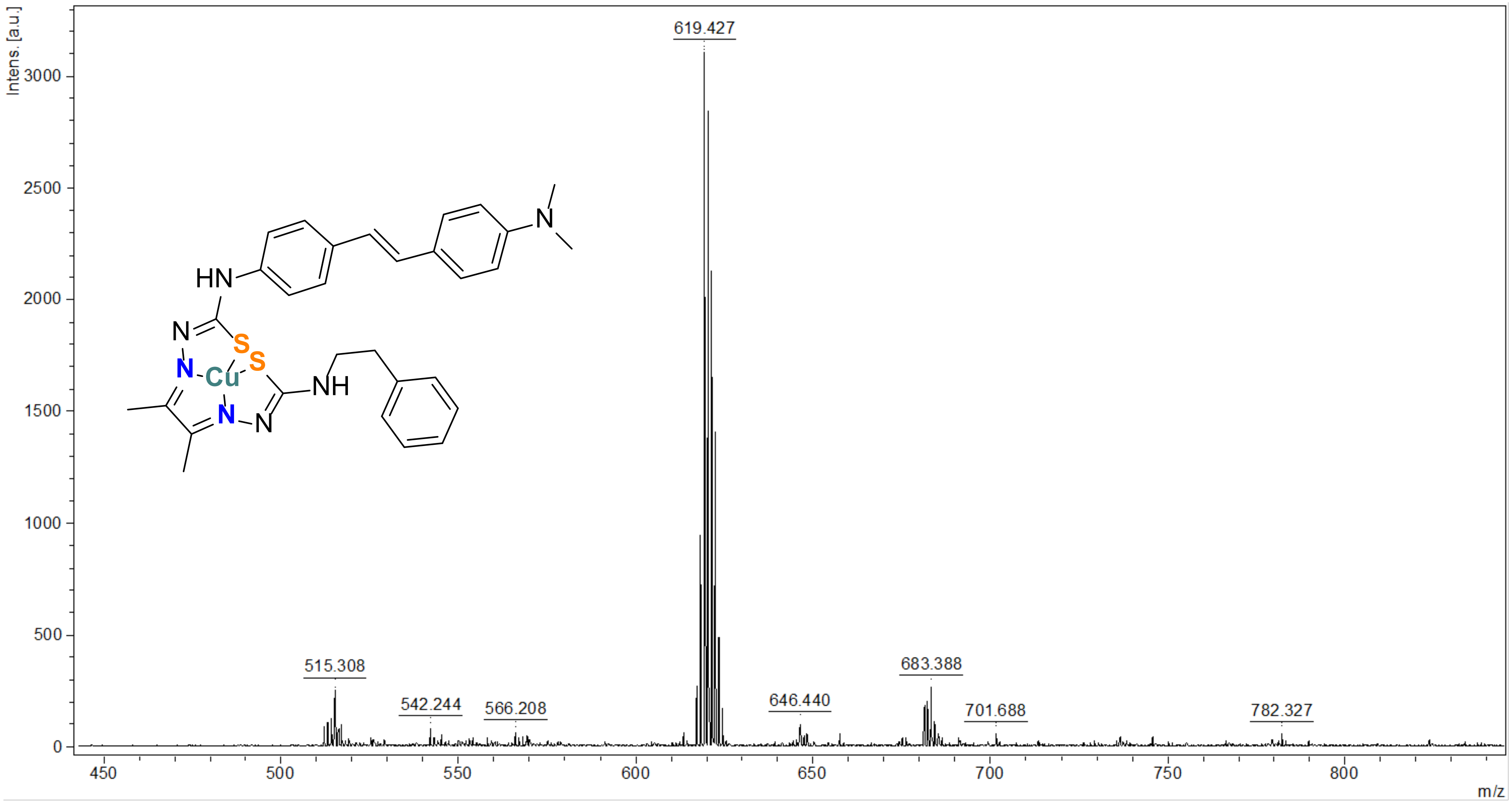
Intens. [a.u.]
3000
2500
2000
1500
1000
500
0
619.427
515.308
542.244
566.208
646.440
683.388
701.688
782.327
450
500
550
600
650
700
750
800
m/z
HN
N
N
Cu
S
S
NH
N
N
N

MALDI (**C7**).

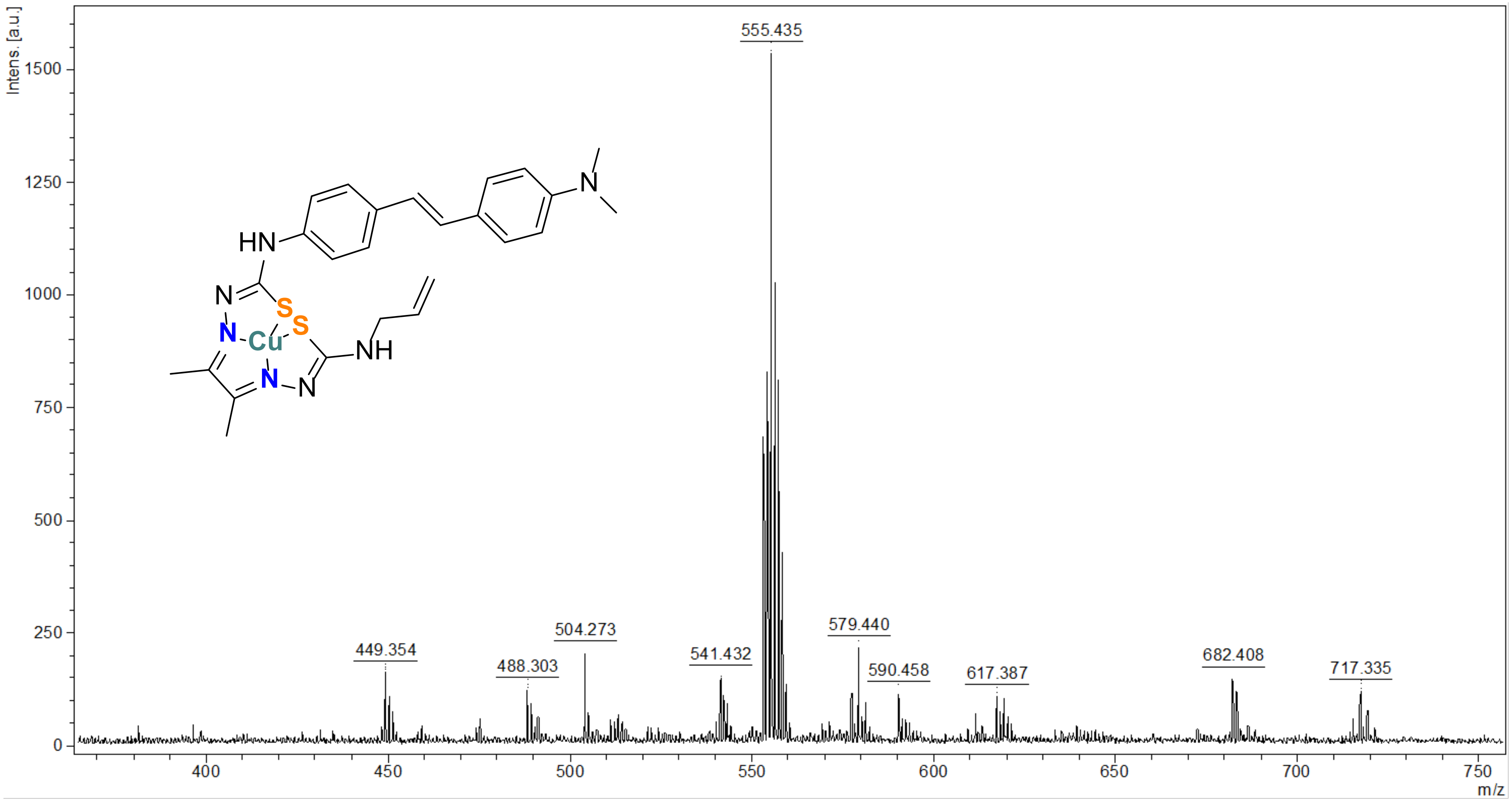
Intens. [a.u.]
1500
1250
1000
750
500
250
0
555.435
449.354
488.303
504.273
541.432
579.440
590.458
617.387
682.408
717.335
400
450
500
550
600
650
700
750
m/z
HN
N
S
S
N
Cu
N
N
NH
N

MALDI (**C8**).

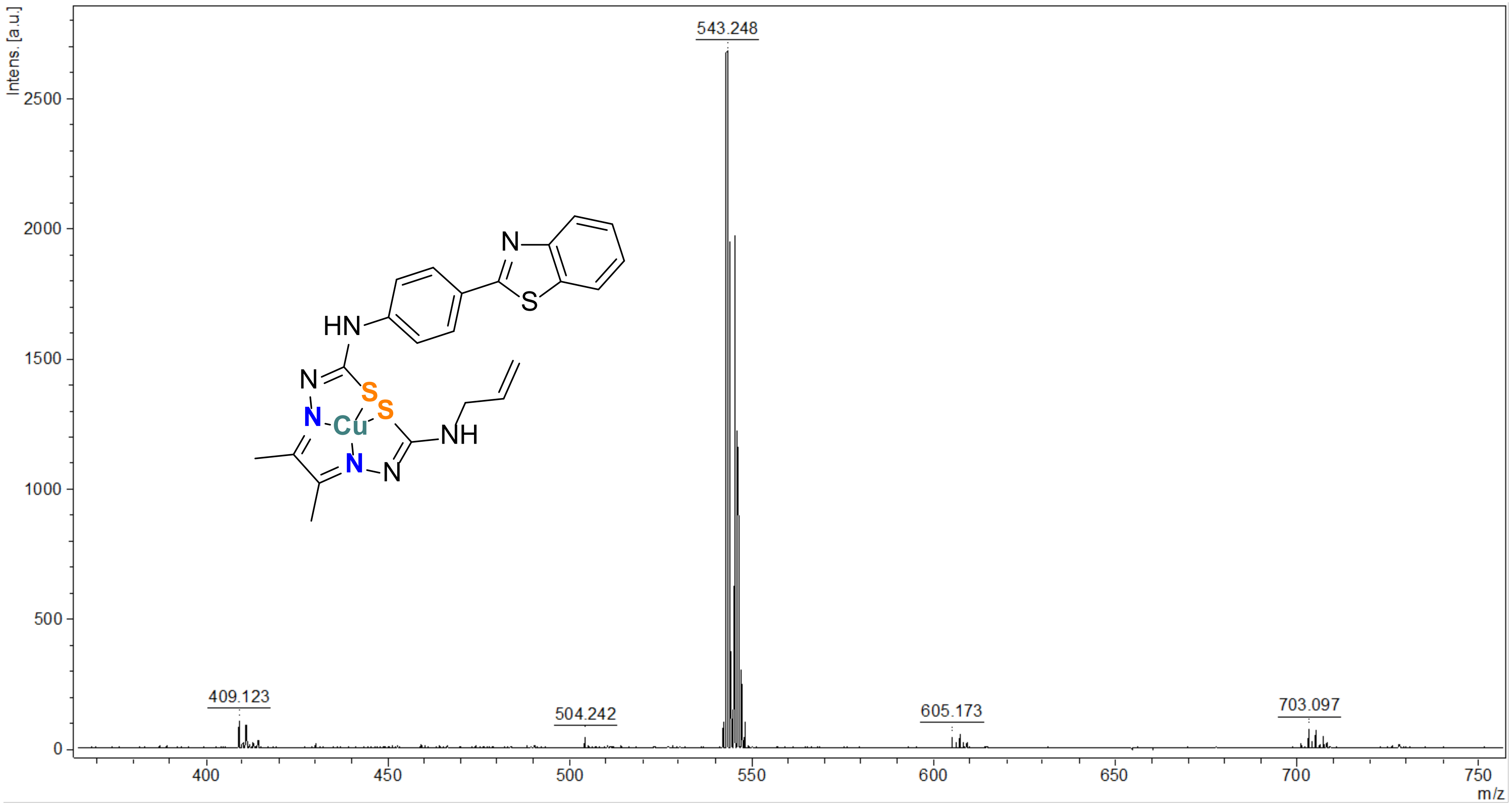

Intens. [a.u.]
2500
2000
1500
1000
500
0
543.248
409.123
504.242
605.173
703.097
400
450
500
550
600
650
700
750
m/z
N
S
HN
N
S
S
N
Cu
N
N
NH

Table S1. Crystal data and structure refinement for compound **7**.

Identificationcode	CCDC 2267583	
Empiricalformula	$C_8H_{13}N_3OS$	
Formulaweight	199.27	
Temperature	295(2) K	
Wavelength	1.54186 Å	
Crystalsystem	Monoclinic	
Spacegroup	P 21/n	
Unitcelldimensions	a = 6.9332(2) Å<br>b = 17.5783(5) Å<br>c = 8.6122(3) Å	α = 90°.<br>β = 90.300(3)°.<br>γ = 90°.
Volume	1049.59(6) Å^3	
Z	4	
Density (calculated)	1.261 Mg/m^3	
Absorptioncoefficient	2.485 mm^{-1}	
F(000)	424	
Theta range for data collection	5.032 to 66.764°.	
Indexranges	-8<=h<=7, -10<=k<=20, -10<=l<=10	
Reflectionscollected	7192	
Independentreflections	1837 [R(int) = 0.0468]	
Completenesstotheta = 66.764°	98.4 %	
Refinementmethod	Full-matrix least-squares on F^2	
Data / restraints / parameters	1837 / 0 / 145	
Goodness-of-fit on F^2	0.994	
Final R indices [I>2sigma(I)]	R1 = 0.0387, wR2 = 0.0977	
R indices (alldata)	R1 = 0.0519, wR2 = 0.1033	
Extinctioncoefficient	0.0048(8)	
Largest diff. peak and hole	0.278 and -0.319 e.Å^{-3}	

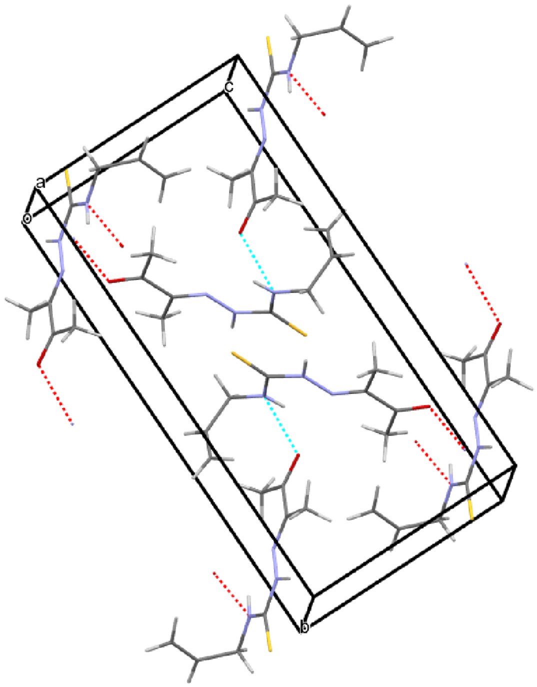

Figure S1. The unit cell for (**7**).

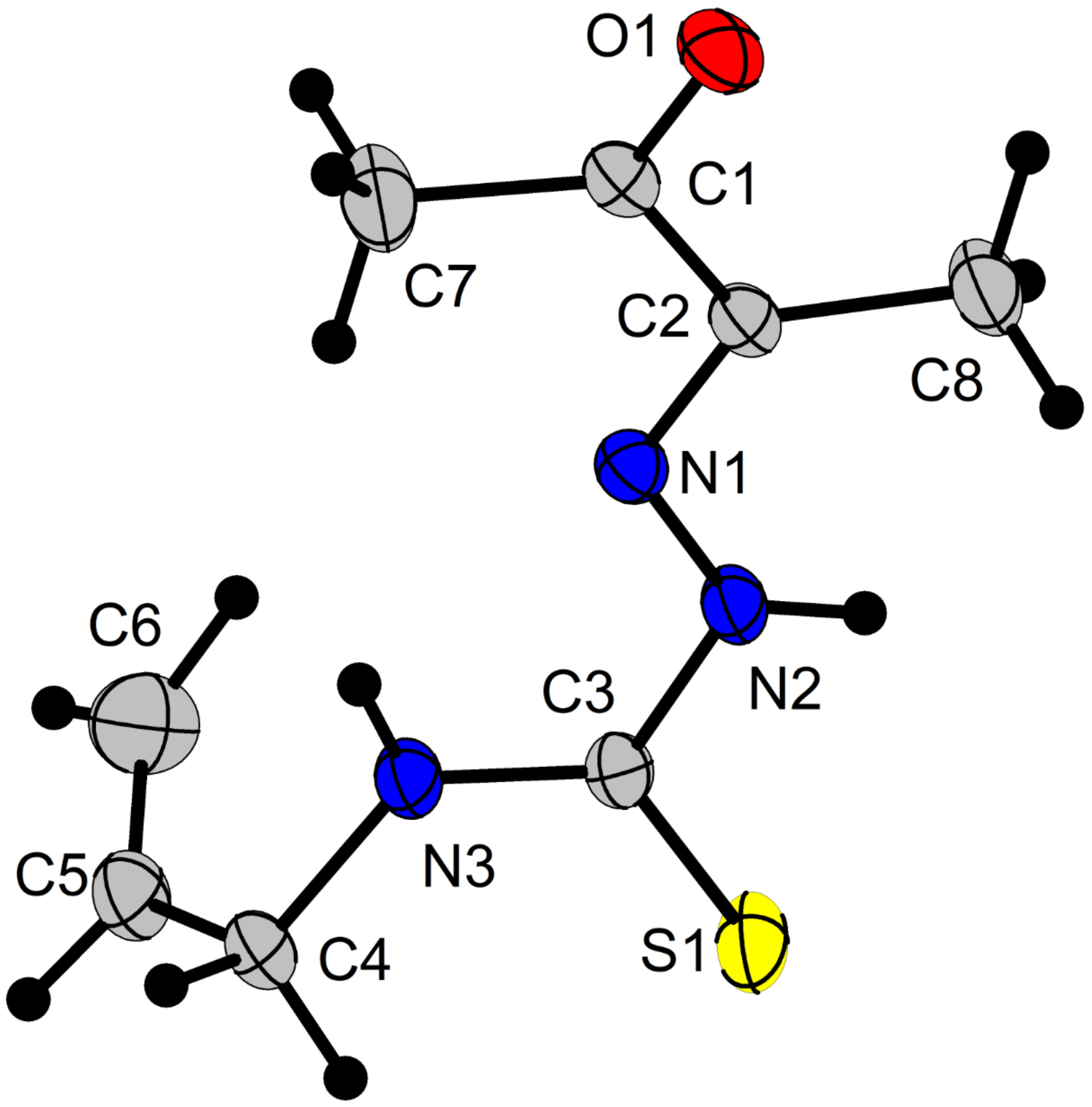

Figure S2. Crystal structure of (**7**); thermal ellipsoids are at the 50% level.

Table S2. Hydrogen bonds for (**7**) [Å and °].

D-H...A	d(D-H)	d(H...A)	d(D...A)	<(DHA)
C(8)-H(8A)...S(1)#1	0.96	2.85	3.420(2)	118.8
N(2)-H(2)...S(1)#1	0.80(2)	2.83(2)	3.6181(18)	169.1(19)
N(3)-H(3)...O(1)#2	0.83(2)	2.31(2)	2.973(2)	137.5(19)

Symmetry transformations used to generate equivalent atoms: #1 -x+2, -y+1, -z+1; #2 x-1/2, -y+3/2, z-1/2.

Table S3. Bond lengths [Å] and angles [°] for (**7**).

S(1)-C(3)	1.6675(19)
O(1)-C(1)	1.216(2)
N(1)-C(2)	1.288(2)
N(1)-N(2)	1.350(2)
N(2)-C(3)	1.378(2)
N(2)-H(2)	0.80(2)
N(3)-C(3)	1.320(2)
N(3)-C(4)	1.456(2)
N(3)-H(3)	0.83(2)
C(1)-C(2)	1.490(3)
C(1)-C(7)	1.495(3)
C(2)-C(8)	1.492(2)
C(4)-C(5)	1.482(3)
C(4)-H(41)	0.96(2)
C(4)-H(42)	0.97(2)
C(5)-C(6)	1.280(4)
C(5)-H(5)	0.9300
C(6)-H(61)	0.93(3)
C(6)-H(62)	0.98(3)
C(7)-H(7A)	0.9600
C(7)-H(7B)	0.9600
C(7)-H(7C)	0.9600
C(8)-H(8A)	0.9600
C(8)-H(8B)	0.9600
C(8)-H(8C)	0.9600
C(2)-N(1)-N(2)	118.67(14)
N(1)-N(2)-C(3)	120.01(15)
N(1)-N(2)-H(2)	124.2(15)
C(3)-N(2)-H(2)	114.8(15)
C(3)-N(3)-C(4)	124.74(18)
C(3)-N(3)-H(3)	117.3(15)
C(4)-N(3)-H(3)	117.7(15)
O(1)-C(1)-C(2)	119.48(16)
O(1)-C(1)-C(7)	120.54(18)
C(2)-C(1)-C(7)	119.98(16)
N(1)-C(2)-C(1)	115.55(15)
N(1)-C(2)-C(8)	125.44(17)
C(1)-C(2)-C(8)	118.97(15)
N(3)-C(3)-N(2)	115.62(17)
N(3)-C(3)-S(1)	125.46(14)
N(2)-C(3)-S(1)	118.92(13)
N(3)-C(4)-C(5)	114.95(18)
N(3)-C(4)-H(41)	107.6(12)
C(5)-C(4)-H(41)	109.2(11)
N(3)-C(4)-H(42)	106.9(13)
C(5)-C(4)-H(42)	111.6(13)
H(41)-C(4)-H(42)	106.1(19)
C(6)-C(5)-C(4)	128.2(2)
C(6)-C(5)-H(5)	115.9
C(4)-C(5)-H(5)	115.9
C(5)-C(6)-H(61)	120.5(19)
C(5)-C(6)-H(62)	123.6(16)
H(61)-C(6)-H(62)	116(3)
C(1)-C(7)-H(7A)	109.5
C(1)-C(7)-H(7B)	109.5
H(7A)-C(7)-H(7B)	109.5
C(1)-C(7)-H(7C)	109.5
H(7A)-C(7)-H(7C)	109.5
H(7B)-C(7)-H(7C)	109.5
C(2)-C(8)-H(8A)	109.5
C(2)-C(8)-H(8B)	109.5
H(8A)-C(8)-H(8B)	109.5
C(2)-C(8)-H(8C)	109.5
H(8A)-C(8)-H(8C)	109.5
H(8B)-C(8)-H(8C)	109.5

Table S4. Torsion angles [°] for (**7**).

C(2)-N(1)-N(2)-C(3)	176.57(16)
N(2)-N(1)-C(2)-C(1)	-177.74(15)
N(2)-N(1)-C(2)-C(8)	0.1(3)
O(1)-C(1)-C(2)-N(1)	172.90(17)
C(7)-C(1)-C(2)-N(1)	-6.1(3)
O(1)-C(1)-C(2)-C(8)	-5.1(3)
C(7)-C(1)-C(2)-C(8)	175.9(2)
C(4)-N(3)-C(3)-N(2)	-174.75(18)
C(4)-N(3)-C(3)-S(1)	5.0(3)
N(1)-N(2)-C(3)-N(3)	1.3(2)
N(1)-N(2)-C(3)-S(1)	-178.53(13)
C(3)-N(3)-C(4)-C(5)	-97.6(2)
N(3)-C(4)-C(5)-C(6)	4.3(4)

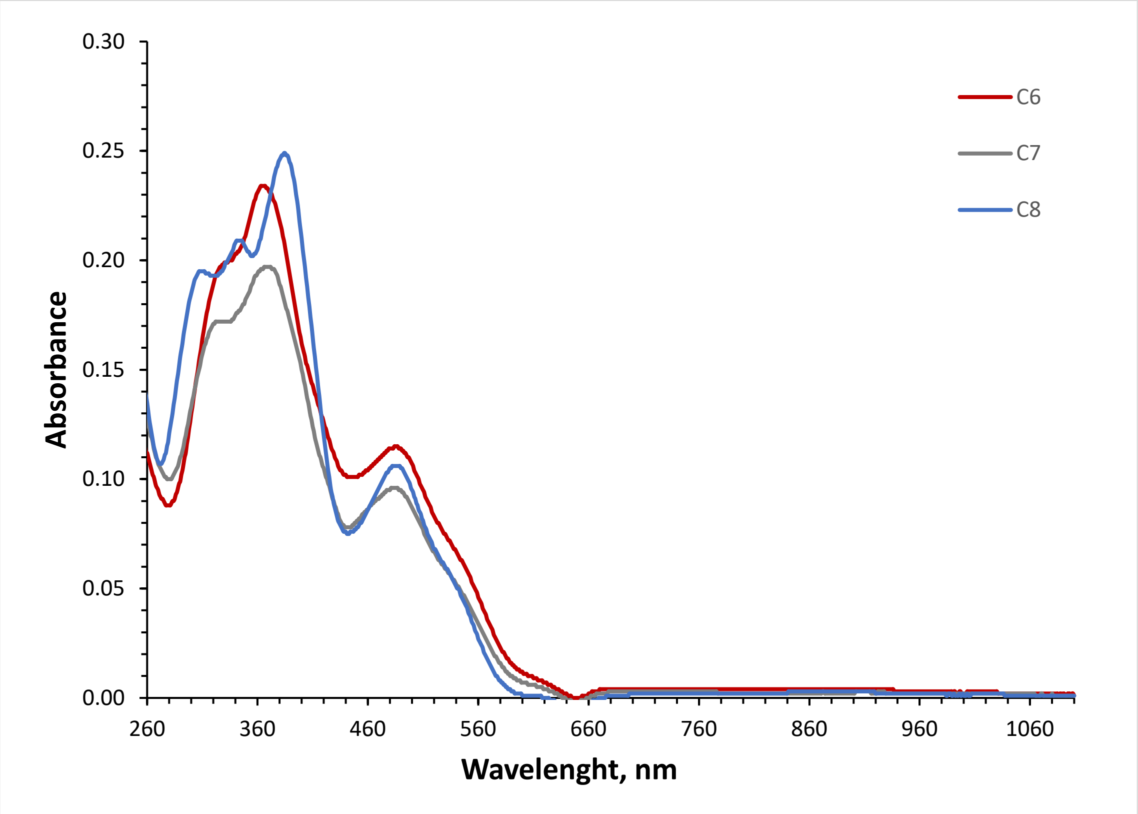

Figure S3. UV–Vis spectra of complexes **C6–C8** (5×10^{-5} M) in DMF solution.

Note several bands in the 260–660 nm region. Maximum absorption was detectable at 365 nm and 485 nm for **C6**, 369 nm and 485 nm for **C7** and 384 nm and 485 nm for **C8**.

Table S5. Calculated IC_{50} values (μM) for the copper–organic complexes.

	Compound	Cell lines			
		HCT116		K562	
		no NAC	NAC	no NAC	NAC
Group 1	C1	2.1	~0.1	1.6	ND
	C2	11.7	~0.1	11.6	ND
	C3	5.8	~0.2	10.5	ND
	C4	27.7	1.1	5.6	ND
	C5	50.3	0.9	8.4	ND
Group 2	C6	48.7	11.6	44.3	2.1
	C7	12.1	5.2	12.9	1.4
	C8	1.4	0.3	21.0	10.2
Group 3	C9	NT	NT	NT	NT
	C10	NT	NT	NT	NT
	C11	NT	NT	NT	NT

Values are the mean of 3 independent measurements with ~10% error.
ND, not determined; the addition of NAC dramatically dropped K562 cell survival to the level below detection. NT, no cytotoxicity up to 100 μM.
